# Understanding gas capacity, guest selectivity, and diffusion in porous liquids†
†Electronic supplementary information (ESI) available: Detailed synthetic procedures, experimental details and measurements (PDF). See DOI: 10.1039/c6sc05196k



**DOI:** 10.1039/c6sc05196k

**Published:** 2017-01-31

**Authors:** Rebecca L. Greenaway, Daniel Holden, Edward G. B. Eden, Andrew Stephenson, Chin W. Yong, Michael J. Bennison, Tom Hasell, Michael E. Briggs, Stuart L. James, Andrew I. Cooper

**Affiliations:** a Department of Chemistry and Materials Innovation Factory , University of Liverpool , Crown Street , Liverpool , L69 7ZD , UK . Email: aicooper@liverpool.ac.uk; b Scientific Computing Department , Science and Technologies Facilities Council , Daresbury Laboratory , Sci-Tech Daresbury , Warrington , WA4 4AD , UK; c Manchester Pharmacy School , Faculty of Medical and Human Sciences , Manchester Academic Health Science Centre , University of Manchester , Manchester , M13 9NT , UK; d School of Chemistry and Chemical Engineering , Queen's University Belfast , David Keir Building, Stranmillis Road , Belfast , BT9 5AG , UK

## Abstract

Porous liquids are a new class of material that could have applications in areas such as gas separation and homogeneous catalysis. Here we use a combination of measurement techniques, molecular simulations, and control experiments to advance the quantitative understanding of these liquids. In particular, we show that the cage cavities remain unoccupied in the absence of a suitable guest, and that the liquids can adsorb large quantities of gas, with gas occupancy in the cages as high as 72% and 74% for Xe and SF_6_, respectively. Gases can be reversibly loaded and released by using non-chemical triggers such as sonication, suggesting potential for gas separation schemes. Diffusion NMR experiments show that gases are in dynamic equilibrium between a bound and unbound state in the cage cavities, in agreement with recent simulations for related porous liquids. Comparison with gas adsorption in porous organic cage solids suggests that porous liquids have similar gas binding affinities, and that the physical properties of the cage molecule are translated into the liquid state. By contrast, some physical properties are different: for example, solid homochiral porous cages show enantioselectivity for chiral aromatic alcohols, whereas the equivalent homochiral porous liquids do not. This can be attributed to a loss of supramolecular organisation in the isotropic porous liquid.

## Abstract 2

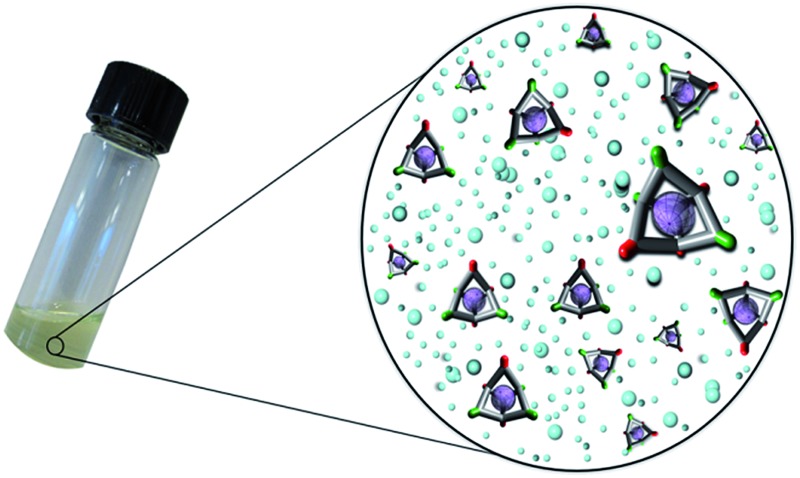
An in-depth study of porous liquids using measurement techniques, molecular simulations, and control experiments to advance their quantitative understanding.

## Introduction

The concept of a ‘porous liquid’—that is, a liquid containing permanent intrinsic cavities or pores—is still relatively new, having been first proposed by James *et al.* in 2007.^1^ Porous liquids should exhibit properties that are familiar for porous solids, such as enhanced gas uptakes and the potential for molecular selectivity, albeit perhaps with lower pore volumes. Such materials might have unique applications in the future: for example, liquids can be pumped around in a continuous system, which could facilitate guest loading and unloading steps.

There are few examples so far of porous liquids.^2–5^ Recently, we reported a Type 2 molecular porous liquid derived from a modified porous organic cage decorated with solubilising crown ether groups on the vertices. The cage was shown to be highly soluble in 15-crown-5, which was size excluded from the cage cavities.^5^ This porous liquid showed an 8-fold increase in methane solubility compared to the pure 15-crown-5 solvent. We also presented an alternative approach that uses dynamic covalent scrambling.^5,6^ When these scrambled cages are dissolved in a bulky solvent that is too large to enter the cage cavity, a free-flowing porous liquid is formed. These scrambled liquids had substantially lower viscosity than their crown-ether analogues and they also show enhanced gas uptakes and guest selectivity.^5^

Now that the basic concepts of porous liquids have been demonstrated, we need to understand these systems more fully to allow the design of the next generation of materials.^7,8^ For example, Qiao *et al.* recently studied the thermodynamics and kinetics of gas storage in crown-ether cage porous liquids by using molecular simulations.^9^ Here, we report the development of vertex disordered porous liquids, starting with the initial design strategy and extending to an in-depth study of the physical properties of the most porous liquid.

## Results and discussion

### Design strategy

The main challenge in producing a molecular porous liquid is to introduce a sufficient density of cavities while retaining fluidity and avoiding any cavity penetration. For Type 2 systems,^1^ which comprise a cage or macrocycle dissolved in a bulky solvent, this means that high solubilities of the cavity-containing molecule are required. This is a difficult challenge for porous organic cages,^10,11^ which often have modest solubilities (*e.g.*, **CC3**-*R* ^12^ has a maximum solubility of 0.2 wt% in chloroform at room temperature).

**Fig. 1:**
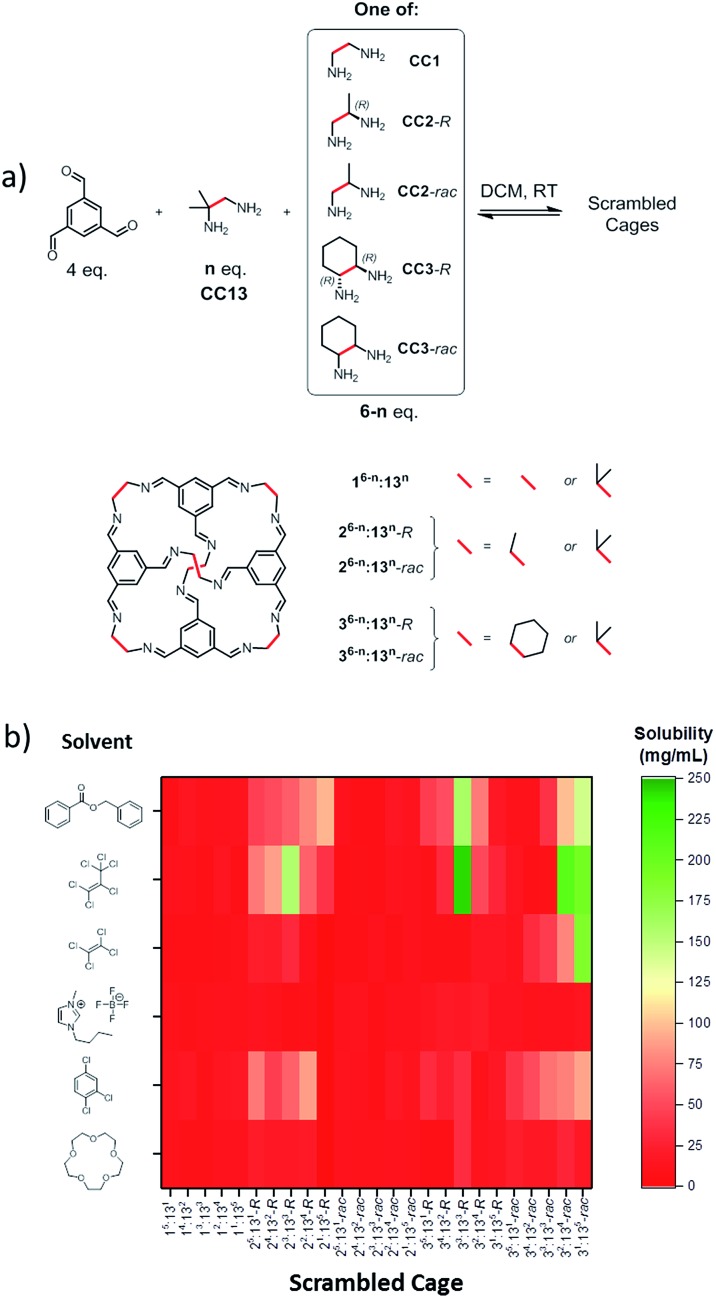
Scrambled cage synthesis and solubility testing for candidate Type 2 porous liquids. (a) Synthesis of the scrambled cages *x* ^6–*n*^:13^*n*^ *via* [4 + 6] imine condensations, where *n* represents the equivalents of the **CC13** diamine that were used and *x* represents the non-**CC13** diamine component; all possible whole number stoichiometric ratios were tested in each case (*i.e.*, 1 : 5, 2 : 4, 3 : 3, 4 : 2, and 5 : 1). (b) Plot showing the structures of the bulky solvents screened and the resulting solubilities of the scrambled cage mixtures. (rac = racemic diamine mixture; *R*- = *R*,*R*-homochiral diamine.) To provide a high density of cavities in the liquid, a high cage solubility is required (green areas in this plot).

Our scrambling approach^5^ was based on our previous observation that vertex-disordered cage mixtures are more soluble than cages derived from a single diamine.^6^ We attributed this to a reduction in lattice energy since these scrambled cage mixtures show a greatly reduced tendency to crystallise. Also, our most soluble, shape-persistent ‘unscrambled’ [4 + 6] cage to date is **CC13**,^13^ which has a solubility in chloroform of around 10 wt%. Our strategy, therefore, was to combine both effects and to generate cages with increased solubility by scrambling **CC13** with various vicinal diamines. A library containing 25 scrambled cage mixtures was synthesised by direct imine condensation of 1,3,5-triformylbenzene with varying ratios of 1,2-diamino-2-methylpropane (**CC13** component) and a second vicinal diamine (Fig. 1a).

### Solubility screening

A series of six different bulky solvents was selected, all of which were likely to be large enough to be size-excluded by the cage cavity or the cage windows. The solubility of the 25 different scrambled cages was tested in these bulky solvents to give a total of 150 combinations (Fig. 1b).

**Fig. 2:**
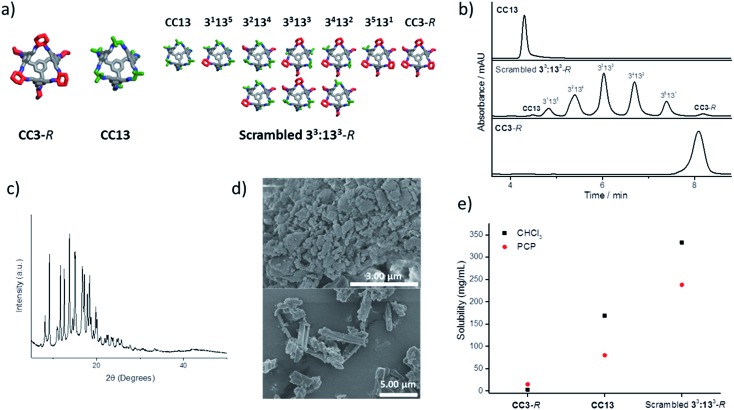
(a) Comparison of the ‘parent’ **CC3**-*R* and **CC13** cage structures with the scrambled **3^3^:13^3^**-*R* mixture; only the relative positional isomers of cyclohexane and dimethyl vertices are shown; the additional positional isomerism of the dimethyl vertices is not represented here. Cyclohexane vertices are highlighted in red, dimethyl vertices highlighted in green; hydrogens omitted for clarity; (b) analytical HPLC traces showing the product distribution formed from scrambling a 1 : 1 mixture of (*R*,*R*)-cyclohexanediamine (**CC3**-*R*) with 1,2-diamino-2-methylpropane (**CC13**); the positional isomers are not resolved under these separation conditions, and hence 7 peaks are observed; (c) powder X-ray diffraction pattern of the scrambled cage **3^3^:13^3^**-*R* showing a modest degree of crystallinity; (d) scanning electron micrographs show that the sample lacks the degree of geometric order that would be expected in a highly crystalline sample; (e) comparison of the solubility of **CC3**-*R*, **CC13**, and the scrambled **3^3^:13^3^**-*R* cage mixture in chloroform and in hexachloropropene (PCP), showing that scrambling has increased the cage solubility.

The solubility of the scrambled cage mixtures was first determined in chloroform; this solvent is small enough to enter the cage cavities, and it would not therefore be expected to form a Type 2 molecular porous liquid. In almost all cases, the scrambled cage mixtures were found to be more soluble in chloroform than pure **CC13** (see ESI Fig. 1†). However, with the bulky solvents, most combinations showed a low solubility of <30 mg mL^–1^ (Fig. 1b). A few materials reached solubilities of ∼200 mg mL^–1^, but one combination in particular proved to be exceptionally soluble: 1 : 1 (*R*,*R*)-1,2-cyclohexanediamine/1,2-diamino-2-methylpropane (Fig. 2a–d) using hexachloropropene (PCP) as the solvent. This mixture (Fig. 2a) gave reproducible solubilities of 234–242 mg of scrambled cage in 1 mL of PCP, equating to an approximate 1 : 31 molar ratio of cage to solvent. On comparing the solubility of the parent cages, **CC3**-*R* and **CC13**, it is clear that scrambling has increased the solubility substantially, both in chloroform and in PCP (Fig. 2e).

The trend in solubility (scrambled **3^3^:13^3^**-*R* > **CC13** > **CC3**-*R*) can be rationalised by the increasing disorder that has been introduced into the cages vertices (Fig. 2a). **CC3**-*R* is a highly crystalline single molecular species with no vertex disorder. Like **CC3**-*R*, **CC13** is also synthesised from a single diamine, but the asymmetric dimethyl vertices lead to a number of positional isomers, which increases the cage solubility. The scrambled **3^3^:13^3^**-*R* mixture contains both positional isomerism from the dimethyl vertices and from the two different diamines that are incorporated. Another factor that is likely to increase the solubility is the introduction of chirality in the **3^3^:13^3^**-*R* mixture. We showed previously that favourable interactions between racemic cages lead to a marked decrease in solubility.^14^ The **CC13** diamine is achiral, and the resulting cages are therefore racemic. However, introduction of the second diamine, (*R*,*R*)-cyclohexanediamine, strongly biases the chirality of the cage mixture, further increasing solubility by reducing favourable interactions between racemic cage pairs.

The powder X-ray diffraction pattern of this scrambled **3^3^:13^3^**-*R* cage mixture shows a degree of crystallinity, probably arising from selective crystallisation of some components in the cage mixture. However, the bulk of the material appears more amorphous in character in scanning electron micrographs (Fig. 2c and d), and the material does not show the usual crystal habit displayed by the parent cages, **CC3**-*R* and **CC13** (see ESI Fig. 7†).^13,14^ It is therefore likely that increased disorder reduces the ability of the cages to pack efficiently, which in turn reduces lattice energy and therefore increases solubility.

Having found a scrambled cage that is highly soluble in a bulky solvent, the cage concentration was set at 20% w_cage_/v_PCP_; that is, slightly below the saturation point to ensure reproducibility during testing. This equates to 10 wt% and a 1 : 35.7 molar ratio of cage to PCP solvent. We note here that the bulky solvent was thoroughly purified before use to avoid any smaller-sized impurities acting as a competitive guest in the cage cavities (ESI Fig. 12†). (Safety note: PCP is highly toxic and should be handled with adequate precautions.)

### Molecular simulations

**Fig. 3:**
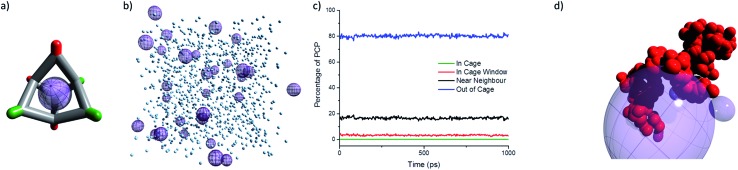
(a) Schematic representation of the scrambled **CC3^3^13^3^** cage used in the computational modelling showing the guest-accessible intrinsic cavity; cyclohexane vertices shown in red, dimethyl vertices shown in green; empty cage cavity shown as purple sphere; (b) molecular simulation of the scrambled porous liquid showing the available free space in the cages (purple spheres); PCP solvent molecules shown as pale blue spheres; cage molecules omitted for clarity; (c) percentage of PCP molecules found in the cage, in the cage window, as near neighbours, or in the bulk solvent over the duration of a 1000 ps molecular dynamics (MD) simulation, demonstrating that PCP solvent is excluded from the cage cavity; (d) pathway taken by a single PCP molecule during the MD simulation highlighting the close proximity to, but exclusion from, the cage cavity (purple sphere).

To be classified as a Type 2 porous liquid, the intrinsic cavities in the cages must be empty (Fig. 3a), and most of the free space in the porous liquid should be attributed to these cavities. While it is possible to infer this by indirect methods—for example, by demonstrating enhanced gas solubility^5^—this does not exclude the possibility that the cages are actually occupied by solvent, and that the gas displaces this solvent because it is a better guest. We therefore developed a computational model of the liquid, built using the experimental 1 : 36 cage to PCP solvent ratio (Fig. 3b). The diffusion of the PCP solvent was then monitored in a molecular dynamics (MD) simulation. This MD simulation showed that PCP moves towards the cage window (*i.e.*, within 5.5 Å of the centre of the cage core), but there was no diffusion into the cage cavity itself (Fig. 3c). On average, around 3% of the PCP molecules at any given time were located in a cage window, with around 16% being classed as near neighbours (*i.e.*, surrounding a cage). This high percentage of near neighbours is a result of the high cage solubility in PCP. These MD simulations support the hypothesis that the bulky PCP solvent is indeed unable to diffuse into the cage cores, thus maintaining the intrinsic cage cavities and forming a Type 2 porous liquid.

### Gas uptake

**Fig. 4:**
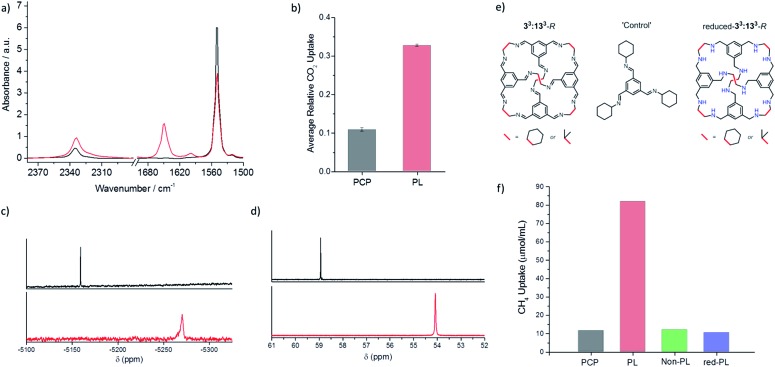
Comparison of CO_2_, CH_4_, Xe and SF_6_ uptakes in a 20% w/v porous liquid (PL, red spectra) *vs.* neat PCP solvent (black spectra) by FTIR and by NMR spectroscopy; all measurements taken at room temperature. (a) Liquid cell-FTIR spectra for the PL and PCP after saturation with CO_2_ showing the 1649 cm^–1^ band of the cage imine bond, the 1548 cm^–1^ band of the PCP alkene bond, and the CO_2_ adsorption band at 2335 cm^–1^; (b) average relative CO_2_ uptake calculated from the FTIR spectra using CO_2_ integrations 2300–2368 cm^–1^ relative to PCP integrations 1500–1579 cm^–1^ shows an apparent 3-fold increase in uptake in the PL; (c) stacked ^129^Xe NMR spectra showing Xe uptake in the PL and PCP; (d) stacked ^19^F NMR spectra showing SF_6_ uptake in the PL and PCP; (e) structures of the scrambled **3^3^:13^3^**-*R* cage mixture, the non-porous aromatic imine ‘control’ molecule, and the reduced-**3^3^:13^3^**-*R* cage mixture; (f) comparison of the calculated CH_4_ uptake (μmol mL^–1^) observed in PCP (black), the 20% w/v solution of the scrambled **3^3^:13^3^**-*R* cage mixture (porous liquid, red), the non-porous aromatic imine control molecule (green), and the reduced-**3^3^:13^3^**-*R* cage mixture (blue), all measured in PCP using ^1^H NMR analysis.

We next investigated gas solubility in this porous liquid. Initial testing focused on CO_2_ because of its distinctive infrared stretch, which allows rapid (qualitative) analysis using neat samples in a liquid FTIR cell. Both the porous liquid and neat PCP were analysed, both before and after saturation with CO_2_. By comparing the integration of the CO_2_ adsorption band at 2335 cm^–1^ with that of the PCP stretch at 1548 cm^–1^, we calculated an apparent 3-fold increase in the amount of CO_2_ taken up by the porous liquid relative to the solvent alone; a promising increase given the high baseline solubility of CO_2_ in ‘non porous’ chlorinated solvents^15^ (Fig. 4a and b).

Further FTIR investigations into the optimal CO_2_ loading conditions found that bubbling the gas through the porous liquid was sufficient to saturate the solution, with no increased uptake being observed when the solid cage was pre-exposed to CO_2_ prior to dissolution in PCP. Degassing the PCP prior to making the porous liquid, or sonication of the porous liquid itself before gas loading, had no effect on the CO_2_ uptake. Changing the flow rate of the gas, cooling the porous liquid, or adding one molar equivalent of water relative to cage had little effect on the quantity of the gas absorbed. The latter observation may be related to the poor solubility of water in PCP due to its hydrophobicity. Indeed, a reduction in CO_2_ uptake was only observed if the cage was ‘pre-wetted’ with water prior to dissolution in PCP: in this case, it seems that the water could act as a competitive guest in the cage cavity.^16^

Overall, the porous liquid's ability to absorb CO_2_ was robust and reproducible over multiple batches of scrambled cage and a variety of gas loading conditions (ESI Fig. 19–24†). Based on these data, it was decided that degassed PCP with a gas addition flow rate of 50–60 mL min^–1^ would be used for further studies. Five minutes of gas delivery per 1 mL of solvent was sufficient to saturate both the porous liquid and the neat PCP (ESI Fig. 25 and 26†).

We next focused on methane gas, which is, generally, more challenging to dissolve in liquid solvents than CO_2_. We previously showed that the CH_4_ uptake in the porous liquid could be monitored using ^1^H NMR analysis.^5^ To prevent deuterated solvent acting as a competitive guest, a sealed capillary containing d_2_-DCM and TMS was used to lock and reference the spectrum, respectively. This allowed ^1^H NMR analysis to be conducted on both the neat solvent and the porous liquid before and after CH_4_ addition. A shift in the methane signal was observed, from –0.24 ppm in pure PCP, to –2.80 ppm in the porous liquid.^5^ This strong shielding effect (Δ*δ* = –2.56 ppm) supports the presence of methane in the cage cavity on the NMR time scale.^17,18^ The presence of methane in the cage cavity is also apparent from a shift in all the cage signals (Δ*δ* = –0.06 ppm). A similar shielding effect was also observed for the gases xenon (Xe) and sulfur hexafluoride (SF_6_) within the cage cavities in this porous liquid, with respect to the neat PCP solvent, as analysed using ^129^Xe and ^19^F NMR (Fig. 4c and d). For Xe, a large shift and broadening of the signal was observed, from –5159 ppm in pure PCP to –5270 ppm in the porous liquid (Δ*δ* = –111 ppm). The same was true for SF_6_, with a peak shift (Δ*δ* = –4.86 ppm) from 58.94 ppm in pure PCP to 54.08 ppm in the porous liquid. This again supports the presence of these gases within the cage cavities in the porous liquid, with the degree of these shifts also being comparable to those reported for alternative host–guest systems studied for either Xe or SF_6_ encapsulation within a cryptophane in chlorinated solvents,^19^ or a metal–organic cage in water,^20^ respectively.

Calibration of the capillary with the porous liquid over a range of concentrations (25–200 mg_cage_ per mL_PCP_) made it possible to calculate a CH_4_ uptake of 51 μmol per g_PL_, as compared to just 6.7 μmol per g_PCP_ in neat PCP; that is, a 7.6-fold increase in gas solubility at 1 bar. This increase is comparable to the 8-fold relative increase observed in the crown ether porous liquid.^5^ The saturation CH_4_ solubilities are also similar for the two porous liquids (51 *vs.* 52 μmol per g_PL_). However, while the CH_4_ uptakes per gram of porous liquid are comparable, the amount of cage in a gram of liquid differs for the two systems. Indeed, the crown-ether porous liquid has more than double the molar cage concentration compared to the scrambled porous liquid (0.21 mmol per g_PL_ *vs.* 0.10 mmol per g_PL_). Hence, while the molar ratio of solvent to cage is higher in the scrambled system (36 : 1 *vs.* 12 : 1), it appears to be a more effective porous liquid in terms of methane adsorption, requiring just half as many cage cavities to achieve a similar gas uptake (see ESI Fig. 30†). This could be due to several factors, such as slight differences in the measurement temperature (20–25 °C for the scrambled porous liquid *vs.* 30 °C for the crown ether porous liquid), the different bulky solvents used (PCP *vs.* 15-crown-5), the cage substituents (methylpropane and cyclohexyl *vs.* large crown ethers), and the large difference in viscosity between the two porous liquids (11.7 cP *vs.* >140 cP)—all of these could affect the uptake kinetics and/or saturation solubility in the porous liquid. This comparison shows that saturation gas solubilities in porous liquids may not be a simple linear function of the number of cage cavities that are present, at least when comparing across different chemical systems.

### Control studies

To confirm that the increased gas uptakes are due to cage cavities, and not simply to some co-solvency effect caused by the hydrocarbon cage molecule, a non-porous aromatic imine ‘control molecule’ was prepared. This control molecule mimics a fragment of the cage (Fig. 4e), but it has no permanent cavity. Gas solubility tests for a 20% w/v solution of the control molecule in PCP were performed using the same methods to determine the solubility of CO_2_ (FTIR), CH_4_ (^1^H NMR), Xe (^129^Xe NMR), and SF_6_ (^19^F NMR). This control liquid showed no enhanced gas solubility over neat PCP (Fig. 4f and ESI Fig. 33†) and no NMR shielding effect for CH_4_, Xe, or SF_6_ (see ESI Fig. 34 and 36†).

This demonstrates that the increased solubility of these gases in the porous liquid is associated with the presence of empty pre-formed molecular-sized cavities. It is also important that the cavities are shape persistent: chemical reduction of the imines in the scrambled **3^3^:13^3^**-*R* cage mixture gave a reduced-**3^3^:13^3^**-*R* amine cage mixture (Fig. 4g) where we would expect the cages to be much more flexible.^21^ A 20% w/v solution of this material in PCP showed no enhanced gas solubility or shielding effect (Fig. 4f and ESI Fig. 35†). This suggests that the solubility enhancement arises from the permanent, preformed cavities: more flexible cages do not have the same solubilising effect, at least not at a gas pressure of 1 bar. We suggest that this is because the more flexible reduced cages do not exclude the PCP solvent, rather than these cages being necessarily ‘collapsed’ in solution.

### Shape- and size-selectivity

In addition to enhancing gas solubility, a porous liquid should, in principle, exhibit other properties associated with porous solids, such as guest selectivity. Previously, we showed that porous organic cages can exhibit shape- and size-selectivity and that this property was mirrored in the porous liquid.^5,22,23^ To evaluate the shape- and size-selectivity of the porous liquid, it was first saturated with xenon gas, which has a good geometric fit with the cage core.^23^ Addition to the xenon-saturated solution of a small antagonistic guest, in this case chloroform (one molar equivalent based on cage), led to rapid evolution of the gas,^5^ presumably because the chloroform can enter the cage core. By contrast, addition of one molar equivalent of a bulky guest, 1-*t*-butyl-3,5-dimethylbenzene did not lead to any gas evolution as the molecule is size-excluded from the cage core.^5^

### Gas release

**Fig. 5:**
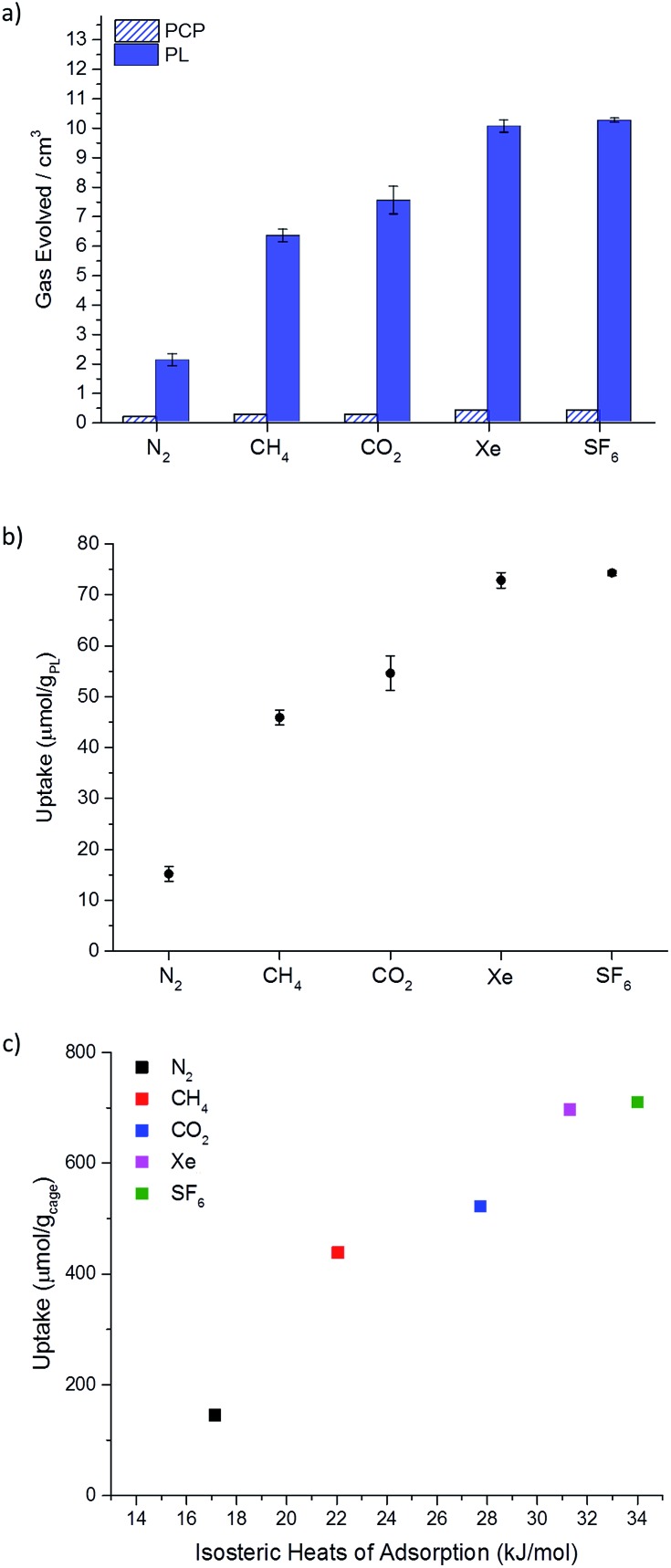
(a) Average gas evolution measured for N_2_, CH_4_, CO_2_, Xe, and SF_6_ (average of 3 independent measurements) both for the porous liquid and for PCP; standard deviations displayed as error bars. Calculated percentage occupancies for each gas in the porous liquid are also given, where a 1 : 1 guest-to-cage ratio would equate to 100%; (b) plot converting displaced gas volume (cm^3^) to estimated gas uptakes in terms of μmol per g_PL_; (c) apparent correlation between the uptake (μmol per g_cage_) for a range of different gases, calculated from the volumes of gas evolved from the PL (20% w/v), with the calculated isosteric heats of adsorption for the structurally related solid porous cage, **CC3**-*R*.^23,24^

This guest selectivity allowed us to carry out gas evolution measurements to obtain estimated gas uptakes within the porous liquid without resorting to techniques such as FTIR or NMR.^5^ This enabled us to study gases, such as nitrogen, that might otherwise have been challenging to measure. Chloroform was therefore added to samples of the porous liquid that had been saturated with N_2_, CH_4_, CO_2_, Xe, or SF_6_, using our previously optimised gas addition conditions (50–60 mL min^–1^ for 5 min per 1 mL PCP used). This was done in a closed system connected to a water-filled burette *via* cannula tubing (see ESI Fig. 37†), thus allowing the volume of released gas to be measured (Fig. 5a). Addition of one molar equivalent of chloroform relative to cage displaced a larger volume of gas from the porous liquid than when we added a large excess of chloroform, presumably because the displaced gas is somewhat solubilised in the excess chloroform (ESI Fig. 38†).

The percentage cage occupancy for each gas in the cages was estimated by comparing the average volume of displaced gas with the theoretical maximum, assuming a nominal 1 : 1 molar ratio of gas to cage.^5^ This estimate neglects any bulk solubility in the PCP solvent, but this is a reasonable approximation, particularly for the heavier gases studied. The porous liquid adsorbed all five gases, with the largest volumes of displaced gas observed for Xe (72.8 μmol per g_PL_) and SF_6_ (74.3 μmol per g_PL_). This equates to an average cage occupancy of 72% and 74% for Xe and SF_6_, respectively (Fig. 5a and b).

The volume of methane displaced matched the uptake determined by ^1^H NMR analysis (45.8 μmol per g_PL_ *vs.* 51 μmol per g_PL_); the 5.2 μmol g^–1^ difference between these values can be ascribed to the inherent solubility of CH_4_ in PCP (6.7 μmol g^–1^ by ^1^H NMR). Again, control experiments with neat gas-saturated PCP with the same five gases (Fig. 5a), and using CO_2_ with the non-porous aromatic imine control molecule (20% w/v in PCP; saturated with CO_2_, see ESI Fig. 39†) showed very little gas evolution (<0.3 cm^3^) upon addition of chloroform, again supporting the role of the cage cavities.

The calculated percentage cage occupancies (SF_6_ > Xe > CO_2_ > CH_4_ > N_2_) mirror the isosteric heats of adsorption that we calculated previously for the structurally related solid porous cage, **CC3**-*R*, (N_2_ = 17.14 kJ mol^–1^, CH_4_ = 22.05 kJ mol^–1^, CO_2_ = 27.73 kJ mol^–1^, Xe = 31.31 kJ mol^–1^ and SF_6_ = 36.90 kJ mol^–1^).^23,24^ In the solid state, adsorption can occur both in the intrinsic cage cavities and in extrinsic cavities between cages, whereas in the porous liquid, the cages cavities are likely to dominate. Despite this difference, there appears to be a good correlation between the computed isosteric heats for the **CC3**-*R* cage solid and the measured gas uptakes in the analogous porous liquid (Fig. 5c), suggesting that physical principles learned from studies on porous organic solids may be transferred to these liquid materials.

**Fig. 6:**
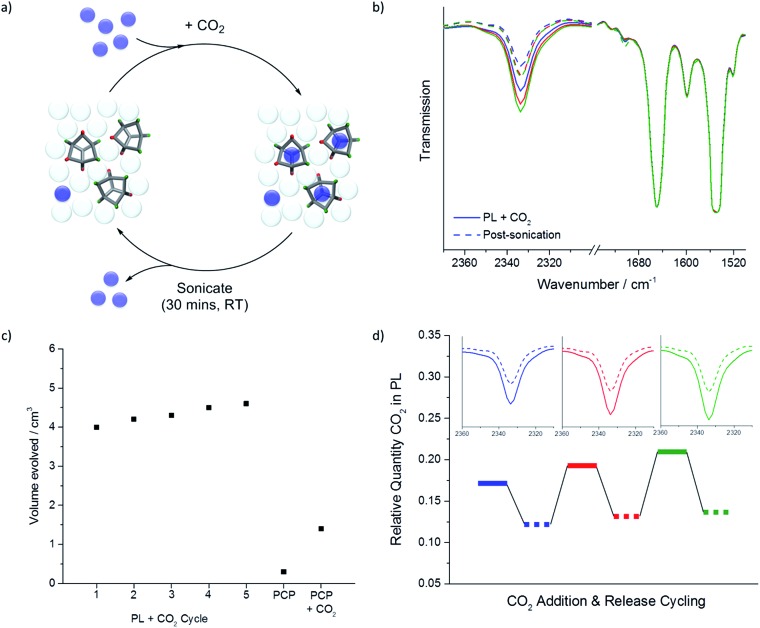
(a) Scheme demonstrating a potential gas loading/release cycle using sonication as a trigger; (b) overlaid liquid cell-FTIR spectra for the porous liquid over three CO_2_ loading/release cycles (1^st^ cycle blue, 2^nd^ red, 3^rd^ green; solid lines = saturation; dashed lines = post sonication); (c) measured volumes of CO_2_ evolved using sonication as the release mechanism for both the PL over five successive cycles and PCP; (d) relative quantity of CO_2_ within the porous liquid over three loading/sonication cycles calculated from the FTIR spectra, with insets showing the FTIR CO_2_ stretch in the porous liquid pre- and post-sonication.

Clearly, guest release from a porous liquid by addition of an antagonistic guest, such as chloroform, is an unattractive scheme for practical applications, not least because it is irreversible, unless the antagonistic guest is removed again. Alternative schemes could be pressure or temperature swings, but here we investigated sonication as a non-chemical method for gas release from porous liquids. While this did not prove to be as efficient as the addition of chloroform (up to 4.6 cm^3^ *vs.* 7.5 cm^3^ CO_2_ evolved), it allowed us to investigate gas loading/release cycles for the porous liquid without the obvious disadvantage of having to add and then remove an antagonistic guest (Fig. 6). The volume of CO_2_ released by sonication remained relatively constant over at least 5 gas loading-release cycles (Fig. 6c). The slight increase in CO_2_ release with cycling could be a result of successive CO_2_ sweeps removing small competitive impurity guests, such as water and/or nitrogen. In comparison with an equivalent sonication loading-release cycle for a CO_2_-saturated sample of neat PCP, this represents a 3-fold increase in the amount of CO_2_ that can be adsorbed and then released in the porous liquid. Again, this increase is encouraging given that CO_2_ has relatively good solubility in chlorinated solvents in any case.^15^ One might expect more pronounced gains for gases that have lower native solubility in common organic solvents.

The ability to mechanically displace gases from porous liquids might allow the development of new practical technologies for gas capture and gas separation. For applications involving flow transport, viscosity will be a key parameter. The viscosities of PCP, the non-porous imine control molecule (20% w/v in PCP), and the porous liquid (20% w/v in PCP) were measured at 22 °C and found to be 3.3, 6.5, and 11.7 cP, respectively. The presence of 20% w/v cage in PCP therefore increases the viscosity ∼3.5 fold; the non-porous aromatic imine control molecule also doubled the viscosity, but without a corresponding increase in gas uptake. For comparison, the viscosity of the Selexol® solvent that is used commercially to remove acid gases at high pressures (a mixture of dimethyl ethers of polyethylene glycol) is 5.8 cP at 298 K.^25^

### Chiral selectivity

In addition to shape- and size-selectivity, we also showed previously that solid porous cages can exhibit chiral selectivity, and such cages can be used to separate chiral enantiomers. For example, homochiral **CC3**-*R* preferentially absorbs the (*S*)-enantiomer of 1-phenylethanol, with an enantiomeric excess (ee) of up to 30%.^23,26^ One component of the scrambled **3^3^:13^3^**-*R* cage, (*R*,*R*)-1,2-diaminocyclohexane, is homochiral; this is the same diamine used to produce **CC3**-*R*. The solid scrambled **3^3^:13^3^**-*R* mixture and the resulting porous liquid were therefore investigated to see whether chiral selectivity is observed. The solid scrambled **3^3^:13^3^**-*R* mixture gave an ee of approximately 14% when exposed to one equivalent of rac-1-phenylethanol; this matches previous results for **CC3**-*R*, which showed an ee of approximately 15% for the equivalent ratio of the same racemic guest.^23^

However, when the same protocol was used with the chiral porous liquid, no ee was measured (ESI Fig. 40†). Even increasing the guest : host ratio to 2 : 1, which for **CC3**-*R* increases the ee to 30%, gave an ee of zero in the porous liquid. The chiral selectivity in **CC3**-*R* is thought to arise from favourable interactions between the hydroxyl group in the alcohol and the hydrogen and nitrogen atoms of the imine in **CC3**-*R*. This, along with π–π interactions between aryl groups in the cage and the alcohol, are far more apparent for (*S*)-1-phenylethanol and **CC3**-*R* than for its (*R*)-enantiomer. Previous simulations^23^ showed that these interactions are maximised when the phenyl group of 1-phenylethanol is located in the centre of a cage while the chiral centre of the alcohol occupies a cage window and interacts with a neighbouring cage. The lack of any neighbouring cages in the porous liquid (see model, Fig. 3b) could explain why no chiral selectivity occurs.

### Comparison to analogous solid porous organic cages

**Fig. 7:**
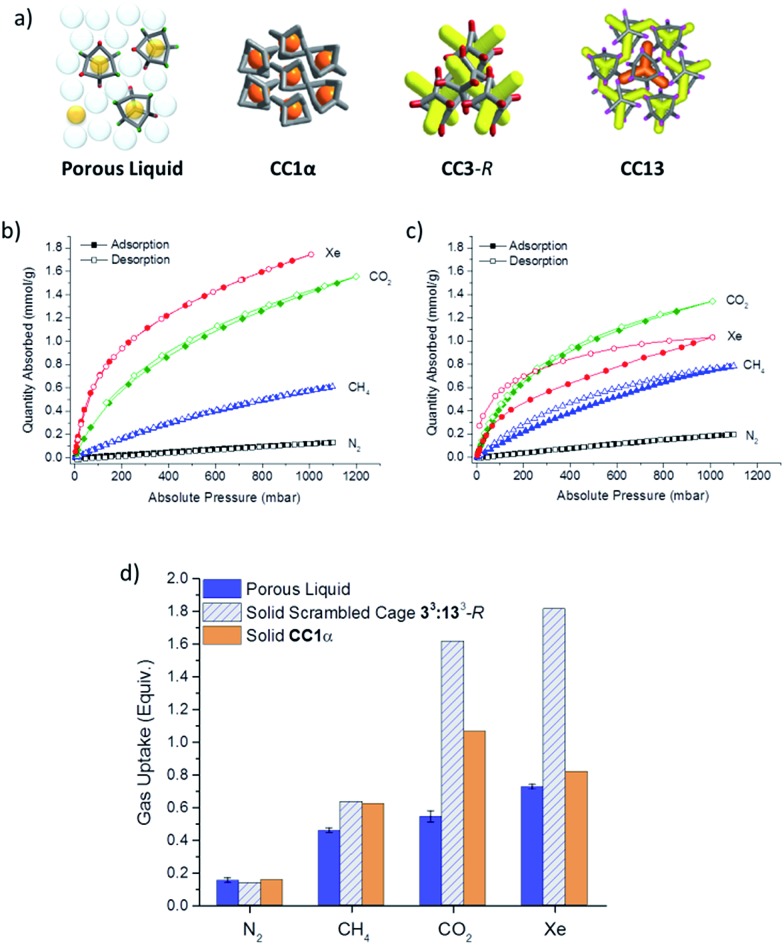
(a) Schematic structures of the porous liquid and analogous porous solids. **CC1α** packs in a window-to-arene fashion and contains only isolated voids within the cage itself (grey = core cage structure; orange = isolated cage voids). Solid, scrambled **3^3^:13^3^**-*R* (not illustrated) will have the same cage cavities, but will also have some degree of extrinsic porosity between cages. Crystalline **CC3**-*R* and **CC13** have interconnected pore networks (yellow); crystalline **CC13** also has some formally isolated cage voids (orange); grey = core cage structure; red = cyclohexane vertices; pink = methylpropane vertices; (b) gas sorption isotherms for solid scrambled **3^3^:13^3^**-*R* cages at 293 K; (c) gas sorption isotherms for solid **CC1α** at 293 K, shown on the same scale; the material can adsorb gases at this temperature despite its lack of formally connected pore channels; (d) comparison of the molecular equivalents of gas per cage for the porous liquid, calculated from the amount of gas evolved, and for both the solid scrambled **3^3^:13^3^**-*R* cage and **CC1α**, as determined by gas sorption measurements.

We next set out to understand how the gas uptake of cages in the porous liquid relates to gas adsorption for the scrambled cages and other analogous cages in the solid state. To do this, the quantity of gas displaced from the porous liquid was compared with adsorption isotherms for both the solid scrambled **3^3^:13^3^**-*R* cage mixture and also pure **CC1α**,^12^ both at 293 K (Fig. 7). **CC1α** was chosen because its closely packed window-to-arene structure contains intrinsic cage voids only (Fig. 7a), and no extrinsic porosity.^12^ This absence of extrinsic voids between cages in **CC1α**, along with its very similar cavity size, makes it a good solid state comparison with the porous liquid, which also lacks any appreciable extrinsic porosity according to our molecular models.

The solid scrambled **3^3^:13^3^**-*R* cage mixture was microporous with an apparent Brunauer–Emmett–Teller surface area (SA_BET_) of 519–629 m^2^ g^–1^. By comparison with other amorphous systems, for which we have built structural models,^27^ this gas uptake is probably a result of both adsorption within the cage cavities (intrinsic pores) and also adsorption in voids between neighbouring cages (extrinsic pores). As such, comparison with the uptake of the scrambled cage in the solid state allows us to assess how much extrinsic porosity in the solid **3^3^:13^3^**-*R* cage mixture is ‘lost’ by forming the porous liquid.

There is a fair correlation between the volume of gas that is displaced from the porous liquid and the gas sorption measurements for solid **CC1α**, as expressed on a ‘per cage’ basis, for N_2_, CH_4_, CO_2_, and Xe (Fig. 7d). However, for the solid, scrambled **3^3^:13^3^**-*R* cage, the CO_2_ and Xe uptakes per cage were markedly higher than observed for the porous liquid (Fig. 7d). We ascribe this to guest accessible extrinsic pores between cages in the solid state for scrambled **3^3^:13^3^**-*R*, which is not present in the liquid.

### Gas retention

We next investigated the gas retention of the porous liquid over time. The scrambled cage was shown to be chemically stable in PCP over a period of 30 days, as determined by HPLC and ^1^H NMR analysis (ESI Fig. 17†). However, on monitoring both CO_2_ (FTIR) and CH_4_ (^1^H NMR) saturated samples of porous liquid (1 mL) left in capped vials (4 mL), under atmospheric conditions and at ambient temperature, the liquid appeared to only retain CO_2_ and CH_4_ for 3 days and 7 days, respectively (ESI Fig. 27 and 32†). We hypothesise that this loss of gas from the saturated porous liquid solution occurs because gas molecules exchange between the cage cavity and the bulk solvent, with slow release occurring when a gas molecule does not re-enter a cage molecule but instead is released at the liquid–air interface. It is also possible, of course, that cages approach the liquid–air interface whereby gas molecules could be lost directly.

### Diffusion NMR – cage aggregation and host–guest chemistry

A single methane signal was observed in ^1^H NMR gas uptake studies in the porous liquid, (ESI Fig. 34†); likewise, a single signal was also observed in the ^129^Xe and ^19^F NMR spectra for Xe and SF_6_, respectively (Fig. 4c and d). These single, shifted peaks could be interpreted as an average signal for bound and unbound guests within the system. This would occur if the gas binding is in dynamic equilibrium and exchange is much faster than the NMR timescale. With slow exchange (or no exchange) then two resonances might be expected; one for the free gas in the PCP solvent and one for the bound gas in the cage cavity. Investigations using low temperature NMR, in an attempt to resolve separate signals for the bound and unbound CH_4_ in the porous liquid, proved unsuccessful, with precipitation of the cage occurring before peak resolution could be obtained. We therefore used diffusion NMR spectroscopy to better understand the host–guest interactions. Previous reports demonstrate that diffusion NMR can be used to determine the size of individual species in solution^28^ and to assess the magnitude of association constants in host–guest systems.^29^ Where a guest is bound strongly, and exchange is slow, the guest diffusion coefficient will closely match that of the host.^30^ This results from the host–guest species diffusing as a single supramolecular entity. When exchange is fast, diffusion NMR can be used to estimate association constants (*K*_a_) and to determine the proportion of time that the guest remains bound.^31^

First, the behaviour of the guest-free scrambled cages in the porous liquid was investigated to determine whether any aggregation of the cages was occurring, particularly since we are close to the saturation solubility in PCP (see above). In principle, gas uptake might be affected by cage aggregates, for example through the formation of cage–cage dimers with extrinsic intercage cavities. By measuring the diffusion coefficient of the cages in the porous liquid over a range of concentrations (2.5 to 20% w/v), along with the viscosity of each solution, it was possible to calculate the apparent solvodynamic radii (*R*_S_, nm) of the cage species.

**Fig. 8:**
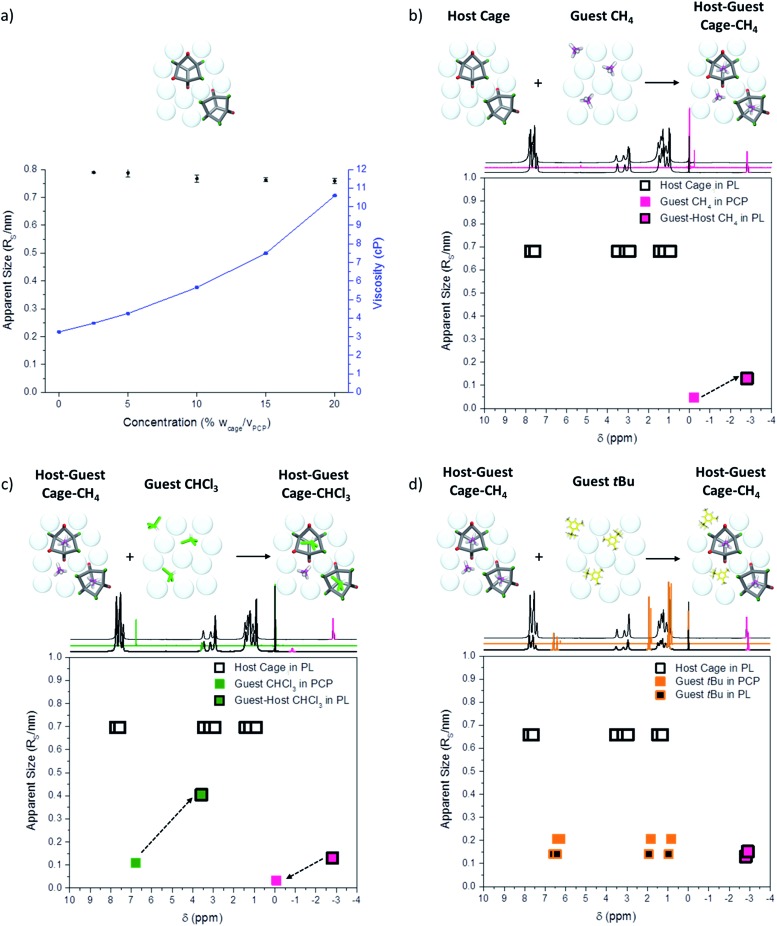
Study of host–guest chemistry in the porous liquid by diffusion NMR. (a) Measured viscosities and calculated apparent solvodynamic radii (*R*_S_) of scrambled cages in the porous liquid at different concentrations. The viscosity increases with concentration but the apparent size of cage remains constant, suggesting that no aggregation in solution is occurring; (b) diffusion NMR measurements for CH_4_ in PCP and the porous liquid show an ∼3-fold increase in the apparent size (*R*_S_) of the gas and a strong shielding effect, both suggesting a dynamic host–guest equilibrium between the cage and CH_4_ (empty host cage = black squares; free CH_4_ guest in neat PCP = pink square; pink-filled black square = CH_4_/cage host–guest complex) (c) addition of CHCl_3_ to the CH_4_-saturated porous liquid shows displacement of the CH_4_, with a subsequent decrease in apparent size and reduced shielding effect for CH_4_; at the same time, a ∼4 fold increase in the apparent size (*R*_S_) of the CHCl_3_ is observed, along with a strong shielding effect, again suggesting a dynamic host–guest equilibrium between the cage and CHCl_3_ (free CHCl_3_ guest in neat PCP = green square, green-filled black square = CHCl_3_/cage host–guest complex); (d) addition of 1-*t*-butyl-3,5-dimethylbenzene (*t*Bu, orange squares) to the CH_4_-saturated porous liquid shows no displacement of CH_4_, and no apparent increase in size (*R*_S_) or shielding effect of the bulky *t*Bu-solvent, confirming that it is size-excluded from the cage cavity.

While the viscosity of the porous liquid solutions did increase with increasing cage concentration, the apparent size of the cage remained essentially constant, with a solvodynamic radius of around of around 0.75–0.8 nm (Fig. 8a). This size is consistent with dimensions derived from single crystal X-ray diffraction for a single [4 + 6] molecular cage. Therefore, diffusion NMR indicates that the scrambled cages in the porous liquid exist as discrete molecular species over this concentration range, and that they are not aggregated on the NMR timescale. It is thus likely that gas uptake in the porous liquid is solely due to the isolated cage cavities with no additional cooperative extrinsic cavities, in keeping with our MD simulations (Fig. 3b).

To investigate the host–guest chemistry, the relative size of each guest (CH_4_, CHCl_3_, and 1-*t*-butyl-3,5-dimethylbenzene) was compared in the porous liquid (20% w/v scrambled cage in PCP), the neat solvent (PCP), and also the non-porous imine control liquid (20% w/v in PCP). As expected, the apparent sizes of the guest molecules in PCP and in the non-porous imine control liquid, were essentially the same due to the absence of any appreciable host–guest interactions (ESI Fig. 43†). However, when the porous liquid was saturated with CH_4_, there was a reproducible increase in the apparent CH_4_ solvodynamic radius (*R*_S_) from 0.047 nm in PCP to 0.13 nm in the porous liquid. This is substantially smaller than the apparent size of cage molecule (1.5 nm, Fig. 8b), and we ascribe this increase to fast exchange between gas that is ‘bound’ in the cage cavity as a host–guest complex and gas that is ‘unbound’ in the PCP. Upon addition of a small volume of CHCl_3_ to this CH_4_-saturated porous liquid (0.85 molar equivalents relative to cage), the CH_4_ is displaced, as discussed earlier (Fig. 5), with a dramatic peak shift from –2.81 ppm to –0.08 ppm, and a reduction in the apparent CH_4_ size, suggesting that it is no longer bound in the cage cavity (Fig. 8c). Accompanying this, the apparent size (*R*_S_) of CHCl_3_ increases from 0.11 nm in PCP to 0.40 nm in the porous liquid. This represents a greater apparent size increase than observed for CH_4_, but it is again much smaller than the apparent size of the cage, suggesting that the CHCl_3_ guest is more strongly bound than CH_4_ but also in dynamic equilibrium. A strong shielding effect was observed for the CHCl_3_, from 6.79 ppm in pure PCP to 3.58 ppm in the porous liquid (Δ*δ* = –3.21 ppm), which provides further support of the presence of the CHCl_3_ within the cage cavity on the NMR timescale. Finally, addition of 1-*t*-butyl-3,5-dimethylbenzene to CH_4_-saturated porous liquid caused no loss of CH_4_ and no increase in its apparent size, confirming that this molecule is size-excluded from the cage cavity (Fig. 8d).

Interestingly, there was a slight increase in the apparent size (*R*_S_) of CH_4_ upon addition of 1-*t*-butyl-3,5-dimethylbenzene (0.13 nm to 0.15 nm) and a further shift in the ^1^H NMR suggesting the CH_4_ is experiencing an increased shielding effect (–2.81 ppm to –2.91 ppm). This could open up interesting avenues into studying the effect of using different size-excluded solvents, or mixed systems, on the dynamic equilibrium of gas uptake, possibly changing the preference of the gas to be located in either the solvent or the cage cavity.

Taken as a whole, the diffusion NMR data suggest that the guests CH_4_ and CHCl_3_ are bound in the cage for ∼68% and ∼86% of the time, respectively (see ESI Section 8.2.3†).^32^ The higher binding affinity of CHCl_3_ is consistent with our gas evolution studies where this molecule was used to displace CH_4_. This supports our theory that gas release from the porous liquid is a result of the dynamic equilibrium between being bound in the cage cavity and unbound in the solvent, with gas loss occurring when the gas does not re-enter a cage and is instead released at the surface of the liquid. The dynamic nature of the host–guest interaction between the cage and gas is also in agreement with the recent conclusions of Qiao *et al.*, who found through molecular simulations that different gas molecules in a crown-ether cage porous liquid could rapidly exchange between the cage and the solvent.^9^

## Conclusions

By using various measurement techniques, combined with molecular simulations and control experiments, we have significantly advanced the quantitative understanding of these Type 2 porous liquids compared to our initial study.^5^ We show that it is possible to increase the solubility of porous organic cages by using a dynamic covalent scrambling strategy. A scrambled combination was discovered, **3^3^:13^3^**-*R*, which is more than 10 wt% soluble in PCP. This system is not unique: although not investigated in detail here, other scrambled systems showed comparable solubility in more than one bulky solvent (Fig. 1b), and it should be possible for other research groups to design ‘task specific’ porous liquids using this scrambling strategy, most likely with solubilities (and hence porosity levels) that exceed those reported here.

MD simulations support our inference that PCP is size-excluded from the cages, confirming that a Type 2 porous liquid was formed. After optimisation of gas addition methods using *in situ* FTIR, this liquid demonstrated enhanced gas uptake and guest selectivity. For example, the solubilities of Xe and SF_6_ in the porous liquid (Fig. 5a) are estimated to be around 22 times higher than for the neat PCP solvent. We also show unequivocally that a shape persistent cavity is required: neither flexible reduced amine cages nor non-porous imine control molecules give rise to this gas solubility enhancement. Diffusion NMR experiments show no evidence of cage aggregation in solution, suggesting that our model (Fig. 3b) of isolated cage cavities is an appropriate description for these systems.

The cage occupancies were measured for gas-loaded liquids, both volumetrically and by *in situ* ^1^H NMR spectroscopy, and were found to be as high as 72% and 74% for Xe and SF_6_, respectively. Comparison with gas adsorption measurements for organic cages in their solid form demonstrate that porous liquids have a gas affinity that is similar to related porous organic solids, such as **CC1α**, where there is little or no extrinsic porosity. Importantly, it was also shown for the first time that gases can be reversibly loaded and unloaded in porous liquids without using an antagonistic guest, for example by using sonication (Fig. 6). It is possible that similar schemes could be devised whereby gases are loaded and unloaded using thermal or pressure cycling, as for porous solids.

As outlined above, porous liquids behave somewhat like porous organic molecular solids in many respects but there are also differences: for example, solid scrambled **3^3^:13^3^**-*R* cage mixtures exhibit chiral selectivity while the porous liquid does not, probably because the chiral binding event requires the presence of two adjacent cages.^23^ Also, diffusion NMR suggests that the gas in these liquids is in dynamic equilibrium between bound and unbound states within the cage cavity, in agreement with recent simulations for related porous liquids.^9^ This rationalises the slow loss of gas from these porous liquids over the period of a few days.

Taken together, these findings should help to establish porous liquids as a new platform for materials research and to provide guidelines for other teams seeking to design new systems. Challenges for the future include reducing the cost and improving sustainability; for example, by discovering porous liquids where the solvent and/or the cage are derived from renewable feedstocks. It might also be practically useful to produce porous liquids that have zero or near-zero volatility: for example, to allow pressure, or temperature, swing schemes. While the initial cage–solvent combinations that we investigated showed insufficient solubility (Fig. 1b), the development of ‘porous ionic liquids’ remains an attractive target for the future.^33,34^
