# Supplementary material for: Understanding gas capacity, guest selectivity, and diffusion in porous liquids
Source: Chem Sci. 2017 Jan 31;8(4):2640–51. doi: 10.1039/c6sc05196k (PMC5431659; doi:10.1039/c6sc05196k)
Supplement: Supplementary file 1 [file SC-008-C6SC05196K-s001.pdf]

Electronic Supplementary Material (ESI) for Chemical Science.
This journal is © The Royal Society of Chemistry 2017

Supporting Information

Understanding Gas Capacity, Guest Selectivity and Diffusion in Porous Liquids

Rebecca L. Greenaway,† Daniel Holden,† Edward G. B. Eden,† Andrew Stephenson,† Chin W. Yong,‡ Michael J. Bennison,† Tom Hasell,† Michael E. Briggs,† Stuart L. James§ and Andrew I. Cooper*,†

† Department of Chemistry and Materials Innovation Factory, University of Liverpool, Crown Street, Liverpool, L69 7ZD, United Kingdom

‡ Scientific Computing Department, Science and Technologies Facilities Council, Daresbury Laboratory, Sci-Tech Daresbury, Warrington, WA4 4AD, United Kingdom; Manchester Pharmacy School, Faculty of Medical and Human Sciences, Manchester Academic Health Science Centre, University of Manchester, Manchester, M13 9NT, United Kingdom.

§ School of Chemistry and Chemical Engineering, Queen's University Belfast, David Keir Building, Stranmillis Road, Belfast, BT9 5AG, United Kingdom

This file includes:

1. General synthetic and analytical methods
2. Screening for a porous liquid
3. Synthesis, purification, and characterization
4. Computational model and molecular dynamics simulations
5. Spectroscopic gas uptake and release studies
6. Guest selectivity
7. Porosity in porous liquids vs porous organic cages
8. Diffusion NMR and host-guest chemistry

The following abbreviations have been used throughout the supporting information:

PCP	Hexachloropropene
PL	Porous liquid
Non-PL	Control Non-porous liquid
SIC	Small imine control
Red-PL	Reduced scrambled liquid

1. General Synthetic and Analytical Methods

Materials: 1,3,5-Triformylbenzene was purchased from Manchester Organics, UK, and used as received. Other chemicals were purchased from Sigma-Aldrich or TCI UK and used as received, unless otherwise stated. PCP was purchased from Sigma-Aldrich. Other solvents were reagent or HPLC grade purchased from Fisher Scientific.

Methods: All reactions requiring anhydrous or inert conditions were performed in oven dried apparatus under an inert atmosphere of dry nitrogen, using dried and purified solvents (PCP and 1-*t*-butyl-3,5-dimethylbenzene – see **section 3.3**) introduced using disposable needles and syringes. All reactions were stirred magnetically using Teflon-coated stirring bars. Removal of solvents was done using a rotary evaporator.

TLC: Reactions were monitored by thin layer chromatography (TLC), conducted on pre-coated aluminium-backed plates (Merck Kieselgel 60 with fluorescent indicator UV254). Spots were visualized by quenching of UV fluorescence.

HPLC: HPLC was conducted on a Dionex UltiMate 3000 equipped with a diode array UV detector using a Thermo-Scientific Syncronis C8 column, 150x4.6 mm, 3 µm (SN 10136940, Lot 12459). The mobile phase was isocratic MeOH at a flow rate of 1 mL/min for a 10 min run time. The injection volume was 10 µL and the sample concentration was approximately 1 mg/mL. Detection for UV analysis was conducted at 254 nm.

Melting Points: Obtained using Griffin melting point apparatus and are uncorrected.

FTIR: Infra-red spectra were recorded on a Bruker Tensor 27 FT-IR using ATR measurements for solid samples, and single sample transmissions for neat liquids with a Specac omni-cell demountable liquid cell with calcium fluoride (CaF_2) plates and a 0.05 mm PTFE insert (Scans: 16 background, 32 sample).

NMR: ^1H Nuclear magnetic resonance spectra were recorded using an internal deuterium lock for the residual protons in $CDCl_3$ (δ = 7.26 ppm) or d_2-DCM (δ = 5.32 ppm) at ambient probe temperature on the following instruments: Bruker Avance 400 (400 MHz) or Bruker DRX500 (500 MHz). NMR studies were conducted using in-house made calibrated capillary of TMS in d_2-DCM (100 µL sample from 10 µL TMS in 0.5 mL d_2-DCM), so as to have no effect on the studies of the PL.

Data are presented as follows: chemical shift, integration, peak multiplicity (s = singlet, d = doublet, t = triplet, q = quartet, qu = quintet, sex = sextet, sept = septet, m = multiplet, br = broad, app = apparent), coupling constants (*J* / Hz) and assignment. Chemical shifts are expressed in ppm on a δ scale relative to δ_{TMS} (0 ppm) or δ_{CDCl3} (7.26 ppm) Assignments were determined either on the basis of unambiguous chemical shift or coupling patterns or by analogy to fully interpreted spectra for structurally related compounds.

^{13}C NMR Spectra were recorded using an internal deuterium lock using $CDCl_3$ (δ = 77.16 ppm) or d_2-DCM (δ = 54 ppm) at ambient probe temperatures on the following instruments: Bruker Avance 400 (101 MHz) or Bruker DRX500 (126 MHz).

^{19}F NMR Spectra were recorded using an internal deuterium lock using a sealed capillary of TMS in $CDCl_3$ at a nominal probe temperature of 298 K on the following instrument: Bruker Avance 400 (376 MHz).

^{129}Xe NMR Spectra were recorded using an internal deuterium lock using a sealed capillary of TMS in $CDCl_3$ at a nominal probe temperature of 298 K on the following instrument: Bruker Avance II (400 MHz – ^{1}H) wide bore spectrometer operating at 110.64 MHz.

HRMS: High resolution mass spectrometry was carried out using an Agilent Technologies 6530B accurate-mass QTOF Dual ESI mass spectrometer (capillary voltage 4000 V, fragmentor 225 V) in positive-ion detection mode. The mobile phase was MeOH + 0.1% formic acid at a flow rate of 0.25 mL/min.

TGA: Thermogravimetric analysis was carried out using a Q5000IR analyser (TA instruments) with an automated vertical overhead thermobalance. The samples were heated at the rate of 5 °C/min to 600 °C in an aluminium pan under a nitrogen flow. All materials were desolvated by heating to 90 °C in a vacuum oven overnight prior to TGA analysis.

SEM: Scanning electron microscopy was conducted using a Hitachi S4800 scanning electron microscope. Powdered samples of scrambled cage were deposited onto adhesive graphite tabs mounted on 15 mm aluminium stages.

PXRD: Laboratory powder X-ray diffraction data were collected in transmission mode on samples held on thin Mylar film in aluminium well plates on a Panalytical X'Pert PRO MPD equipped with a high throughput screening (HTS) XYZ stage, X-ray focusing mirror and PIXcel detector, using Ni-filtered Cu Kα radiation. Data were measured over the range 4–50° in ~0.013° steps over 60 minutes.

Gas Sorption Analysis: Surface areas were measured by nitrogen sorption at 77.3 K. Powder samples were degassed offline at 90 °C for 15 h under dynamic vacuum (10^{-5} bar) before analysis, followed by degassing on the analysis port under vacuum, also at 90 °C. Isotherms were measured using Micromeritics 2020, or 2420 volumetric adsorption analyzer. Gas uptake measurements (for N_2, CH_4, CO_2, and Xe) were taken at a temperature of 293 K, stabilized using a circulating water chiller/heater.

Gas Uptake and Evolution Studies: All samples of the PL were tested with gases purchased from BOC of the following grades: carbon dioxide (N5.0), methane (N4.5), xenon (N5.0) and sulphur hexafluoride (N3.0) in GC headspace vials (22 mm x 45 mm screw top, 10 mL, Fisher Scientific).

All samples had gas addition and measurements conducted between 17 and 25 °C in a temperature-controlled laboratory.

The flow rate of gas addition was measured using a Gilmont calibrated/correlated flowmeter (tube size 0, Gilmont EW-03201-22) with a stainless steel float on a scale of 1-100. The flow of each gas was calculated from the supplied correlated flow table for air, and general correction equations for approximating gas flow from air using the density of each gas in g/mL at standard conditions taken from the NIST Chemistry WebBook[1] (Gilmont calibrated at 1 atm, 294 K), with corrections for temperature and pressure. After optimisation, each gas was maintained at ~50-60 mL/min flow rate by setting the regulator output pressure to 0.5 bar and fine-controlling the flow with a needle valve to the calculated scale readings (see table).

Gas evolution was measured by displacement of water in an inverted Rotaflo stopcock 10 or 25 mL burette (0.1 mL graduations) in a beaker of water connected to the GC vial containing the sample *via* a needle/tubing cannula. Measurements were repeated a total of three times—two times on one batch and another on a different batch of cage material, at the same temperature to obtain a mean gas evolution and corresponding standard deviation.

General Correction Equations for Approximating Gas Flow from Air Flow Readings:

Gas Flow from Air Flow:

$$q_G^{\circ} = q_A^{\circ}\sqrt{\frac{\rho_{Air}^{\circ}}{\rho_G^{\circ}}}$$

Corrections for Temperature and Pressure:

$$q_G' = q_G^{\circ}\sqrt{\frac{p}{p^{\circ}}\cdot\frac{T^{\circ}}{T}}$$

$$\boldsymbol{Standard\ Conditions}: \boldsymbol{p}^{\circ} = \mathbf{1}\ \boldsymbol{atm}, \boldsymbol{T}^{\circ} = \mathbf{70}\ ^{\circ}\mathrm{F}/\mathbf{21}\ ^{\circ}\mathrm{C}/\mathbf{294}\ \boldsymbol{K}$$

$$q_A^{\circ} = standard\ air\ flow\ reading\ from\ meter\ (mL/\mathrm{min})$$

$$q_G^{\circ} = standard\ gas\ flow\ in\ same\ units\ (mL/\mathrm{min})$$

$$\rho_{Air}^{\circ} = density\ of\ air\ at\ standard\ conditions\ (g/\mathrm{mL})$$

$$\rho_G^{\circ} = density\ of\ gas\ at\ standard\ conditions\ (g/\mathrm{mL})$$

$$q_G' = gas\ flow\ at\ P\ and\ T\ with\ volume\ corrected\ to\ measurement\ at\ standard\ conditions\ (mL/\mathrm{min})$$

$$p = absolute\ pressure\ of\ gas\ inlet\ (atm)$$

$$T = absolute\ temperature$$

Calculating gas flow from air flow and corresponding Gilmont Flowmeter Scale Reading:

Gas	Density of gas (g/mL) at standard conditions (1 atm, 70 °F/21 °C/294 K) from NIST WebBook	Gilmont Scale Reading (SS Float)	Calibrated Air Flow q_A° at standard conditions (mL/min)	Corrected Gas Flow q_G° from Air Flow q_A° at standard conditions (mL/min)
**Air**	$\rho_{Air}^{\circ} = 0.00120$	39–43	49.69–58.40	N/A
**N_2**	$\rho_G^{\circ} = 0.00116$	39–43	49.69–58.40	50.53–59.39
**CO_2**	$\rho_G^{\circ} = 0.00183$	44–49	60.74–73.42	49.18–59.45
**CH_4**	$\rho_G^{\circ} = 0.00066$	32–36	36.50–43.76	49.21–59.00
**Xe**	$\rho_G^{\circ} = 0.00546$	60–66	105.2–124.7	49.31–58.46
**SF_6**	$\rho_G^{\circ} = 0.006127$	62–69	111.5–135.0	49.34–59.74

GC: Gas chromatography measurements were carried out using a Thermo Scientific TRACE 1310 instrument configured with an FID detector and a 2,3-di-*O*-methyl-6-*O*-TBDMS-*β*-cyclodextrin capillary column (Supelco Beta DEX 325; 30 m × 0.25 mm × 0.25 μm). Samples were analysed using headspace injections and were performed by incubating the sample at 100 °C for 30 minutes followed by sampling 1 mL of the samples headspace. The following GC method was used; the oven was programmed from 95 °C with 35 min hold and 10 °C/min increments to 150 °C with 4 min hold, the total run time was 44.5 min; injection temperature 300 °C; detection temperature 300 °C with hydrogen, air, and make-up flow-rates of 35, 350, and 35 mL/min respectively; helium (carrier gas) flow-rate 1 mL/min. The samples were injected in the split mode (5:1). Numeric integration of the resulting peaks was performed using the supplied Chromeleon 7.1.2.1478 (Thermo Scientific Corporation) software package.

Diffusion NMR: All measurements were carried out non-spinning on a 400 MHz Bruker Avance 400 spectrometer, using a 5 mm indirect detection probe, equipped with a z-gradient coil producing a nominal maximum gradient of 34 G/cm. Diffusion data was collected using the Bruker longitudinal eddy current delay (LED) pulse sequence (ledgp2s). In the case of highly concentrated, viscous samples, bipolar gradients were used (ledbpgp2s) to minimise artefacts in the spectrum. A diffusion encoding pulse δ of length 1–7 ms, and diffusion delay D of 0.1–0.25 s were used. Gradient amplitudes were equally spaced between 1.70 and 32.4 G/cm. Each FID was acquired using 16 k data points. All experiments were carried out at a nominal probe temperature of 298 K, with an air flow of 800 m^3/min to minimise convection. A sealed lock tube containing TMS in d_2-DCM (100 μL sample from 10 μL TMS in 0.5 mL d_2-DCM) was used to facilitate a deuterium lock without affecting the chemical makeup of the PL. All measurements were carried out three times and the numbers quoted represent the mean.

Diffusion coefficients were calculated from signal intensities using the Skejskal-Tanner equation[2]:

$$I = I_0 e^{\gamma^2 g^2 \delta^2 \left(\Delta - \delta/3\right) D}$$

Where I is the signal intensity, I_0 is the signal intensity at a gradient strength of zero, g is the gradient strength, and D is the diffusion coefficient (D = m^2/s). Solvodynamic radii, R_S (nm), of solution-phase species were calculated from the Stokes-Einstein equation assuming molecules have a spherical geometry:

$$D = \frac{kT}{6\pi\eta R_S}$$

Where kT is the thermal energy (k = 1.38 x 10^{-23} N m/K; T = 298 K) and η is the viscosity of the solvent (η = cP = 0.001 N s/m^2). Viscosities were measured at a regulated internal temperature of 298 K.

Viscosity Measurements: Viscosity measurements were carried out using a calibrated RheoSense µVISC viscometer (0.01–100 cP) with a temperature controller (18–50 °C). Measurements were repeated a minimum of three times with the average viscosity reported and the standard deviation displayed as error bars.

2. Screening for a Porous Liquid

2.1 General Procedure for Synthesis of Scrambled cages:

To a solution of 1,3,5-triformylbenzene (300 mg, 1.85 mmol, 4 eq.) in DCM (90 mL) was added a solution of 1,2-diamino-2-methylpropane, for the **CC13** component, in DCM (9 mL) and a solution of one of: ethylenediamine (for **CC1**), (*R*)-(+)-1,2-diaminopropane dihydrochloride (for **CC2**-*R*), 1,2-diaminopropane (for *rac*-**CC2**), (*R,R*)-1,2-diaminocyclohexane (for **CC3**-*R*) or 1,2-diaminocyclohexane (for *rac*-**CC3**) in DCM (9 mL).

Quantities of each diamine were calculated using $x^{6-n}:13^n$ where *x* represents the non-**CC13** diamine used, and *n* represents the equivalents of the **CC13** diamine used, i.e. the number of **CC13** vertices, with all possible ratios being conducted (1:5, 2:4, 3:3, 4:2, 5:1).

The reactions were stirred at rt for 3 days before concentration *in vacuo*. To the residue was added DCM (10 mL) and any insoluble precipitates removed by filtration before re-concentration of the filtrate *in vacuo*. For samples where the hydrochloride salt of a diamine had been used, the residue was then dissolved in THF (50 mL) and the insoluble triethylamine-hydrochloride salts removed by filtration before concentration of the filtrate *in vacuo*. The purified scrambled cages were dried in the vacuum oven at 40 °C overnight and analysed by ^{1}H NMR, HPLC, and PXRD.

2.2 General Procedure for Solubility Screen of scrambled cages in bulky solvents:

For solubility testing in $CHCl_3$: Scrambled cage (desolvated in the vacuum oven at 90 °C overnight) was diluted with $CHCl_3$ (pre-treated with anhydrous K_2CO_3 overnight) until a saturated solution formed. The saturated solution was stirred at rt for 2 h to ensure no further cage dissolved before being filtered through a 0.2 µm PTFE syringe filter. A 100 µL sample of each filtered solution was added to a pre-weighed vial, followed by a 100 µL $CHCl_3$ syringe wash. Samples had $CHCl_3$ removed under a N_2 flow before being dried in the vacuum oven at 90 °C overnight. Samples of cages re-weighed to enable calculation of the solubility in terms of mg/mL.

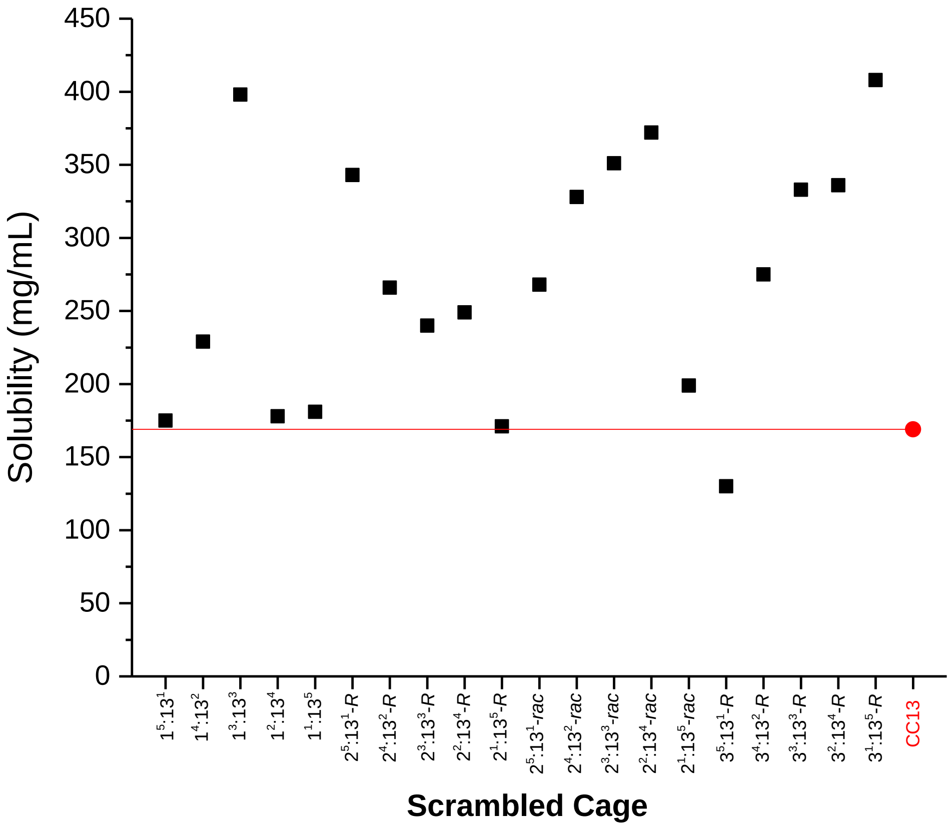

Supplementary Fig. 1: Solubility testing of scrambled cages in $CHCl_3$ compared to our most soluble 'unscrambled' cage to date, **CC13**, demonstrating that in almost all cases the scrambled mixtures have increased solubility.

For solubility testing in bulky solvents: Approximately 10 mg of each scrambled cage (desolvated in the vacuum oven at 90 °C overnight) was added to pre-weighed vials equipped with stirrer bars, and the weights recorded. Initially 0.3 mL of the solvent being tested was added and the weight recorded before stirring the sample at rt. If the solid fully dissolved, more sample was added recording the weight after each addition until no further solid could be dissolved. If the solid did not fully dissolve, more solvent was added in 0.1 mL aliquots until no solid remained with the weight being recorded, unless the solubility dropped below 10 mg/mL at which point the sample was discarded. The solubility of each sample was calculated in terms of mg/mL and the molar ratio of solvent:cage.

Supplementary Table 1: Solubility testing of scrambled cages in bulky solvents (Bmim.BF_4 = 1-butyl-3-methylimidazolium tetrafluoroborate; PERC = perchloroethylene).

**Scrambled Cage**[a]	**Solubility** (mg/mL, solvent:cage molar ratio)					
	**15-crown-5**	**1,2,4-trichloro-Benzene**	**Bmim.BF_4**	**PERC**	**PCP**	**Benzyl benzoate**
**CC1^5:13^1**	<9.9, >417.0:1	12.2, 538.8:1	<9.8 >447.7:1	<8.3 >972.1:1	<11.0 >487.5:1	<7.5 >578.7:1
**CC1^4:13^2**	<9.5, >451.3:1	<16.5, >412.4:1	<8.8, >516.4:1	<8.1, >1025.0:1	<9.9, >557.3:1	15.1, 296.8:1
**CC1^3:13^3**	<8.8, >501.9:1	<9.3, >753.3:1	<10.5, >447.8:1	<7.4, >1157.7:1	<6.5, >877.3:1	<10.1, >457.7:1
**CC1^2:13^4**	<10.9, >419.2:1	14.7, 493.6:1	<11.5, >419.8:1	<8.6, >1025.8:1	<14.3, >414.3:1	<10.9, >435.8:1
**CC1^1:13^5**	<11.3, >417.8:1	17.6, 424.8:1	<9.7, >517.6:1	<10.1, >902.2:1	<7.2, >849.9:1	12.9, 380.1:1
**CC2^5:13^1**-*R*	<21.4, >210.6:1	71.7, 99.5:1	<14.0, >340.1:1	23.5, 370.5:1	71.8, 88.0:1	45.3, 103.5:1
**CC2^4:13^2**-*R*	<17.5, >261.8:1	44.5, 162.9:1	<10.1, >478.6:1	20.9, 424.0:1	89.2, 71.9:1	51.9, 91.0:1
**CC2^3:13^3**-*R*	<17.3, >268.0:1	62.3, 118.1:1	<8.2, >600.8:1	30.3, 297.1:1	155.6, 41.9:1	60.1, 80.5:1
**CC2^2:13^4**-*R*	<19.2, >245.3:1	88.0, 84.9:1	<9.4, >533.0:1	11.5, 795.9:1	62.3, 106.3:1	77.7, 63.2:1
**CC2^1:13^5**-*R*	-	-	-	-	37.1, 181.2:1	95.9, 52.0:1

Scrambled Cage[a]	Solubility (mg/mL, solvent:cage molar ratio)					
	**15-crown-5**	**1,2,4-trichloro-Benzene**	**Bmim.BF_4**	**PERC**	**PCP**	**Benzyl benzoate**
**CC2**^{5}:**13**^{1}-*rac*	11.8, 382.8:1	12.8, 556.9:1	<11.2, >425.1:1	<7.7, >1133.1:1	<7.3, >800.5:1	<10.9, >430.1:1
**CC2**^{4}:**13**^{2}-*rac*	<10, 468.6:1	11.2, 648.3:1	<12.3, >393.3:1	<7.7, >1153.8:1	<9.0, >653.7:1	<8.2, >582.4:1
**CC2**^{3}:**13**^{3}-*rac*	<9.9, >468.2:1	<7.5, >986.4:1	<8.2, >603.4:1	<11.1, >810.5:1	<7.1, >917.2:1	<6.8, >709.8:1
**CC2**^{2}:**13**^{4}-*rac*	11.8, 400.9:1	17.3, 431.7:1	<11.3, >443.8:1	<8.2, >1119.6:1	12.0, 552.9:1	16.1, 305.8:1
**CC2**^{1}:**13**^{5}-*rac*	9.6, 499.2:1	12.9, 589.3:1	<11.9, >425.3:1	<11.6, >797.5:1	<11.4, >544.2:1	<12.4, >403.1:1
**CC3**^{5}:**13**^{1}-*R*	<8.1, >677.6:1	32.3, 270.8:1	<14.5, >249.7:1	9.1, 1172.0:1	<9.2, >775.6:1	43.4, 132.3:1
**CC3**^{4}:**13**^{2}-*R*	7.9, 678.8:1	24.2, 352.6:1	<19.7, >179.0:1	9.3, 1126.2:1	31.8, 237.0:1	52.4, 107.1:1
**CC3**^{3}:**13**^{3}-*R*	33.6, 156.1:1	34.9, 238.3:1	<18.9, >182.1:1	9.2, 1105.7:1	242, 30.4:1	160.5, 34.1:1
**CC3**^{2}:**13**^{4}-*R*	12.8, 401.0:1	10.7, 760.1:1	<20.7, >161.9:1	<16.0, >619.8:1	50.6, 141.9:1	73.3, 72.7:1
**CC3**^{1}:**13**^{5}-*R*	14.6, 342.1:1	18.6, 424.2:1	<13.7, >239.3:1	<15.6, >617.7:1	28.8, 243.2:1	17.7, 294.3:1
**CC3**^{5}:**13**^{1}-*rac*	<22.8, >241.4:1	39.2, 223.2:1	<13.9, >421.8:1	12.4, 861.5:1	<14.5, >534.7:1	<9.6, >599.7:1
**CC3**^{4}:**13**^{2}-*rac*	<18.9, >284.4:1	51.9, 164.5:1	<9.8, >582.7:1	32.6, 319.3:1	<8.3, >909.3:1	<9.8, >572.4:1
**CC3**^{3}:**13**^{3}-*rac*	<17.5, >300.4:1	69.2, 120.4:1	<11.3, >490.2:1	44.0, 231.3:1	<9.0, >816.8:1	<37.0, >147.8:1
**CC3**^{2}:**13**^{4}-*rac*	<28.4, >180.2:1	75.6, 107.4:1	<11.9, >456.6:1	77.3, 128.2:1	209.8, 34.2:1	99.1, 53.8:1
**CC3**^{1}:**13**^{5}-*rac*	<18.0, >276.9:1	90.1, 87.7:1	<14.5, >365.3:1	186.2, 51.8:1	195.0, 35.9:1	142.7, 36.4:1

[a] Scrambled cage name based on ratio of diamines used

3. Synthesis, Purification and Characterisation

3.1 Synthesis of Scrambled Cage Mixture 3^3:13^3-*R*

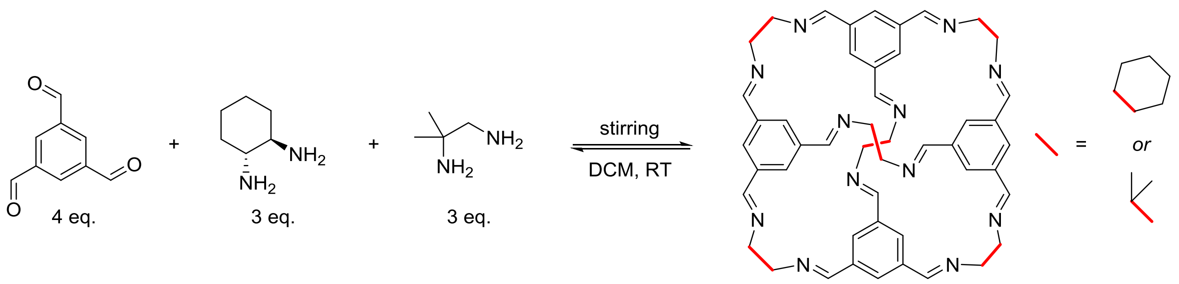

Three batches of scrambled cage were produced by the following procedure:

To a 3 L jacketed vessel, equipped with overhead stirrer, was added 1,3,5-triformylbenzene (7.5 g, 46.25 mmol, 4 eq.) in DCM (2.25 L), followed by solutions of (*R,R*)-1,2-diaminocyclohexane (3.961 g, 34.69 mmol, 3 eq.) in DCM (225 mL) and 1,2-diamino-2-methylpropane (3.058 g, 34.69 mmol, 3 eq.) in DCM (225 mL). The resulting solution was stirred at 20 °C under N_2 for 3 days and the reaction completion checked by HPLC analysis before concentration *in vacuo*. This preparation was repeated and both crude reaction products were redissolved in DCM (250 mL), combined, and filtered to remove any insoluble solids before concentration *in vacuo*. The resulting yellow solid was washed with EtOAc (3 x 100 mL) and the collected solid completely dissolved in DCM before concentration *in vacuo* prior to drying in the vacuum oven at 90 °C overnight to afford the scrambled **3^3:13^3**-*R* cage mixture as a very pale yellow solid (Batch 1: 14.71 g, 61%; Batch 2: 14.54 g, 60%; Batch 3: 9.33 g, 77% (*NB* preparation only conducted once for batch 3)).

Mpt >240 °C (decomposed before melting)*;* **IR** (ν_{max} /cm^{-1}) 2927, 2856, 1648 (imine, N=C), 1598, 1444, 1380, 1365, 1147, 1093, 1054; **^{1}H NMR** (400 MHz, $CDCl_3$) δ_H 8.22–7.78 (24H, m, N=C*H* and Ar*H*), 3.95–3.35 (12H, m, C*H*N=CH), 1.84–1.31 (42H, m, CH_2 and CH_3); **^{13}C NMR** (101 MHz, $CDCl_3$) δ_C (*NB.* Due to scrambling all ^{13}C NMR signals are broad mulitplets) 160.9, 159.2, 155.3, 137.1, 136.6, 129.4, 74.8, 72.3, 61.2, 33.1, 29.5, 24.4, 22.2; **HRMS** (ES+) calc. for scrambled cages 3^0:13^6, 3^1:13^5, 3^2:13^4, 3^3:13^3, 3^4:13^2, 3^5:13^1, 3^6:13^0 = 960.6003, 986.6159, 1012.6316, 1038.6472, 1064.6629, 1090.6785, 1116.6942; found $[M+H]^+$ 961.6164, 987.6344, 1013.6505, 1039.6669, 1065.6821, 1091.6965 and $[M+2H]^{2+}$ 559.3612; **CHN Analysis** calc. for $C_{66}H_{78}N_{12}$: C, 76.27; H, 7.56; N, 16.17; found: C, 74.38; H, 7.32; N, 15.66.

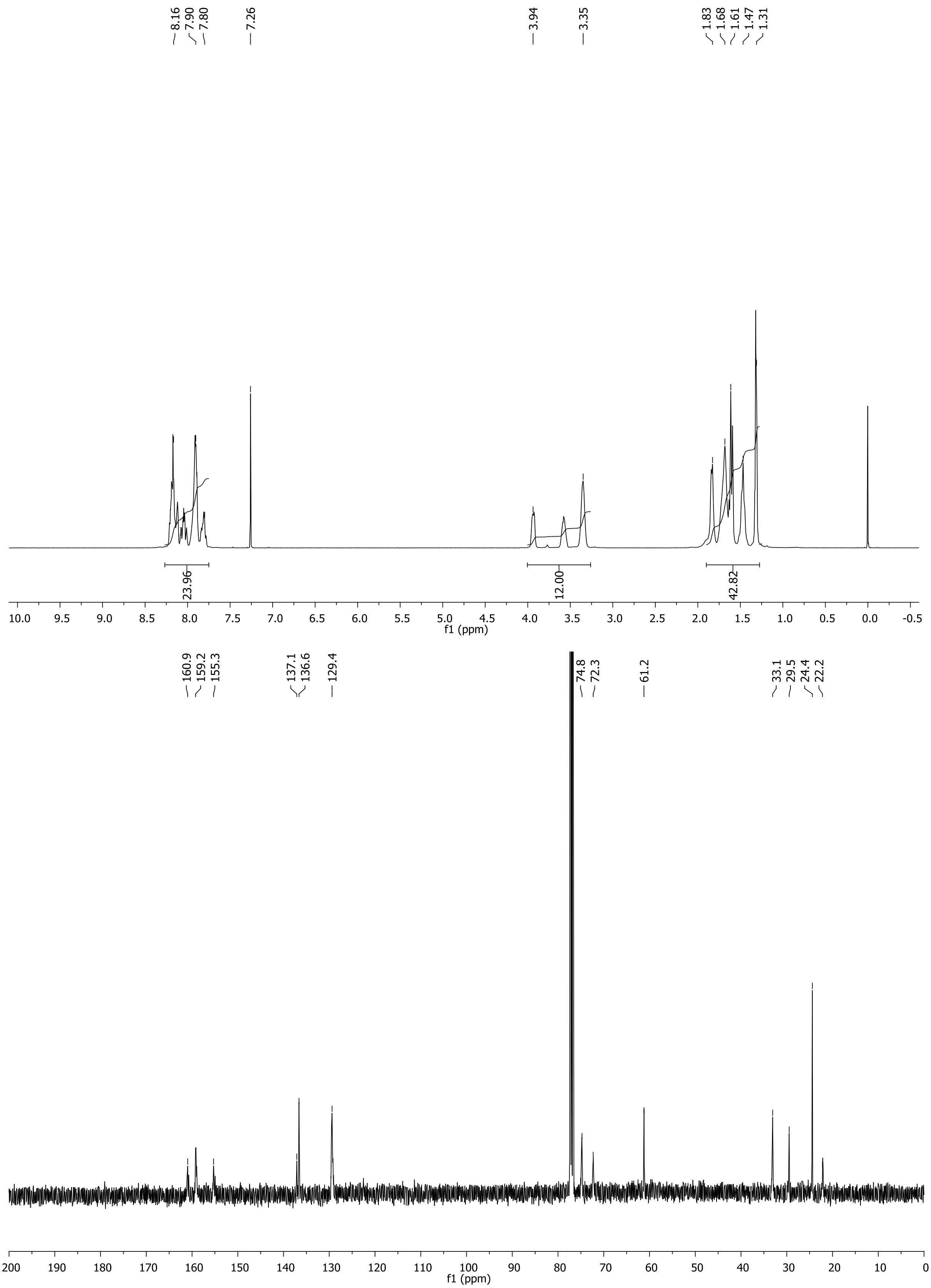

Supplementary Fig. 2: ^{1}H NMR ($CDCl_3$; upper) and ^{13}C NMR ($CDCl_3$; lower) spectra of $\mathbf{3^3{:}13^3}$-*R* scrambled cage mixture.

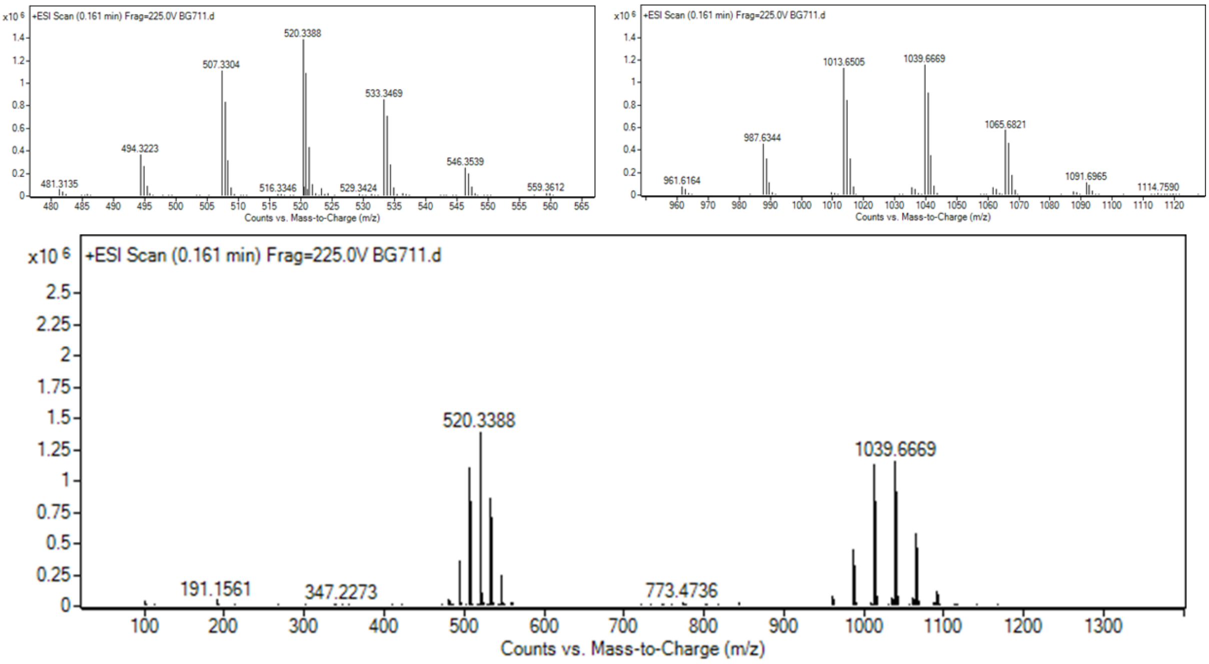

Supplementary Fig. 3: QTOF mass spectrometry of the **3^3:13^3**-*R* scrambled cage mixture.

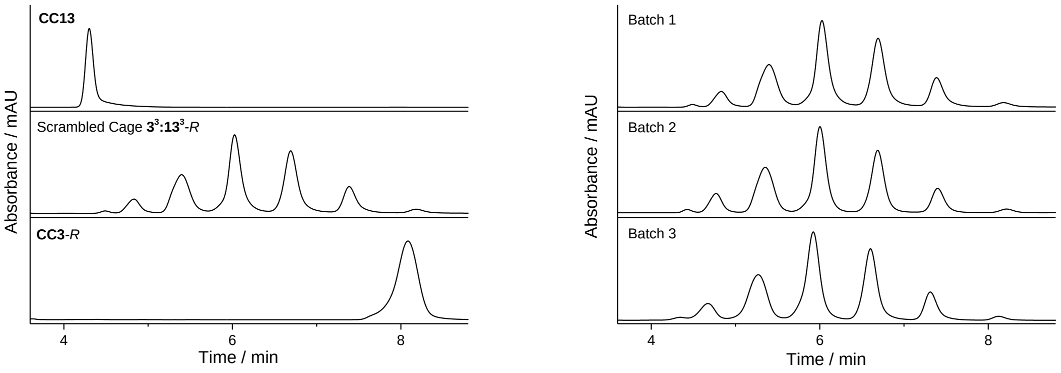

Batch	Peak Area / %						
	$3^0 13^6$	$3^1 13^5$	$3^2 13^4$	$3^3 13^3$	$3^4 13^2$	$3^5 13^1$	$3^6 13^0$
1	0.61	6.67	21.50	33.11	26.32	10.36	1.43
2	0.86	7.21	22.76	33.08	24.98	9.71	1.39
3	0.35	5.55	21.98	34.54	26.71	9.44	1.42

Supplementary Fig. 4: HPLC Analysis - Comparison of the parent cages **CC3**-*R* and **CC13** with the scrambled **3^3:13^3**-*R* cage mixture (upper left), alongside an overlay of the HPLC traces of the different scrambled cage batches (upper right) with the corresponding peak areas for each cage component.

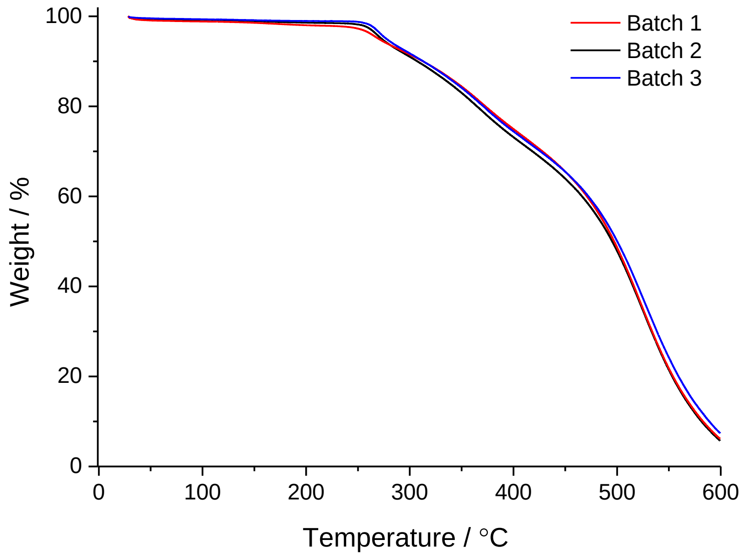

Supplementary Fig. 5: Overlaid thermogravimetric (TGA) data for the different batches of the scrambled **3^3:13^3**-*R* cage mixture.

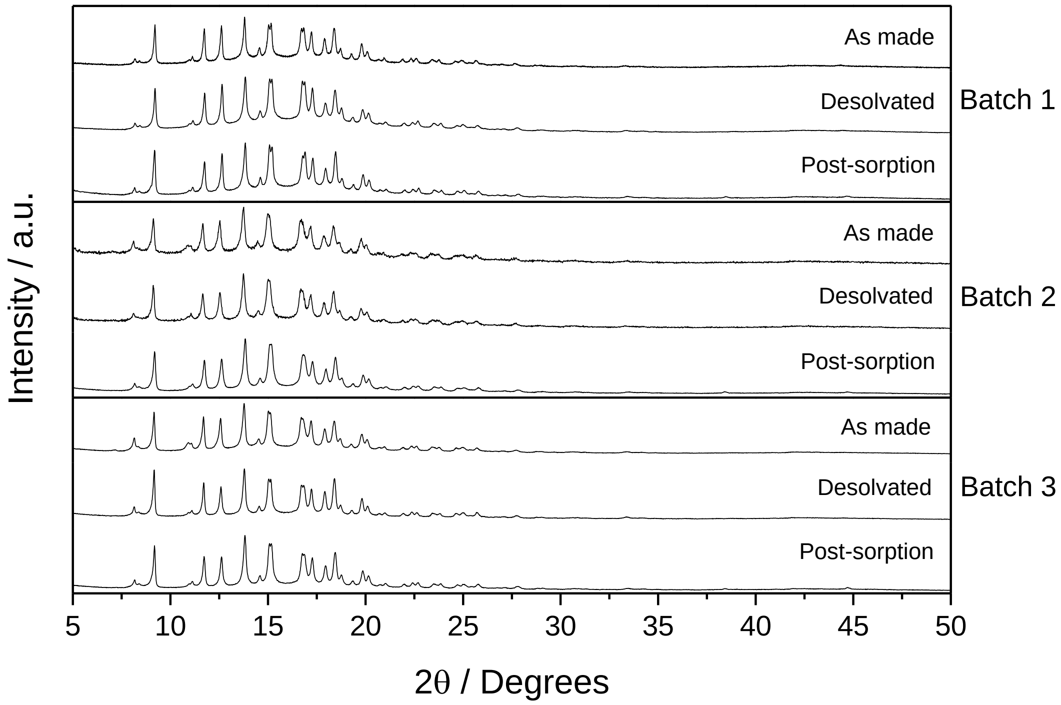

Supplementary Fig. 6: PXRD patterns for the different batches of scrambled **3^3:13^3**-*R* cage mixture all showing a degree of crystallisation that remains the same whether the scrambled cage mixture is as-made, desolvated at 90 °C in a vacuum oven or post-sorption.

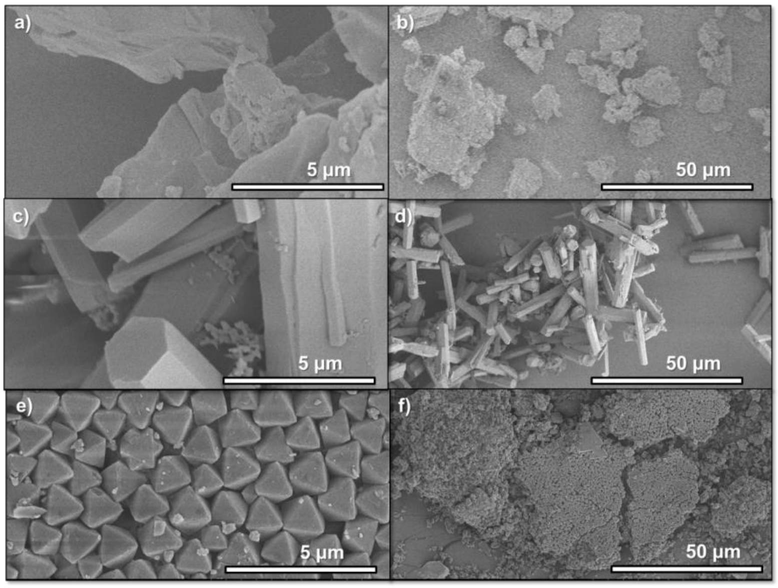

Supplementary Fig. 7: Scanning electron micrographs of cage samples: (a), (b) - scrambled **3^3:13^3**-*R* cage showing no clear geometric order; (c), (d) - **CC13**α can be seen to form hexagonal needle crystals; (e), (f) – **CC3**α can be seen to form octahedral crystals. Samples are shown with a 5 µm scale bar (left-hand column) or 50 µm scale bar (right-hand column).

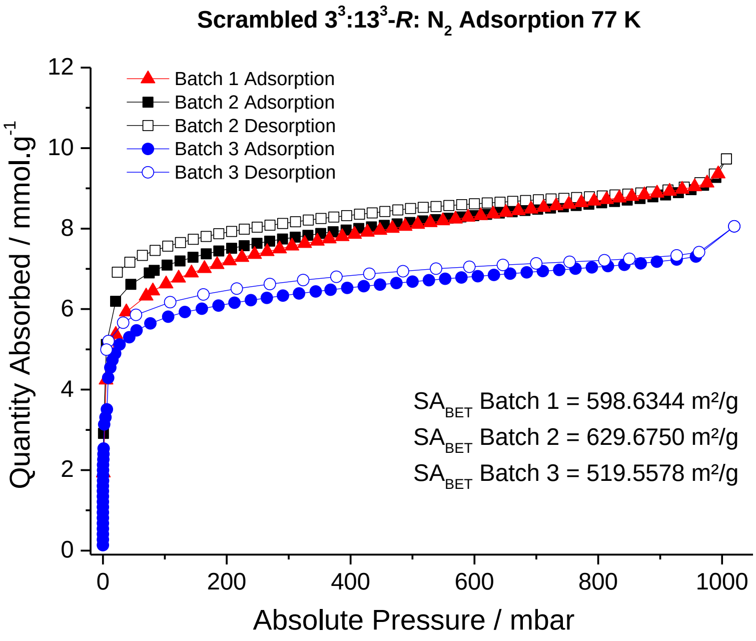

Supplementary Fig. 8: Nitrogen adsorption / desorption isotherms for the different batches of scrambled **3^3:13^3**-*R* cages (77 K) as a function of pressure alongside their respective surface areas. The differences in surface area are likely due to the difference in packing of the scrambled cages in the solid state between batches; this difference has no effect on the gas uptake of the PL between batches.

3.2 Synthesis of 'Control' Molecules

1,1',1''-(Benzene-1,3,5-triyl)tris(*N*-cyclohexylmethanimine), (SIC)

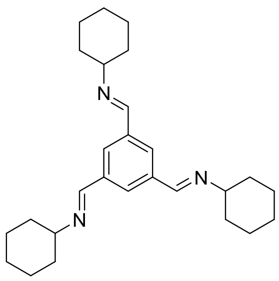

To a solution of 1,3,5-triformylbenzene (1 g, 6.16 mmol, 1 eq.) in DCM (100 mL) was added cyclohexylamine (2.12 mL, 18.50 mmol, 3 eq.) and the resulting solution stirred at rt overnight before being concentrated *in vacuo*. The resulting solid was dried in the vacuum oven at 90 °C until all traces of residual DCM had been removed to afford the desired product as an off-white solid (2.38 g, 95%).

Mpt 106–108 °C*;* **IR** (ν_{max} /cm^{-1}) 2930, 2843, 1649 (imine, N=C), 1454, 1346, 1161, 1070, 964, 892; **^{1}H NMR** (500 MHz, $CDCl_3$) δ_H 8.36 (3H, s, N=C*H*), 8.10 (3H, s, Ar*H*), 3.19 (3H, tt, *J* = 10.5 Hz, 4.0 Hz, NC*H*), 1.82 (6H, dt, *J* = 13.0 Hz, 3.0 Hz, *c*Hex), 1.69 (9H, m, *c*Hex), 1.57 (6H, qd, *J* = 13.0, 3.5 Hz, *c*Hex), 1.36 (6H, qt, *J* = 12.5, 3.0 Hz, *c*Hex), 1.24 (3H, m, *c*Hex); **^{13}C NMR** (126 MHz, $CDCl_3$) δ_C 157.9, 137.5, 129.3, 70.1, 34.4, 25.7, 24.9; **HRMS** (CI+) calc. for C27H40N3 [M+H]$^+$ 406.3222, found 406.3226; **CHN Analysis** calc. for $C_{27}H_{39}N_3$: C, 79.95; H, 9.69; N, 10.36; found: C, 79.92; H, 9.71; N, 10.34.

Reduced Scrambled Cage Mixture red-$\mathbf{3^3{:}13^3}$-*R*

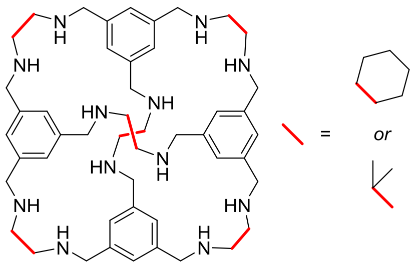

To a solution of scrambled $\mathbf{3^3{:}13^3}$-*R* cage mixture (1.28 g, 1.23 mmol, 1.0 eq.) in a $CHCl_3$/MeOH mix (75 mL, 1:1), was added sodium borohydride (1.49 g, 39.41 mmol, 32.0 eq.) batchwise under N_2. After complete addition, reaction stirred at rt overnight before being concentrated *in vacuo*. The resulting solid was partitioned between $CHCl_3$ (200 mL) and water (100 mL), before the organic layer was subsequently washed with water (100 mL), dried ($MgSO_4$) and concentrated *in vacuo* to afford the reduced-$\mathbf{3^3{:}13^3}$-*R* scrambled cage mixture as an off-white solid (1.3 g, 1.22 mmol, quant.) which was used without further purification.

Mpt 102–118 °C; **IR** (ν_{max} /cm^{-1}) 2922, 2852, 1733, 1603, 1448, 1361, 1241, 1112; **^{1}H NMR** (500 MHz, $CDCl_3$) δ_H (*NB.* Due to the flexible mixture of reduced scrambled cages, signals are broad mulitplets) 7.65–6.59 (12H, m, Ar*H*), 4.05–3.22 (24H, m, NHC*H*$_2$Ar), 2.66–0.64 (66H, m, N*H*, C*H*, C*H*$_2$ and C*H*$_3$); **^{13}C NMR** (126 MHz, $CDCl_3$) δ_C (*NB.* Due to scrambling most ^{13}C NMR signals are broad mulitplets) 174.4, 171.0, 141.2, 125.9, 74.5, 61.3, 53.2, 51.5, 50.7, 46.3, 34.2, 31.7, 29.5, 25.1, 22.6, 21.4, 14.1; **HRMS** (ES+) calc. for reduced scrambled cages red-$3^0{:}13^6$, red-$3^1{:}13^5$, red-$3^2{:}13^4$, red-$3^3{:}13^3$, red-$3^4{:}13^2$, red-$3^5{:}13^1$, red-$3^6{:}13^0$ = 984.7881, 1010.8037, 1036.8194, 1062.8350, 1088.8507, 1114.8663, 1140.8820; found [M+H]$^+$ 985.7911, 1011.8107, 1037.8283, 1063.8441, 1089.8611, 1115.8790, 1141.8984.

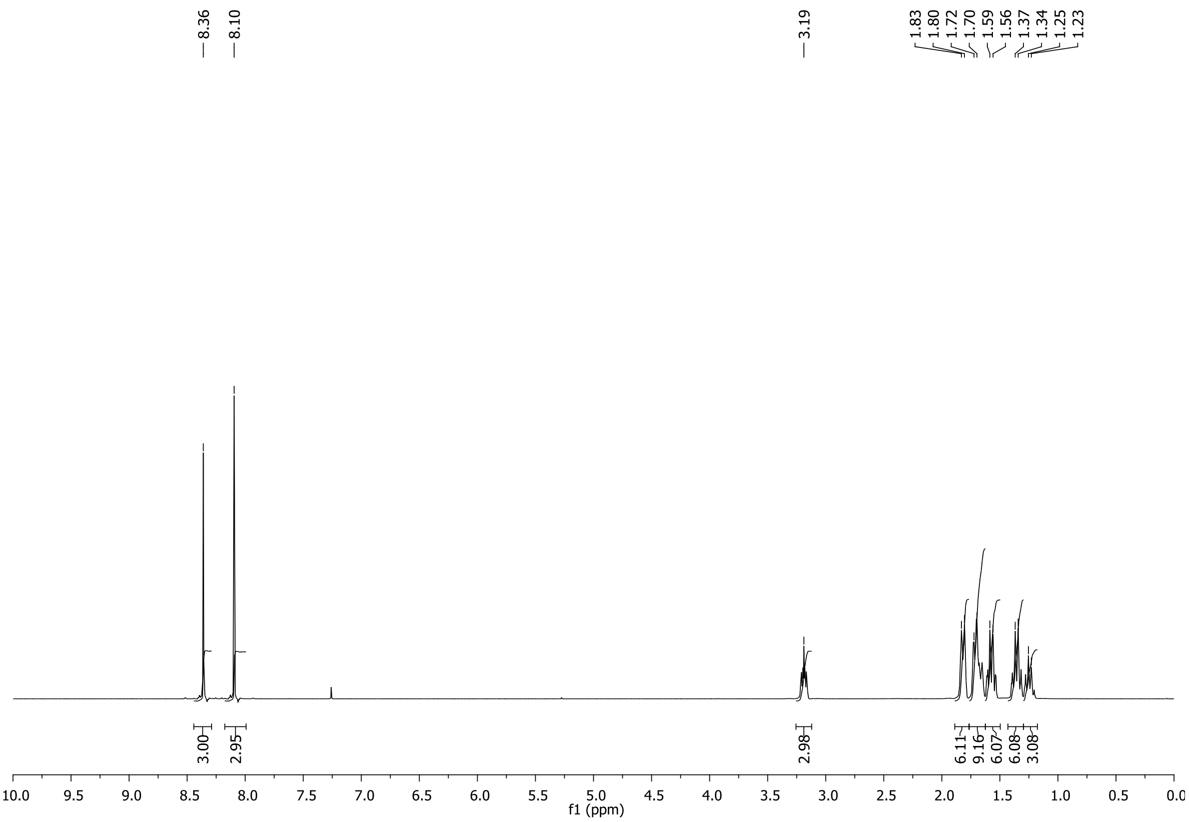

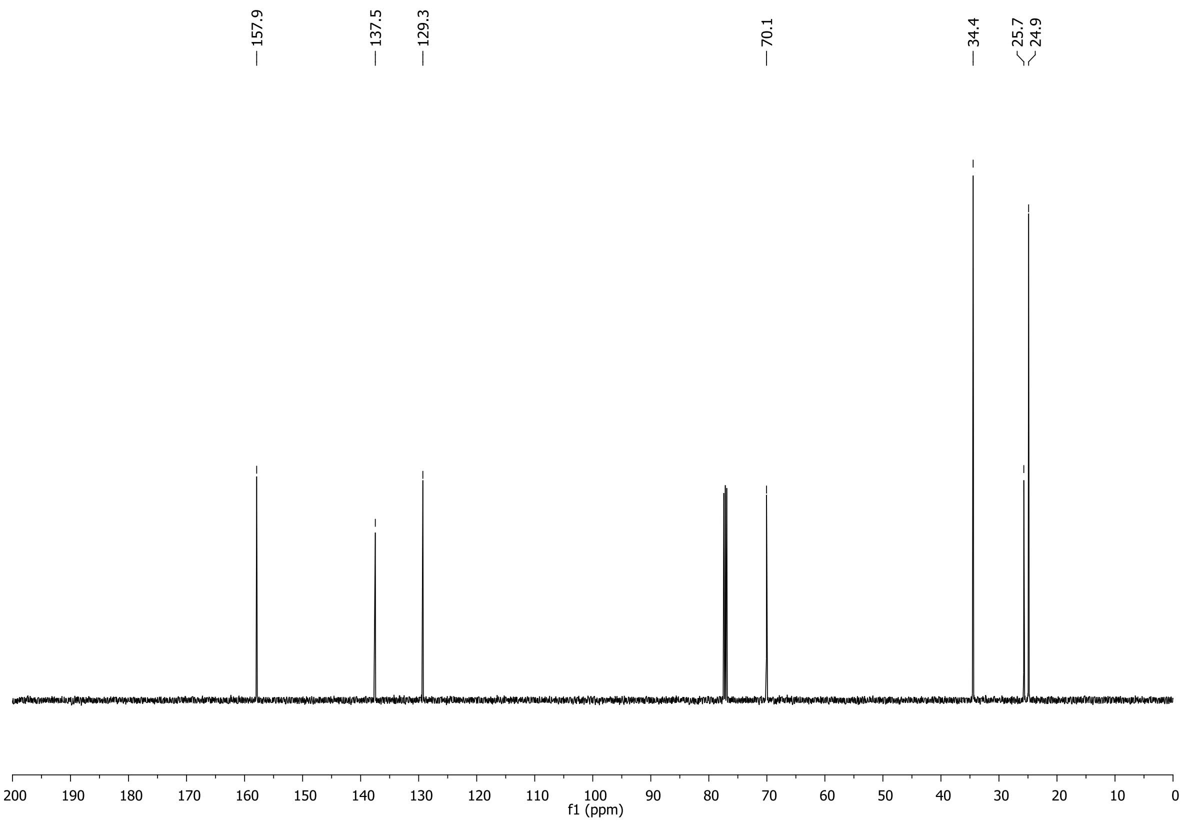

Supplementary Fig. 9: ^{1}H NMR ($CDCl_3$; upper) and ^{13}C NMR ($CDCl_3$; lower) spectra of 1,1',1''-(benzene-1,3,5-triyl)tris(*N*-cyclohexylmethanimine).

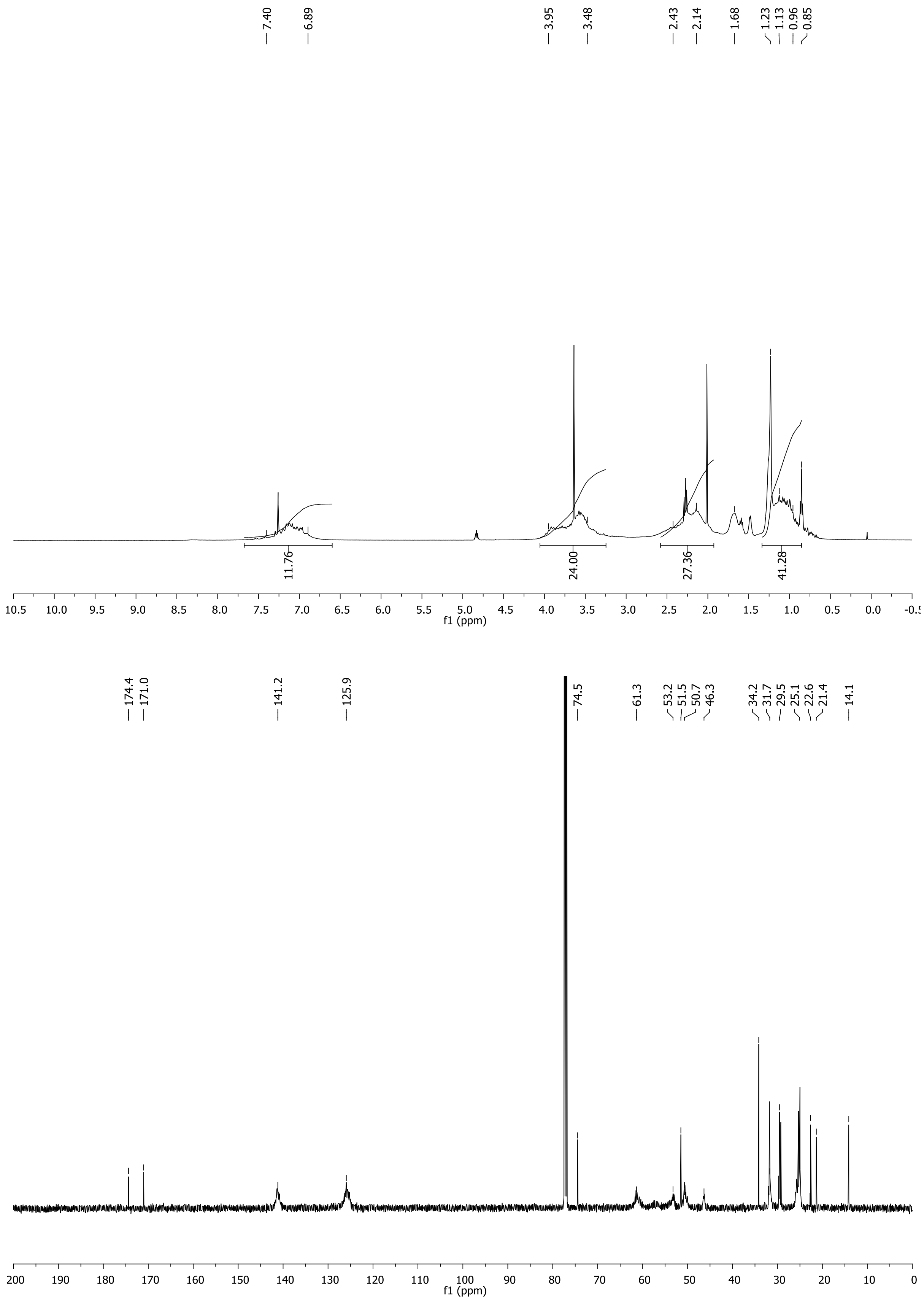

Supplementary Fig. 10: ^{1}H NMR ($CDCl_3$; upper) and ^{13}C NMR ($CDCl_3$; lower) spectra of the crude reduced-**3^3:13^3**-*R* scrambled cage mixture.

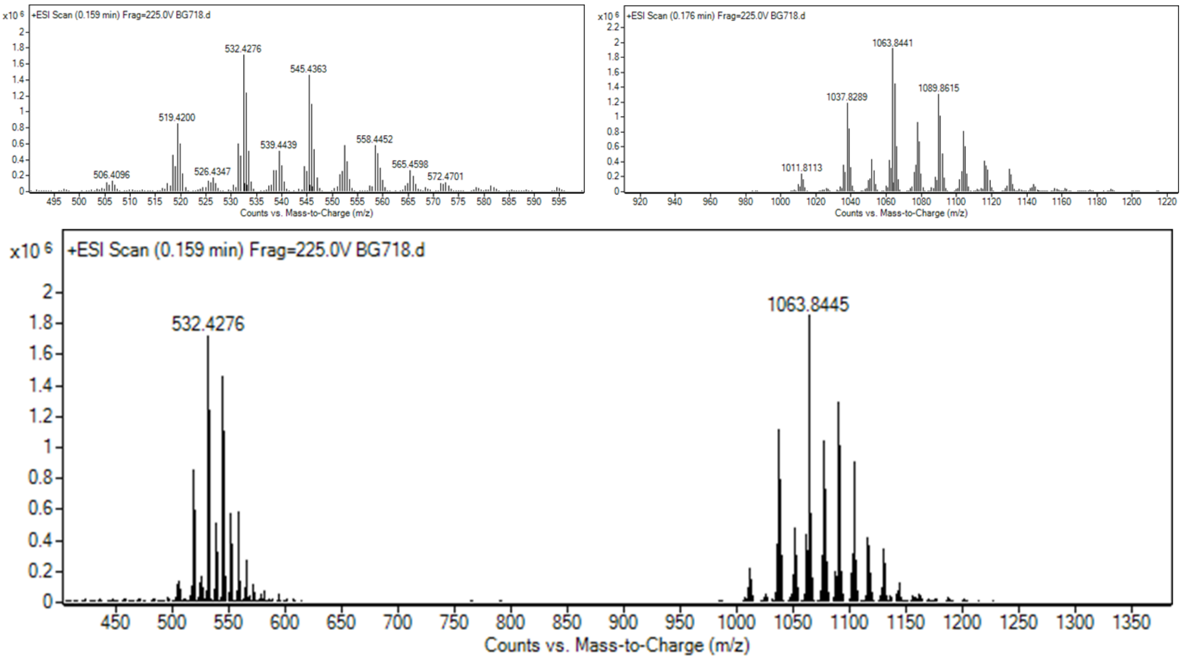

Supplementary Fig. 11: QTOF mass spectrometry of the reduced-$\mathbf{3^3}$:$\mathbf{13^3}$-*R* scrambled cage mixture.

3.3 Purification of Solvents

Hexachloropropene (PCP): It is worth noting that different batches of PCP often had markedly different impurity profiles. The properties of the PL could be affected by these impurities, especially smaller-sized impurities which could act as a competitive guest in the cage cavities. Therefore, the solvent was thoroughly purified and analysed before use.

PCP (~510 mL, 9 x 100 g bottles, Sigma-Aldrich, H6401, batch MKBQ2222V) was filtered 5 times through 5 separate activated basic alumina plugs (5 x 150 g Aluminium oxide, activated, basic, Brockmann I, CAS 1344-28-1, Sigma-Aldrich) using positive N_2 pressure to afford pure PCP (175 mL, 34% recovery); **^{13}C NMR** (126 MHz, d_2-DCM/TMS capillary) δ_C 132.1, 127.2, 92.8.

The purified PCP was transferred to a dry Schlenk tube and degassed *via* repeated freeze-pump-thaw cycles before being stored under N_2 and used for the PL studies. Unless stated otherwise, all experiments used this purified solvent.

***Safety Note* — PCP is fatal by inhalation and a lachrymator. Therefore it is important that all manipulations using this solvent are conducted in a fume cupboard, with all samples for measurements being appropriately sealed before removal.**

1-*t*-Butyl-3,5-dimethylbenzene: Purified by vacuum distillation with the first 10% discarded and pure 1-*t*-butyl-3,5-dimethylbenzene collected at 55 °C at a pressure of 1.7 mbar, and stored over 3Å molecular sieves under a N_2 atmosphere.

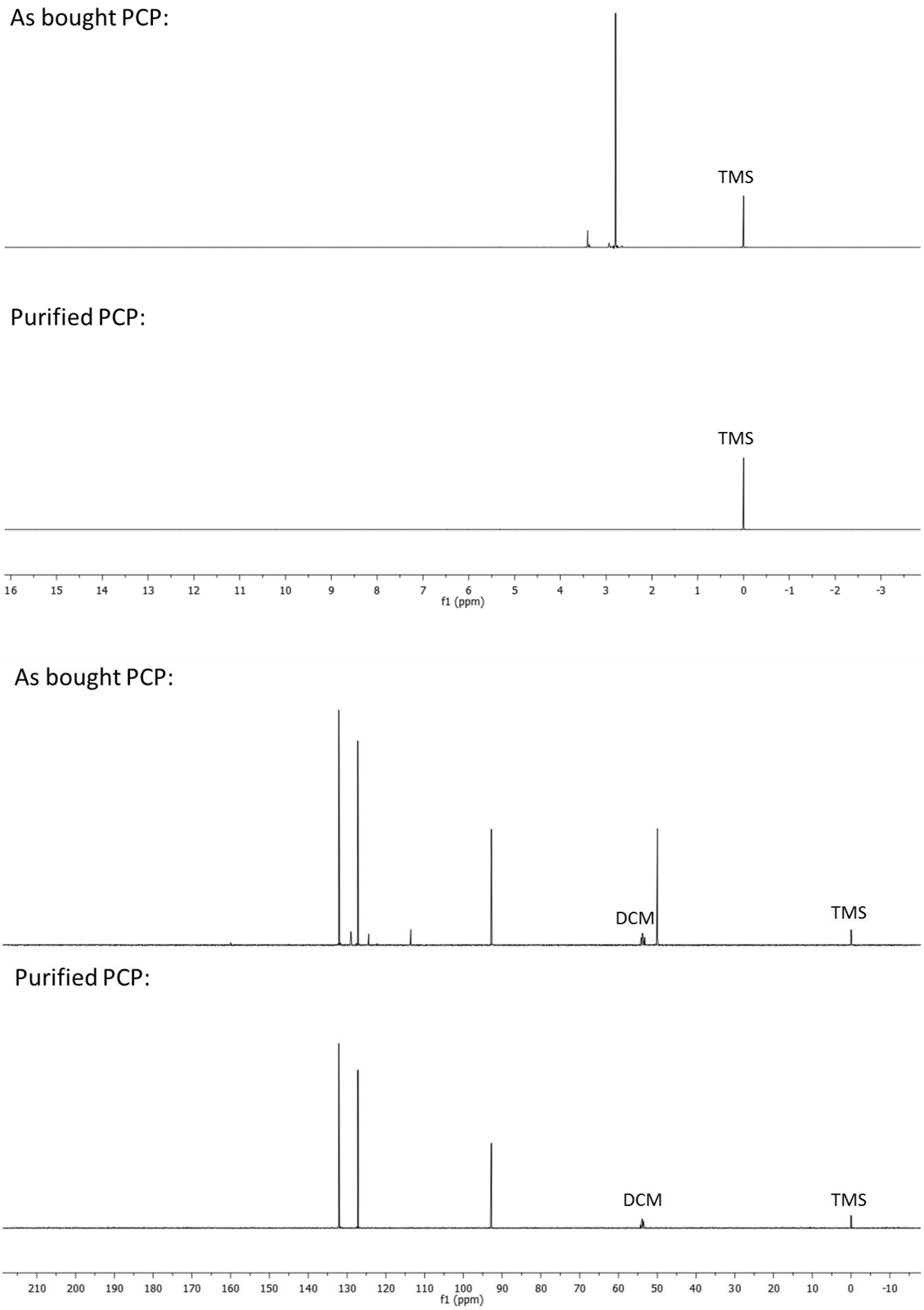

Supplementary Fig. 12: Stacked ^{1}H NMR (d_2-DCM/TMS capillary; upper) and ^{13}C NMR (d_2-DCM/TMS capillary; lower) spectra of PCP—before and after purification through basic alumina.

3.4 Standard Procedures for Preparing the Liquid Samples for Testing

Preparation of the Scrambled Porous Liquid (PL): Scrambled $\mathbf{3^3}$:$\mathbf{13^3}$-*R* cage mixture desolvated in a vacuum oven at 90 °C overnight before being evacuated and refilled with N_2 on a manifold in an oven dried GC headspace vial. The scrambled $\mathbf{3^3}$:$\mathbf{13^3}$-*R* cage subsequently had degassed PCP added at a concentration of 20% w_{cage}/v_{PCP} (e.g. 200 mg dissolved in 1 mL PCP, 600 mg in 3 mL), the N_2 line removed and was stirred or vortexed until fully dissolved to afford a pale yellow liquid.

Preparation of PCP: An oven dried GC headspace vial was evacuated and refilled with N_2 on a manifold before the addition of degassed PCP and removal of the N_2 line.

Preparation of Control Non-Porous Liquid (Non-PL): Prepared according to the method for the PL but using the small imine control (SIC), 1,1',1''-(benzene-1,3,5-triyl)tris(*N*-cyclohexylmethan-imine).

Preparation of Reduced-Scrambled Liquid (Red-PL): Prepared according to the method for the PL but using the reduced scrambled cage mixture red-$\mathbf{3^3}$:$\mathbf{13^3}$-*R* and desolvation carried out at 80 °C.

.

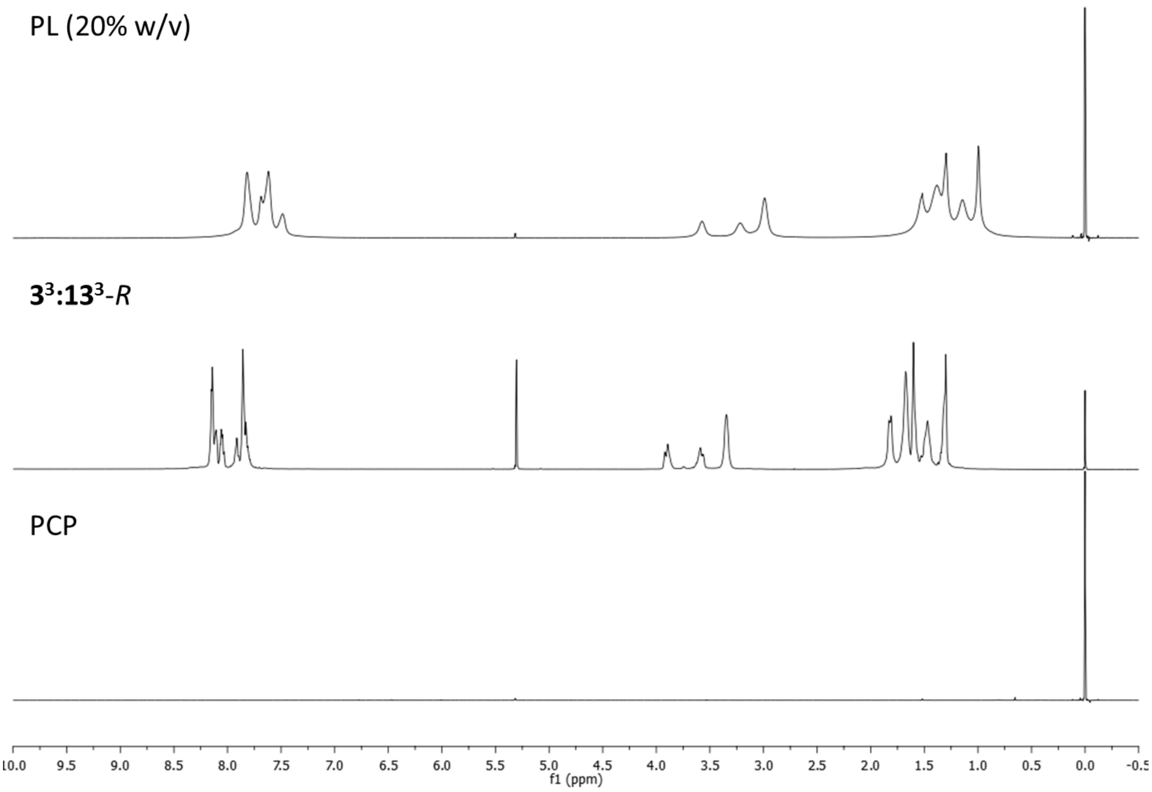

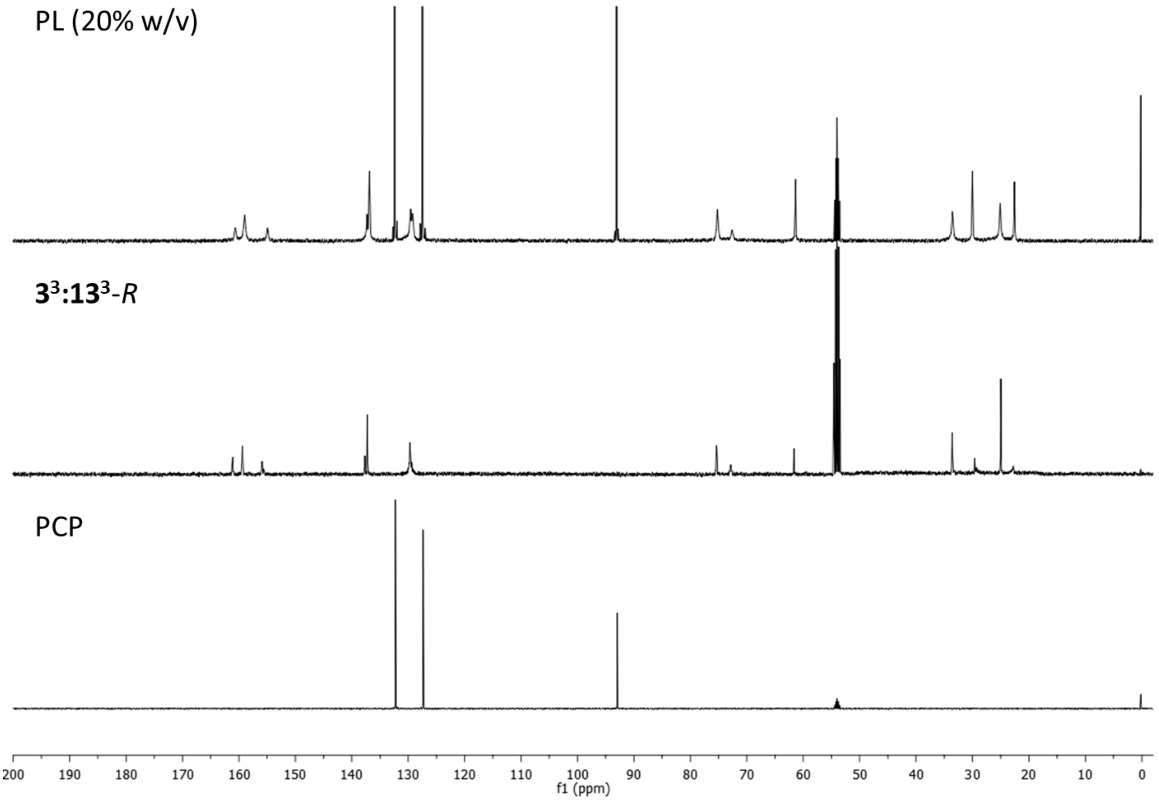

Supplementary Fig. 13: Stacked ^{1}H NMR (d_2-DCM/TMS capillary; upper) and ^{13}C NMR (d_2-DCM/TMS capillary; lower) spectra of the PL (20% w/v, top), scrambled cage mixture (**3^3:13^3**-*R*, middle) and PCP (bottom).

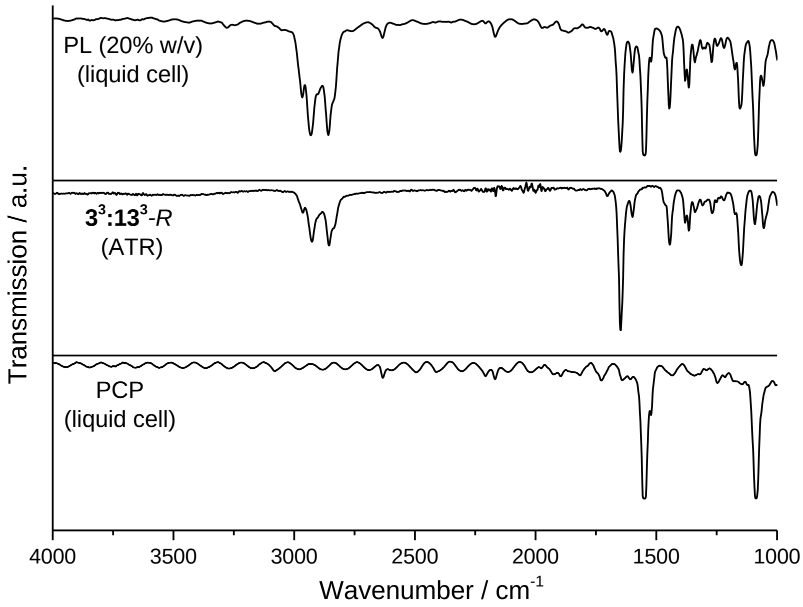

Supplementary Fig. 14: FTIR Analysis—Comparison of the transmission IR spectra of the PL (20% w/v, upper), scrambled cage mixture (**3^3:13^3**-*R*, middle) and PCP (lower) showing the 1649 cm^{-1} band of the cage imine bond and the 1548 cm^{-1} band of the PCP alkene bond. The IR of the PL and PCP were measured using a liquid cell, and the IR of the solid scrambled **3^3:13^3**-*R* cage mixture was measured using an ATR module.

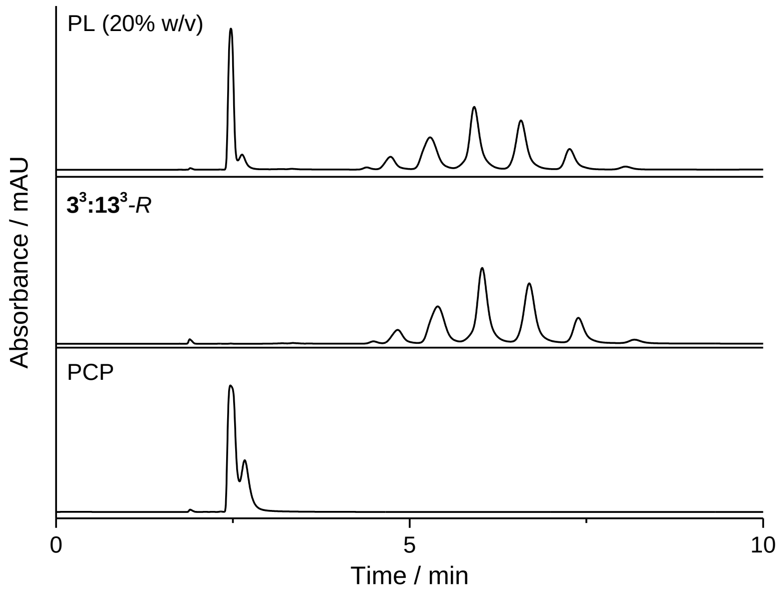

Supplementary Fig. 15: HPLC Analysis—Comparison of the PL (20% w/v, upper), scrambled cage mixture (**3^3:13^3**-*R*, middle) and PCP (lower) confirming the scrambled cage is still present and stable in PCP.

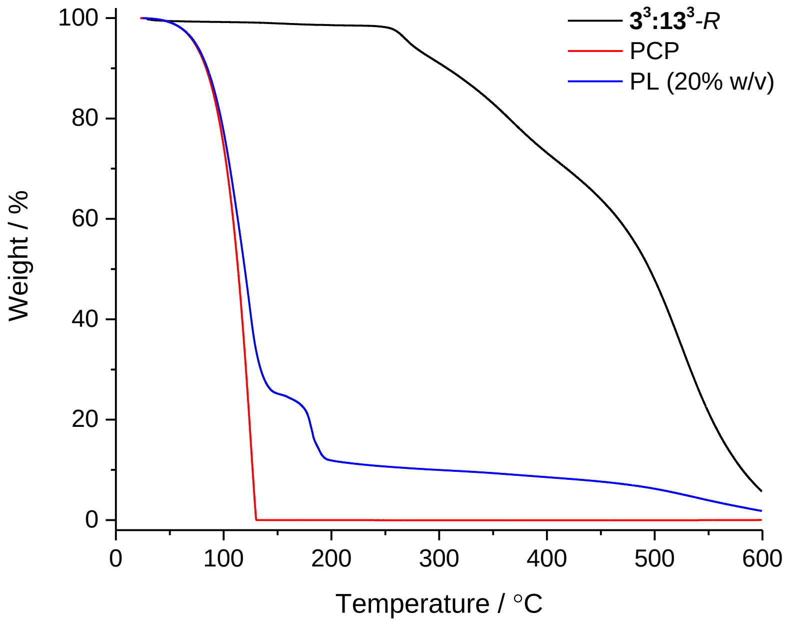

Supplementary Fig. 16: TGA Analysis was conducted at a ramp rate of 5 °C/min up to 600 °C in an aluminium pan under a nitrogen flow for the scrambled cage mixture (**3^3:13^3**-*R*, black), PCP (red) and the PL (20% w/v, blue).

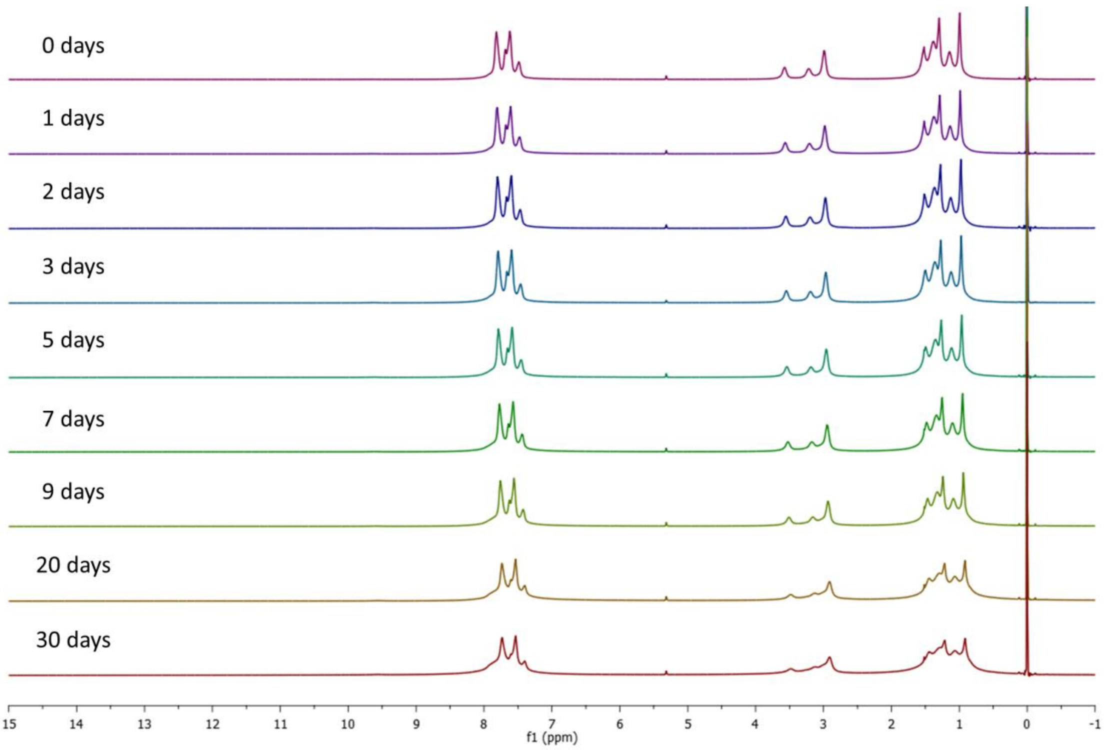

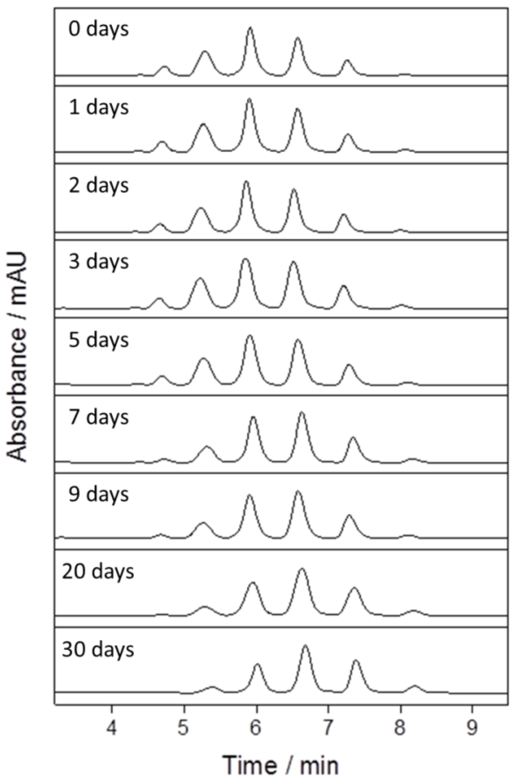

Supplementary Fig. 17: Chemical stability of the PL—Stacked ^{1}H NMR spectra (top) of the same sample of PL over 30 days shows slight peak broadening from 20 days which could be indicative of the beginning of cage catenation, or possibly the decomposition of PCP to afford HCl which then causes broadening of the cage signals, but the stacked HPLC traces (bottom) confirm the presence of the scrambled $\mathbf{3^3{:}13^3}$-*R* cage mixture even after 30 days.

3.5 Standard Procedure for Density Calculations

All measurements were repeated three times to calculate an average density with corresponding standard deviation.

PCP: To a pre-weighed oven dried 1 mL volumetric flask with lid was added 1 mL PCP (freeze-pump-thaw degassed purified material) and the weight recorded.

PL: Three batches of 20% w/v PL were made-up according to the standard procedure (200 mg in 1 mL) and 1 mL of each was added to oven dried 1 mL volumetric flasks and the weights recorded.

Supplementary Table 2: Calculation of the average densities of PCP and the PL (20% w/v) enabling the molar ratio of cage to solvent to be calculated, and for calculation of gas uptakes in later experiments (mmol/g).

**Sample**	**Temp (°C)**	**Sample 1 Mass (g)**	**Sample 2 Mass (g)**	**Sample 3 Mass (g)**	**Average Density (g/mL)**	**Standard Deviation (± g/mL)**
PCP	25	1.7112	1.7149	1.7121	1.7127	0.0019
PL (20 % w/v)	19	1.6193	1.6052	1.5974	1.6073	0.0111

Using the measured density of the purified PCP (*vide infra*) the molar ratio of scrambled $\mathbf{3^3{:}13^3}$-*R* cage to solvent in the 20% w/v PL can be calculated—1:35.8 cage:PCP (0.192:6.885 mmol) *i.e.* approximately one cage for every 36 solvent molecules (average scrambled cage MW = 1039.34 g/mol; PCP MW = 248.75 g/mol; measured density of pure PCP, ρ = 1.7127 g/mL).

The pore volume can also be calculated using the measured density of the PL (20% w/v). For a sample containing 200 mg $\mathbf{3^3{:}13^3}$-*R* in 1 mL PCP the total mass will be 1.9127 g, with an overall volume of 1.19 mL. In this sample there will be $1.92\text{x}10^{-4}$ mol cage, and therefore $1.159\text{x}10^{20}$ cage molecules. Assuming a cavity size of 5 Å, the pore volume in a single cage will be $6.545\text{x}10^{-23}$ mL, leading to a total pore volume of $7.586\text{x}10^{-3}$ mL. This leads to a percentage pore volume of 0.64%.

4. Computational model and molecular dynamics simulations

The Amorphous Cell module in Materials Studio[3] was used to generate a 74.44 Å amorphous box of scrambled **3^3:13^3**-*R*. In total, 30 cages were inserted and for simplicity one of the most prevalent isomers in the mixture (see **Supplementary Fig. 4**), a **3^3:13^3**-*R* isomer, was selected (see **Fig. 3a**)—this removes the difficulty of positional isomerism, and it is assumed this would not affect the results. DL_FIELD[4] was then used to solvate the system with PCP, such that the ratio of cage:PCP was 1:36. The system was then checked to see whether PCP was inserted in the cage cavity. This is important, as the movement of PCP with respect to the cage can be accurately monitored. **Supplementary Fig. 18** shows this process.

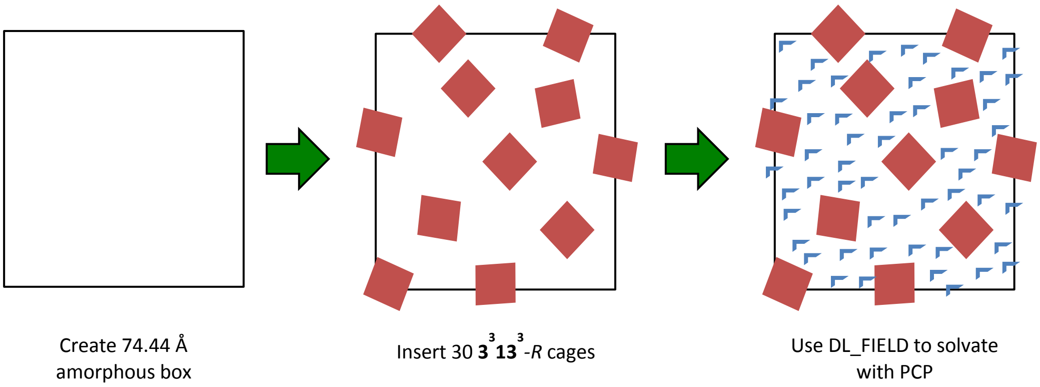

Supplementary Fig. 18: Scheme illustrating the setup of the PL.

MD simulations were subsequently carried out using DL_POLY_2.20.[5,6] A potential cut-off of 10 Å was used and electrostatic interactions were calculated using the partial charges from OPLS FF. An NVT ensemble (constant number of moles, volume and temperature) at 1 atm and 298 K was used with the Hoover barostat and thermostat,[7] and both had a time constant of 0.5 ps. A timestep of 0.5 fs was used, with the system first equilibrated for 50 ps with temperature scaling every 5 fs, followed by a production run of 10 ns, with a frame output every 2.5 ps.

Once complete, the system was analysed using an in-house script to determine the centre of mass for each cage and PCP molecule. Vectors between these were calculated for each frame of the MD simulation, such that it was possible to determine the distance between the centre of the cages, and each PCP. To ascertain whether a PCP molecule had in fact entered a cage, the following criteria were used:

Supplementary Table 3: Criteria used for determining where the PCP molecules were during the simulation, with respect to the cage centre.

**Location of PCP**	**Distance (Å)**	**Average Frequency (%)**
Inside the cage cavity	3.5	0
In the cage window	5.5	3.29
Near neighbour to the cage (essentially surrounding the cage)	9.0	16.53
Away from cage molecules	>9.0	80.18

It was also possible to monitor the simulation density, to make sure it agrees well with that observed experimentally. The average density of the system was maintained at 1.54 g/cm^3 post equilibration.

5. Spectroscopic Gas Uptake and Release Studies

5.1 FTIR Studies

5.1.1 Optimisation and Reproducibility of CO_2 Uptake

Sample Preparation: Batches of the PL (20% w/v) were made-up according to the standard procedure in either non-degassed or freeze-pump-thaw degassed pure PCP (200 mg in 1 mL). The PL then had CO_2 bubbled through at either 5–10, 50–60 or 200–220 mL/min (6–14, 44–49 or 95–100 respectively on Gilmont flowmeter scale with a stainless steel float) with an 18 gauge needle as an outlet for 2 h under a range of conditions including ice-cooled or at rt (17–25 °C), with or without the addition of 1.0 eq. water, sonicated prior to CO_2 addition, with pre-wet cage or using cage a day after desolvation, and with duplicates conducted and different batches of scrambled cage used to test reproducibility. Control samples were conducted on 1 mL PCP under the same conditions.

Supplementary Table 4: Calculation of the different corrected gas flows of CO_2 from air flow, and their corresponding Gilmont scale readings.

Gas	Density of CO_2 (g/mL) at standard conditions (1 atm, 294 K) from NIST WebBook	Gilmont Scale Reading (SS Float)	Calibrated Air Flow q_A° at standard conditions (mL/min)	Corrected Gas Flow q_G° from Air Flow q_A° at standard conditions (mL/min)
CO_2	$\rho_G^{\circ} = 0.00183$	6–14	6.314–11.76	5.11–9.52
		44–49	60.74–73.42	49.18–59.45
		95–100	248.4–271.8	201.14–220.09

IR analysis: IR analysis of CO_2 uptake conducted immediately after gas introduction, and after leaving the sample in ambient conditions overnight, with the neat liquid samples in a Specac omni-cell demountable liquid cell with calcium fluoride (CaF_2) plates and a 0.05 mm PTFE insert.

NB. It was noted that an accurate measure of the CO_2 content could not be achieved by using a drop of the liquid on an ATR module as this looks at the surface of the liquid, and not through it as in a liquid cell.

The absorbance IR spectra were integrated using Origin with the CO_2 signal integrated from 2300.9509–2368.4558 cm^{-1} and the PCP signal integrated from 1500.5363–1579.6134 cm^{-1}. All CO_2 uptakes were made relative to the PCP to allow for comparisons.

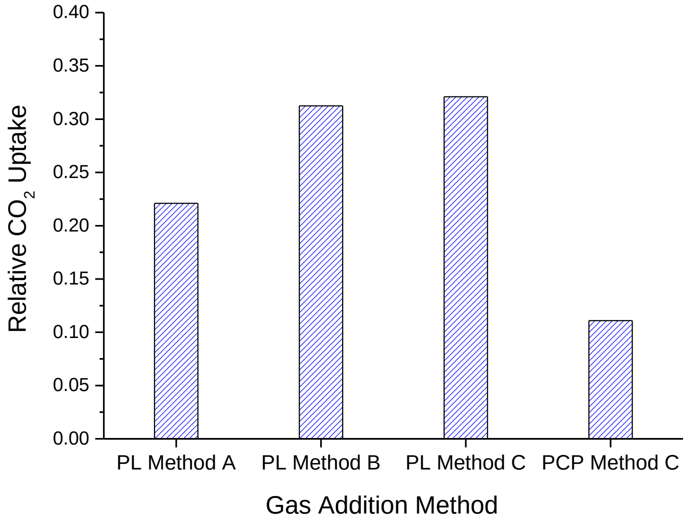

Supplementary Fig. 19: Comparison of gas addition method and its effect on the relative CO_2 uptake by FTIR (Method A = Gas flow over solid scrambled $\mathbf{3^3{:}13^3}$-*R* cage mixture (30 min) prior to dissolving in PCP; Method B = Gas flow over solid (30 min) followed by bubbling through PL solution (2 h); Method C = Gas bubbled through PL solution or PCP (2 h) demonstrating that bubbling the gas through the solution is the most efficient method of introducing CO_2.

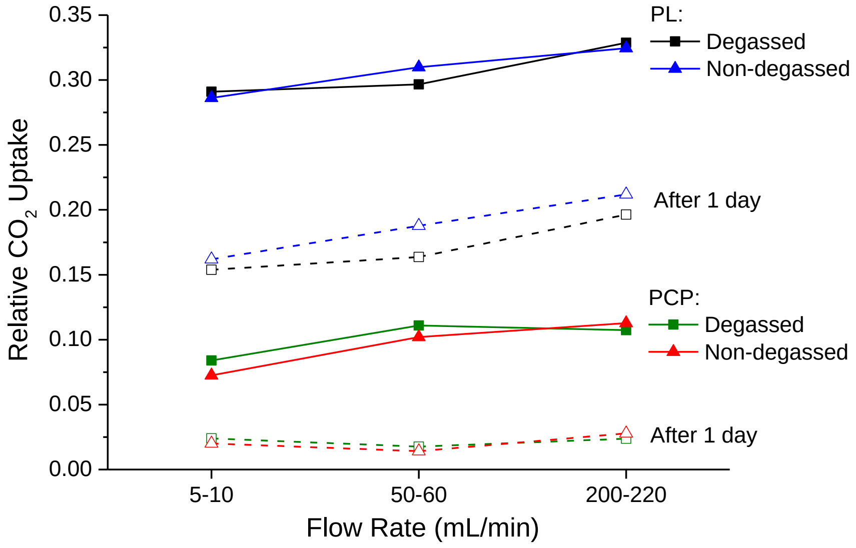

Supplementary Fig. 20: Effect of degassed *vs* non-degassed solvent on CO_2 uptake at a range of flow rates and the effect of cage presence on CO_2 retention by FTIR. Similar uptake occurs whether degassed or non-degassed PCP is used, with variation of the flow rate of gas having no major effect. The PL shows an ~3-fold increase in CO_2 uptake compared to PCP, and retains CO_2 after a day whereas the PCP does not.

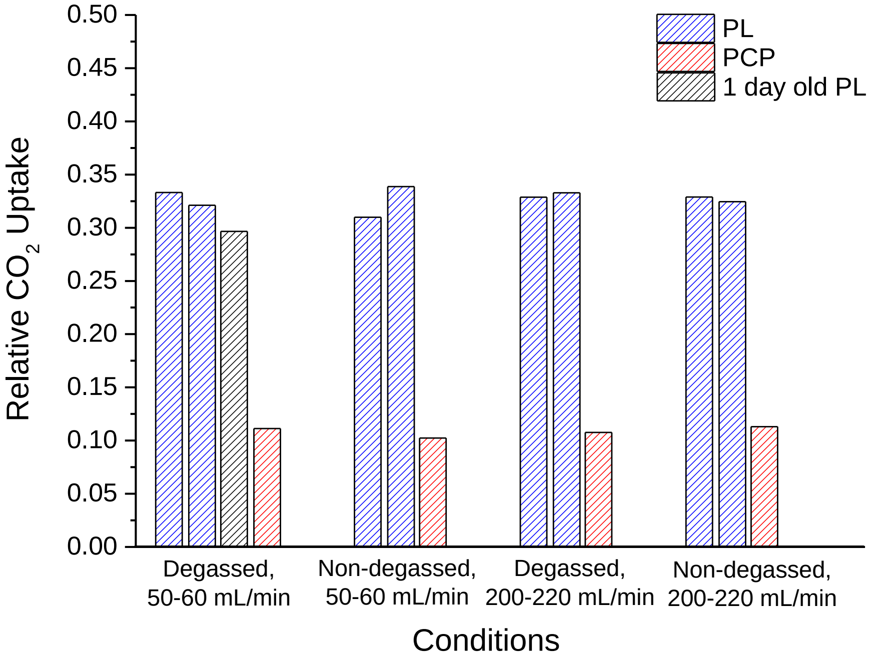

Supplementary Fig. 21: Reproducibility of CO_2 uptake between different methods by FTIR. Uptake reproducible between different samples, whether degassed or non-degassed PCP used, and with variation of the flow rate of gas.

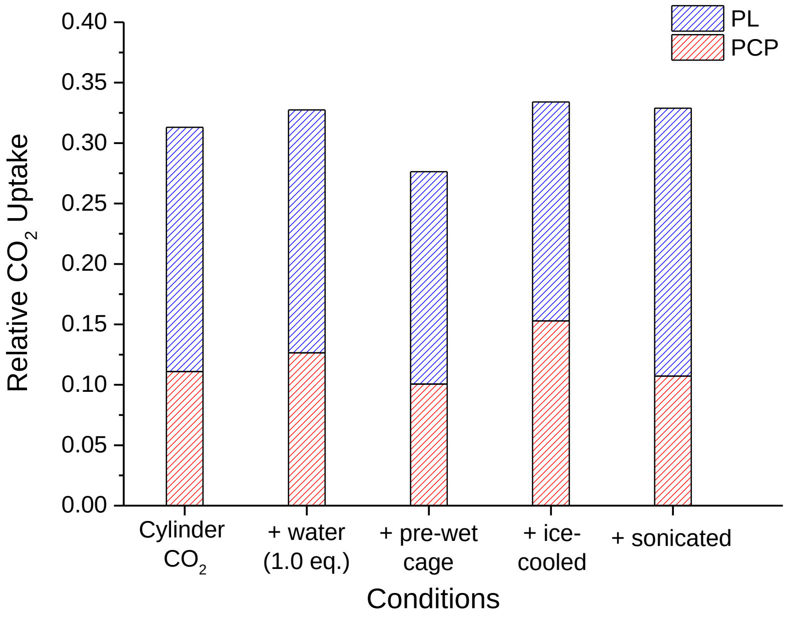

Supplementary Fig. 22: Effect of varying conditions on CO_2 uptake by FTIR (degassed PCP and 50–60 mL/min flow rate used). No appreciable difference in CO_2 uptake observed under a variety of conditions with only a reduction apparent if the cage was pre-treated with water.

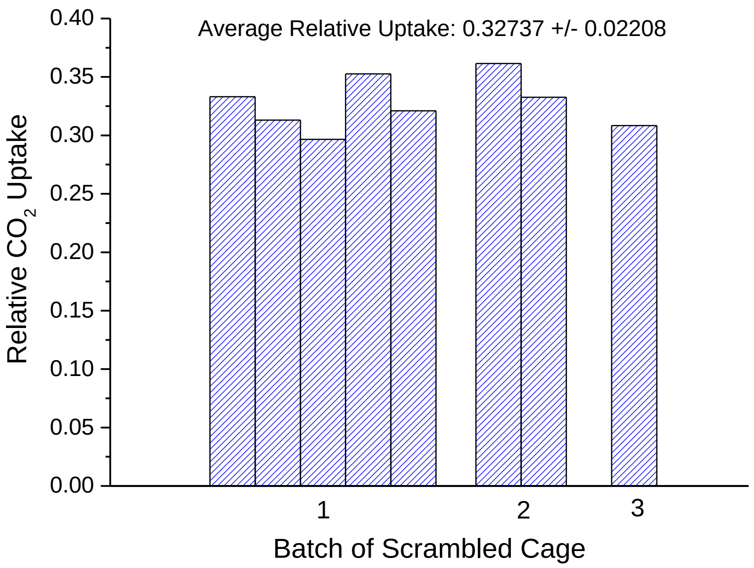

Supplementary Fig. 23: Reproducibility of CO_2 uptake in the PL (20% w/v) between batches by FTIR (degassed PCP and 50–60 mL/min flow rate used)—CO_2 uptake similar and reproducible between different PL samples formed using three different batches of scrambled **3^3:13^3**-*R* cage.

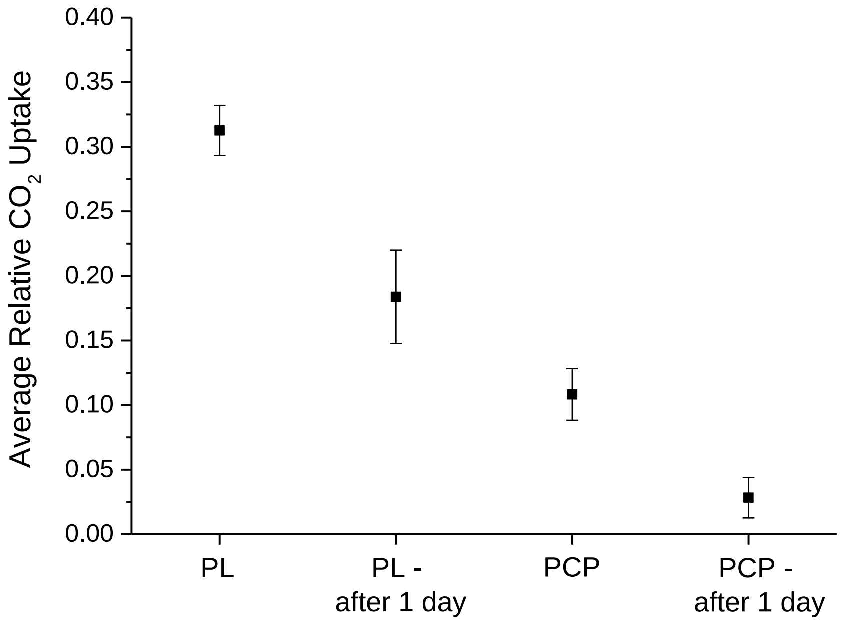

Supplementary Fig. 24: Average relative CO_2 uptake of the PL and PCP calculated from all attempted conditions shown as a mean with standard deviations. Overall, the PL's ability to uptake CO_2 is fairly reproducible, and always displays an ~3 fold increase compared to the neat PCP.

5.1.2 CO_2 Saturation Test

PCP: Six samples were prepared according to the standard procedure (1 mL) and CO_2 was bubbled through at a flow rate of ~50–60 mL/min (44–49 on Gilmont flowmeter scale with a stainless steel float) with an 18-gauge needle as an outlet for a set time period prior to IR analysis.

PL: Six batches of 20 % w/v PL were prepared according to the standard procedure (200 mg in 1 mL) and CO_2 was bubbled through at 50–60 mL/min (44-49 on Gilmont flowmeter scale with a stainless steel float) with an 18-gauge needle as an outlet for a set time period prior to IR analysis.

IR analysis: IR analysis of CO_2 uptake conducted immediately after gas introduction (0, 5, 10, 20, 30, 60 and 120 min) with the neat liquid samples in a Specac omni-cell demountable liquid cell with calcium fluoride (CaF_2) plates and a 0.05 mm PTFE insert.

The absorbance IR spectra were integrated using Origin with the CO_2 signal integrated from 2300.9509–2368.4558 cm^{-1} and the PCP signal integrated from 1500.5363–1579.6134 cm^{-1}. All CO_2 uptakes were made relative to the PCP to allow for comparisons.

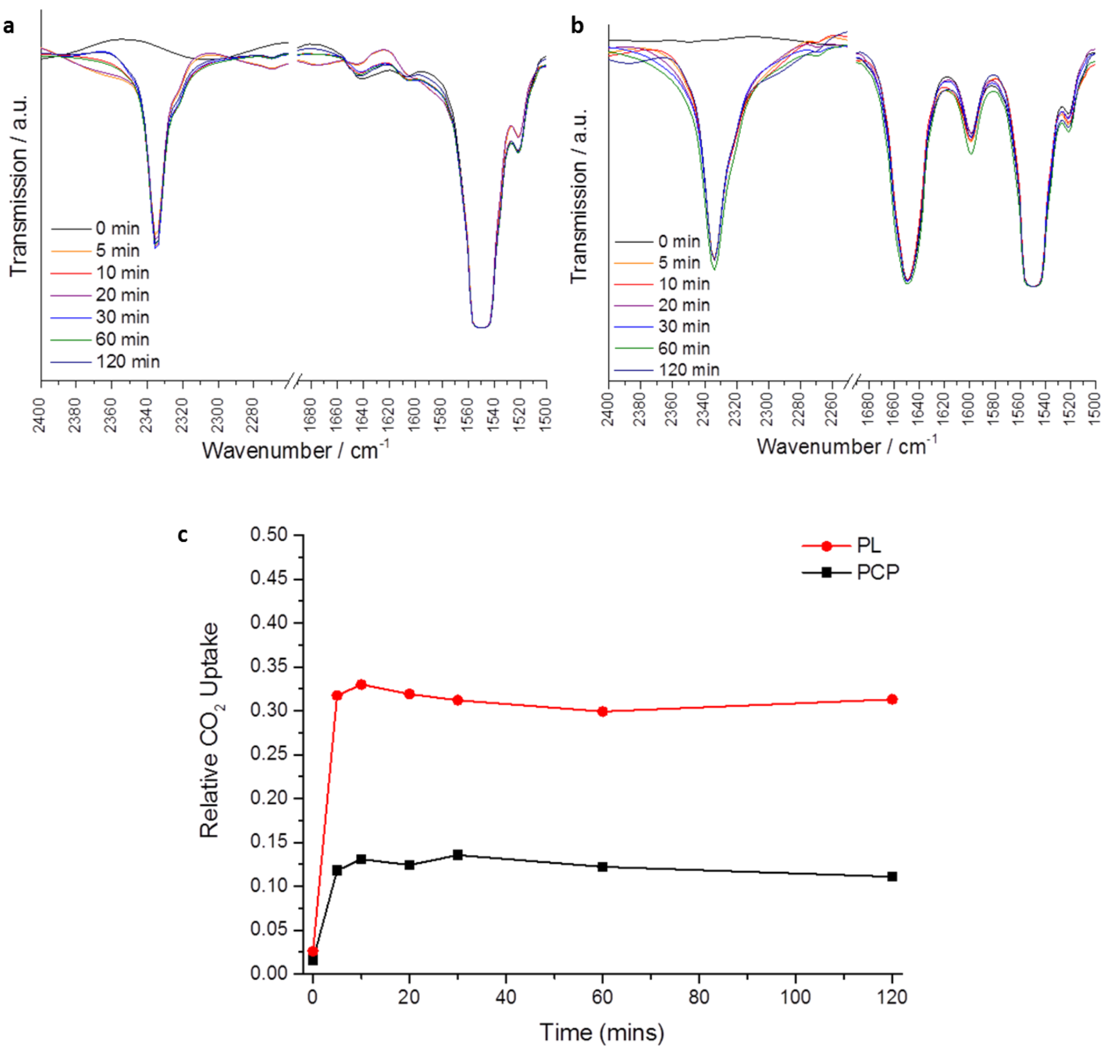

Supplementary Fig. 25: CO_2 Saturation test of PL and PCP by FTIR. (a) Overlaid FTIR spectra of PCP with CO_2 uptake over time. (b) Overlaid FTIR spectra of the PL with CO_2 uptake over time. (c) Saturation curve plotted showing relative CO_2 uptake over time for both PCP and the PL with both reaching saturation after as little as 5 minutes of CO_2 addition *via* bubbling at 50–60 mL/min.

Scalability Test: 200 mg Scrambled **3**[3]**:13**[3]-*R* cage in 1 mL PCP found to be saturated after 5 min at a flow rate of 50–60 mL/min, therefore the same experiment was subsequently conducted on 2 mL and 3 mL of the PL with CO_2 introduced for 10 min and 15 min respectively to check scalability.

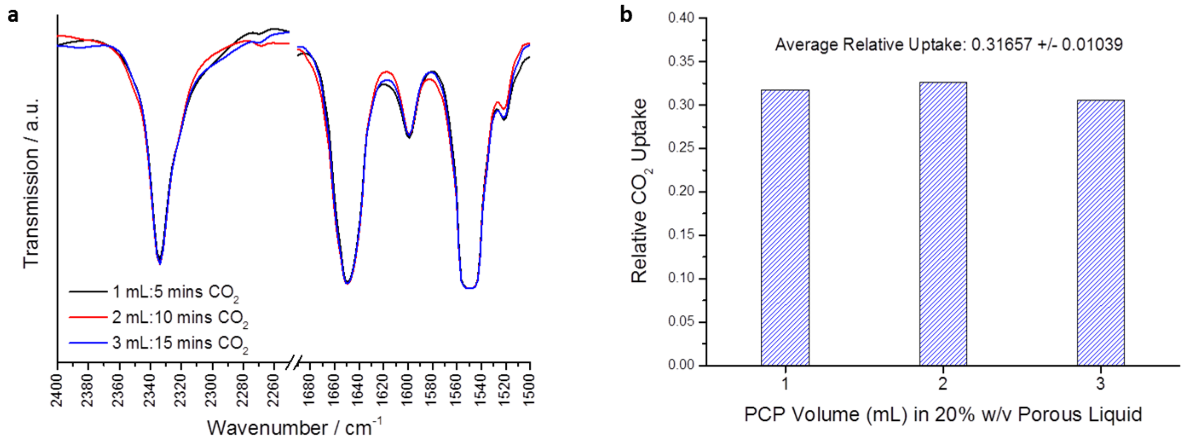

Supplementary Fig. 26: Saturation scale-up test measuring the PL CO_2 uptake at 50–60 mL/min for 5 min per 1 mL of PCP used. (a) Overlaid transmission IR of 1, 2, and 3 mL of the PL after CO_2 addition for 5, 10, and 15 min respectively. (b) Calculated relative CO_2 uptake for the different scales confirming that CO_2 can be reproducibly introduced for 5 min at 50–60 mL/min per 1 mL of PCP used in the PL.

5.1.3 Study of CO_2 Retention

PCP: Two samples were prepared according to the standard procedure (1 mL) in 4 mL oven-dried glass vials and CO_2 was bubbled through for 5 min at a flow rate of ~50–60 mL/min (44–49 on Gilmont flowmeter scale with a stainless steel float) with an 18-gauge needle as an outlet before analysis by FTIR to confirm saturation. The sample was then left in the capped vial and stored at either rt or at -20 °C in the freezer with daily analysis of the CO_2 content by FTIR.

PL: Two samples of 20% w/v PL were prepared according to the standard procedure (200 mg in 1 mL) and CO_2 was bubbled through for 5 min at 50–60 mL/min (44–49 on Gilmont flowmeter scale with a stainless steel float) with an 18-gauge needle as an outlet before analysis by FTIR to confirm saturation. The sample was then left in the capped vial and stored at either rt or at -20 °C in the freezer with daily analysis of the CO_2 content by FTIR.

IR analysis: IR analysis of CO_2 uptake conducted immediately after gas introduction and after 1, 2, and 3 days with the neat liquid samples in a Specac omni-cell demountable liquid cell with calcium fluoride (CaF_2) plates and a 0.05 mm PTFE insert.

The absorbance IR spectra were integrated using Origin with the CO_2 signal integrated from 2300.9509–2368.4558 cm^{-1} and the PCP signal integrated from 1500.5363–1579.6134 cm^{-1}. All CO_2 uptakes were made relative to the PCP to allow for comparisons.

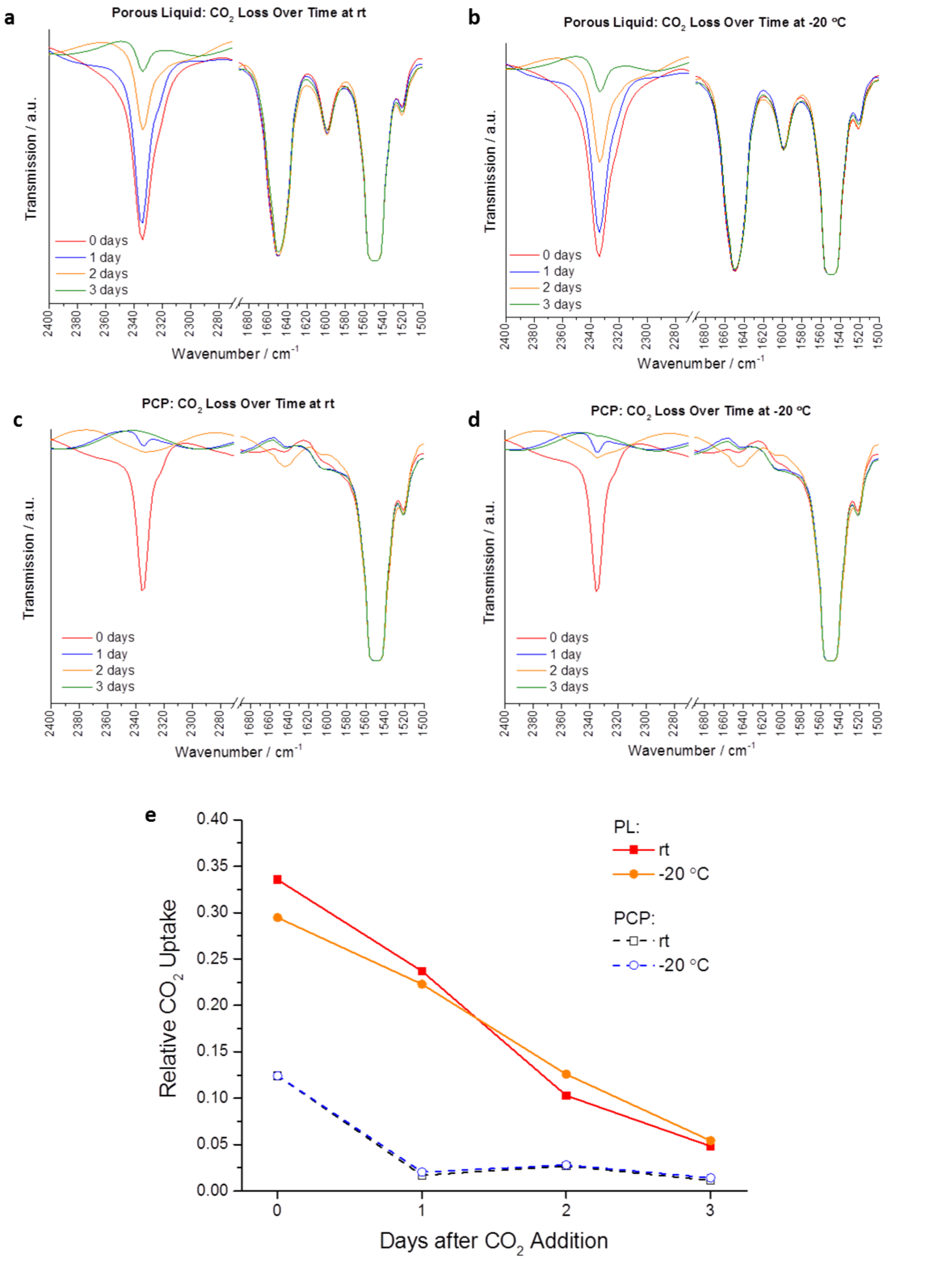

Supplementary Fig. 27: CO_2 Retention test of PL and PCP by FTIR at both rt and at -20 °C in the freezer. (a) Overlaid FTIR spectra of the PL with CO_2 loss at rt over 3 days. (b) Overlaid FTIR spectra of the PL with CO_2 loss at -20 °C in the freezer over 3 days. (c) Overlaid FTIR spectra of PCP with CO_2 loss at rt over 3 days. (d) Overlaid FTIR spectra of PCP with CO_2 loss at -20 °C in the freezer over 3 days. (e) Release curve plotted showing relative CO_2 content over 3 days with the PCP losing all of its CO_2 after 1 day and the PL only retaining it for 3 days.

5.2 NMR Studies

5.2.1 CH_4 Uptake by ^1H NMR

All samples (0.6 mL) had a ^1H NMR spectra recorded using the same sealed d_2-DCM/TMS capillary as an internal standard (capillary 1), both prior to CH_4 addition and after (3 min bubbling at 50–60 mL/min, 32–36 on Gilmont flowmeter scale with a stainless steel float). *N.B*. As a precaution, an NMR lid with a hole in was used to avoid build-up of pressure due to gas release.

PCP: 0.6 mL freeze-pump-thaw degassed pure PCP.

PL: Conducted on 20% w/v PL (120 mg dissolved in 0.6 mL) prepared according to the standard procedure.

Supplementary Table 5: Duplicates of ^1H NMR uptake of CH_4 in PCP and PL showing reproducibility in peak shift and integration.

Sample		CH_4 Shift (ppm)	CH_4 Integration (4 H) relative to TMS = 1 (12 H, 0.016 to -0.016 ppm)
PCP	1	-0.24	0.11
	2	-0.24	0.12
PL (20% w/v)	1	-2.80	0.83
	2	-2.75	0.81

Calibration of d$_2$-DCM/TMS Sealed Capillaries with PL at Varying Concentrations: To small vials was added either 25, 50, 100, 150, 175, or 200 mg of desolvated scrambled **3^3:13^3**-*R* cage (vacuum oven, 90 °C overnight) followed by 0.8 mL freeze-pump-thaw degassed pure PCP. Once the solid was fully dissolved (vortexed) the solution was transferred to 1 mL volumetric flasks. The sample vials were rinsed with a 0.1 mL PCP and the solution added to the volumetric flasks before making the sample up to 1 mL with further solvent. Each sample then had 0.6 mL taken for ^{1}H NMR analysis using two individual sealed d$_2$-DCM/TMS capillaries (washed and dried between samples)—capillary 1 and 2.

For each spectrum the TMS signal (0 ppm) was integrated to 1 (12 H, 0.016 to -0.016 ppm) and the NC*H* cage peaks integrated relative to it (12 H, 3.819–2.682 ppm) allowing calibration curves to be plotted over varying concentrations of the PL to enable accurate calculation of concentrations of samples used in NMR studies.

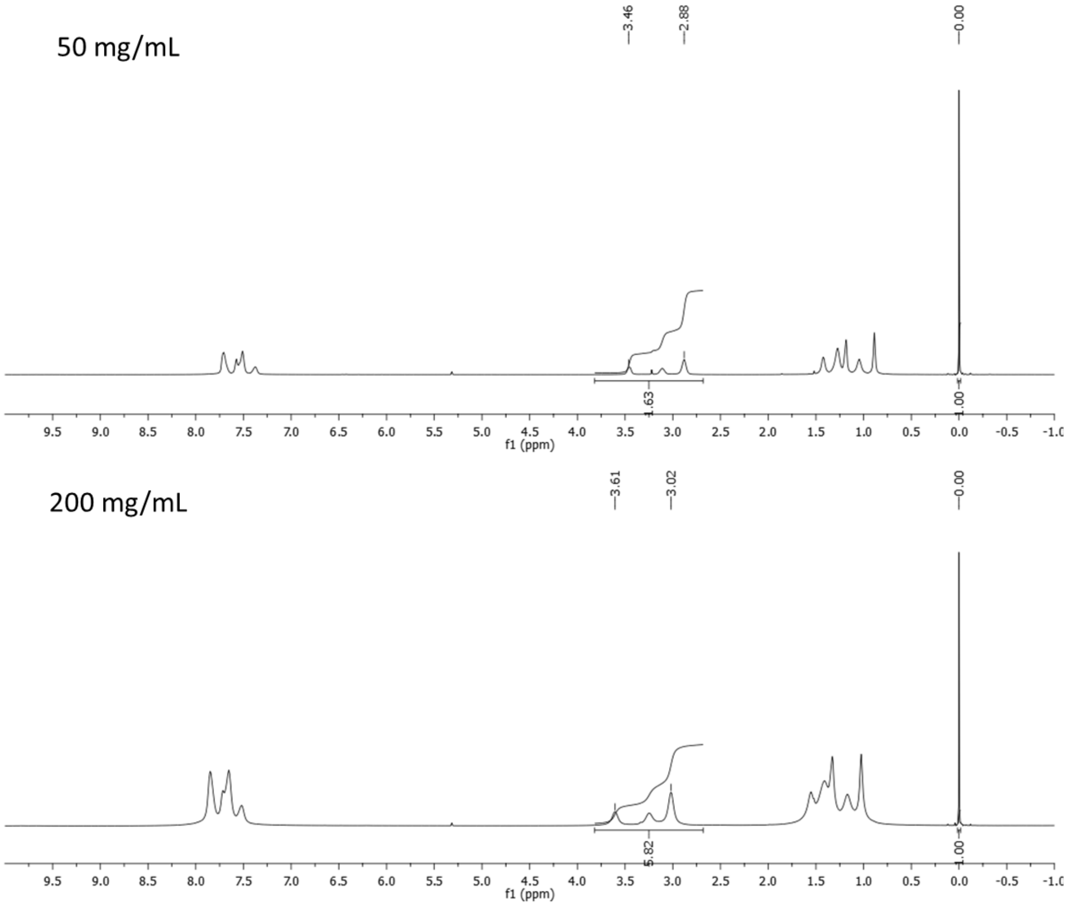

Supplementary Fig. 28: Example ^{1}H NMR spectra of the PL at concentrations of 50 and 200 mg/mL with d$_2$-DCM/TMS capillary 1, and the integrated peaks used to generate the calibration curve.

Supplementary Table 6: Integrated NC*H* peak relative to TMS for a range of PL concentrations with both capillary 1 and 2.

**PL Concentration (mg/mL)**	**NC*H* Integration** (12 H, 3.819-2.682 ppm) relative to TMS = 1 (12 H, 0.016 to -0.016 ppm)	
	**Capillary 1**	**Capillary 2**
25	0.88	1.69
50	1.63	3.24
100	3.05	6.01
150	4.21	9.08
175	4.95	10.56
200	5.82	11.94

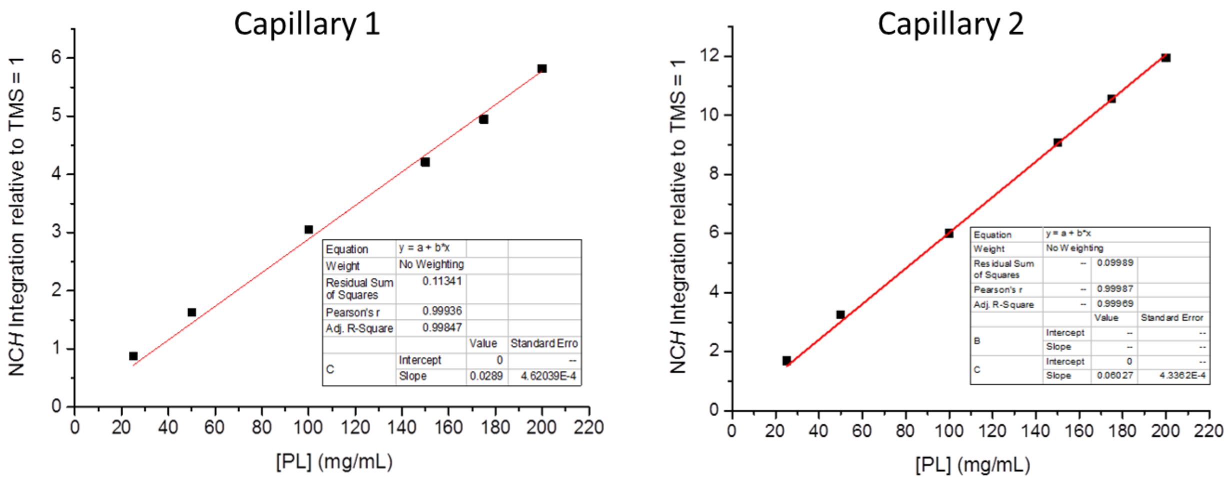

Supplementary Fig. 29: Calibration curves generated for each d_2-DCM/TMS sealed capillary by plotting the integration of the NC*H* cage peak relative to TMS = 1 from the ^{1}H NMR spectra, against the concentration of the PL.

Therefore, calculation of the PL concentration using the calibration curve is possible using $y = a + b{*}x$ so for each capillary the equation is:

Capillary 1	Capillary 2
$y = 0.0289x$	$y = 0.06027x$
$x = \frac{y}{0.0289}$	$x = \frac{y}{0.06027}$

Supplementary Table 7: Integration of the NC*H* cage peak (relative to TMS = 1 using the same capillary) from the 20% w/v PL samples used in the ^{1}H NMR analysis of CH_4 uptake (see **Supplementary Table 5**) allows an accurate calculation of the concentration of the PL (mg_{cage}/mL) to be conducted, which can be subsequently converted to $mmol_{cage}$/mL using the scrambled **3^3:13^3**-*R* cage mixtures average molecular weight.

	Capillary	**NC*H* Integration** (12 H, 3.819–2.682 ppm) relative to TMS = 1 (12 H, 0.016 to -0.016 ppm)	**Calculated [PL] (mg_{cage}/mL)** from calibration curve	**Conversion to $mmol_{cage}$/mL** (Av. MW = 1039.34)
1	1	4.43	153.3	0.1475
2	1	4.36	150.8	0.1451
3	2	9.61	159.4	0.1533

This allows a quantitative CH_4 uptake in the PL and PCP to be calculated.

Supplementary Table 8: Integration of the CH_4 peak relative to the cage NC*H* peak = 12 allows a ratio of cage : CH_4 to be determined, which in turn can be used to calculate the mmol/mL ratio by using the $mmol_{cage}$/mL calculated from the calibration curve (see **Supplementary Table 7**). The CH_4 uptake can then be converted to either mmol/g_{cage} using the calculated concentration (e.g. 153.4, 150.8, and 159.4 mg/mL, see **Supplementary Table 7**) allowing comparison to other sorption on solid cages, or to mmol/g_{PL} using the measured density of the 20% w/v PL (see **Supplementary Table 2**) allowing comparison to uptake in alternative porous liquids.

	Capillary	**Cage NC*H* Integration set to 12 H** (3.819–2.682 ppm)	**CH_4 Integration relative to cage NC*H*** (4 H, -2.762 to -2.862 ppm)	**Cage : CH_4 ratio from Integrations**	**Conversion to mmol/mL Ratio**	**CH_4 Uptake (mmol/g_{cage})**	**CH_4 Uptake (mmol/g_{PL})** ρ = 1.6073 g/mL
1	1	12	2.25	1 : 0.5625	0.1475 : 0.0829	0.5407	0.0515
2	1	12	2.24	1 : 0.5600	0.1451 : 0.0812	0.5388	0.0505
3	2	12	2.26	1 : 0.565	0.1533 : 0.0866	0.5432	0.0538

Supplementary Table 9: Calculation of CH_4 uptake in PCP from the ^{1}H NMR spectra using the same calibrated capillary and measured densities (see **Supplementary Table 2**) enabling a quantitative uptake in mmol CH_4/g to be determined.

	**Capillary**	**Average CH_4 Uptake from ^{1}H NMR** (mmol/mL)	**Average CH_4 Integration** (4 H) relative to TMS = 1 (12 H, 0.016 to -0.016 ppm)	**Average Calculated CH_4 Uptake using ratio of integrations** (mmol/mL)	**CH_4 Uptake (mmol CH_4/g)**
PL (20% w/v)	1	0.0820	0.82	0.0820	0.0510 mmol/g_{PL} ρ = 1.6073 g/mL
PCP	1	-	0.115	0.0115	0.0067 mmol/g_{PCP} ρ = 1.7127 g/mL

Supplementary Table 10: Comparison of the CH_4 uptake and cage content in the previously reported crown-ether porous liquid[8] with the scrambled porous liquid. Whilst the liquids have comparable CH_4 uptake in terms of µmol/g_{PL}, the molar amount of cage present in the scrambled porous liquid is approximately half of that in the crown-ether porous liquid.

**Crown-ether PL**		**Scrambled PL**	
**Cage MW**	2102.45	**Cage Average MW**	1039.34
**15-crown-5 MW**	220.27	**PCP MW**	248.75
**PL Average MW (1:12)**	365.05	**PL Average MW (1:36)**	270.11
**1 g PL (mmol)**	2.74	**1g PL (mmol)**	3.70
**Amount cage in 1 g PL (mmol)**	0.21	**Amount cage in 1 g PL (mmol)**	0.10
**Mass cage in 1 g PL (mg)**	441	**Mass cage in 1 g PL (mg)**	103
**CH_4 Uptake (µmol/g_{PL})**	52	**CH_4 Uptake (µmol/g_{PL})**	51
**CH_4 Uptake (µmol/g_{cage})**	117.9	**CH_4 Uptake (µmol/g_{cage})**	495.1
**CH_4 Uptake (equiv.)**	0.24	**CH_4 Uptake (equiv.)**	0.51

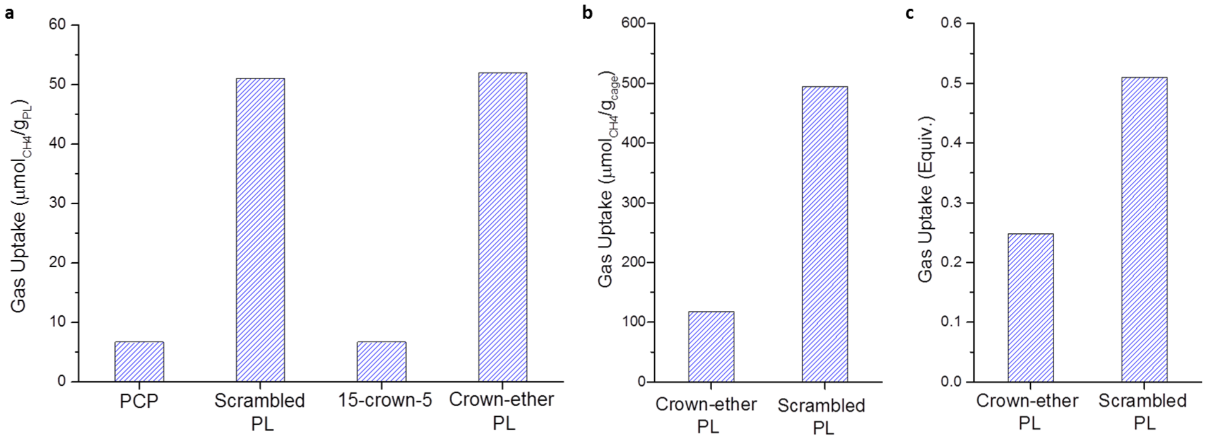

Supplementary Fig. 30: (a) Comparable CH_4 uptake in terms of µmol/g_{PL} is observed for both the previously reported crown-ether and the scrambled porous liquid. (b) The CH_4 uptake in terms of µmol/g_{cage} is approximately 4 times higher with the scrambled cage porous liquid compared to the crown-ether porous liquid. (c) There is also double the equivalents of CH_4 per cage in the scrambled porous liquid.

5.2.2 CH_4 Saturation Test

Both the PL (20% w/v—120 mg dissolved in 0.6 mL, prepared according to the standard procedure) and PCP (0.6 mL freeze-pump-thaw degassed) had a ^1H NMR spectra recorded using the same calibrated d_2-DCM/TMS capillary as an internal standard (capillary 1), both prior to CH_4 addition and after each set addition (3 or 9 min bubbling at 50–60 mL/min, 32–36 on Gilmont flowmeter scale with a stainless steel float).

NB. As a precaution, an NMR lid with a hole in was used to avoid build-up of pressure due to gas release.

Analysis of all the ^1H NMR spectra using the same method as shown in **Supplementary Tables 7-9** enabled a saturation curve of CH_4 uptake over time graph to be plotted for both PCP and the PL.

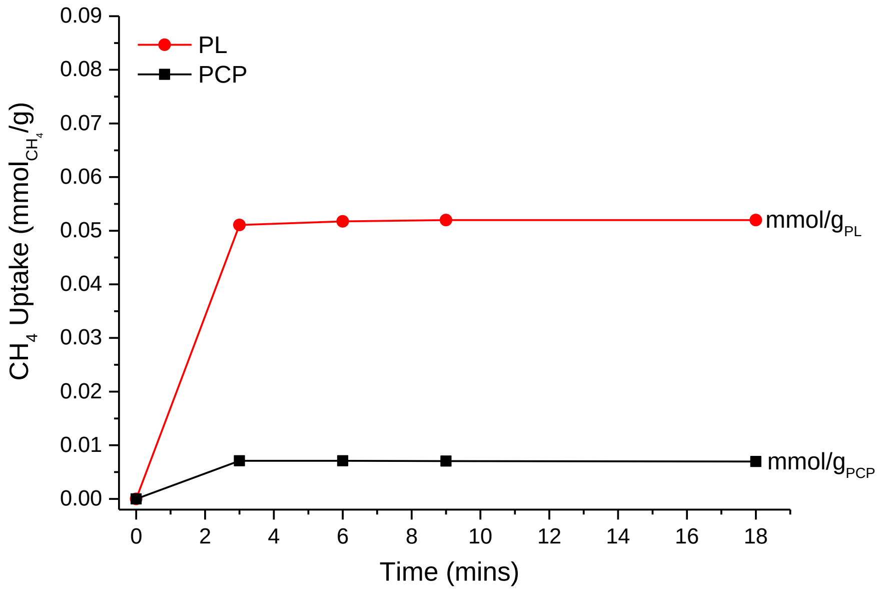

Supplementary Fig. 31: CH_4 Saturation test of PL (20% w/v, red) and PCP (black) using ^1H NMR analysis. Saturation curve plotted showing CH_4 uptake over time for both PCP and the PL, with both reaching saturation after 5 minutes of CH_4 addition per 1 mL of PCP used *via* bubbling at 50–60 mL/min. Mean at saturation: PCP = 0.0071±0.000063 mmol/g_{PCP}, PL = 0.0517±0.00043 mmol/g_{PL}.

5.2.3 Study of CH_4 Retention

The saturated PL and PCP samples from the CH_4 saturation study in **Supplementary Fig. 31** were left at rt in the NMR tubes, capped with a lid with a hole in to avoid build-up of pressure due to any gas release, and analysed by ^{1}H NMR spectroscopy after 1, 2, 5, and 7 days using the same calibrated d_2-DCM/TMS capillary as an internal standard (capillary 1).

Analysis of all the ^{1}H NMR spectra using the same method as shown in **Supplementary Tables 7-9** enabled the CH_4 content to be plotted for both PCP and the PL over time.

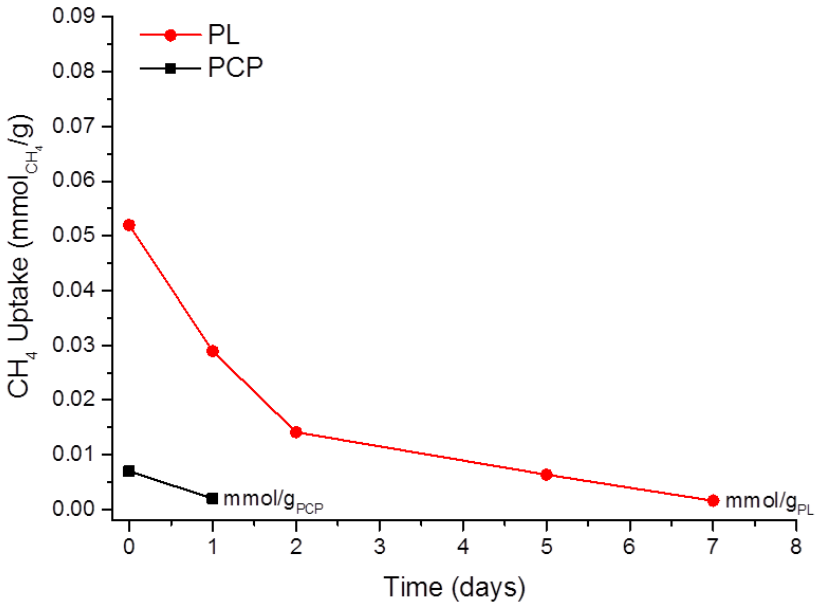

Supplementary Fig. 32: Study of CH_4 retention in the PL (20% w/v, red) and PCP (black) at rt by ^{1}H NMR analysis. Release curve plotted showing CH_4 content, with the PCP losing all of its CH_4 after 1 day and the PL retaining it for 7 days.

5.3 FTIR and NMR Studies of Control Systems

5.3.1 CO_2 Uptake

Control Non-PL: A sample of 20% w/v control non-PL was prepared according to the standard procedure (200 mg in 1 mL) and CO_2 was bubbled through for 5 min at 50–60 mL/min (44–49 on Gilmont flowmeter scale with a stainless steel float) with an 18-gauge needle as an outlet before analysis by FTIR.

FTIR analysis and calculation of relative CO_2 uptake conducted using the same procedure as for the PL and PCP (see section 5.1).

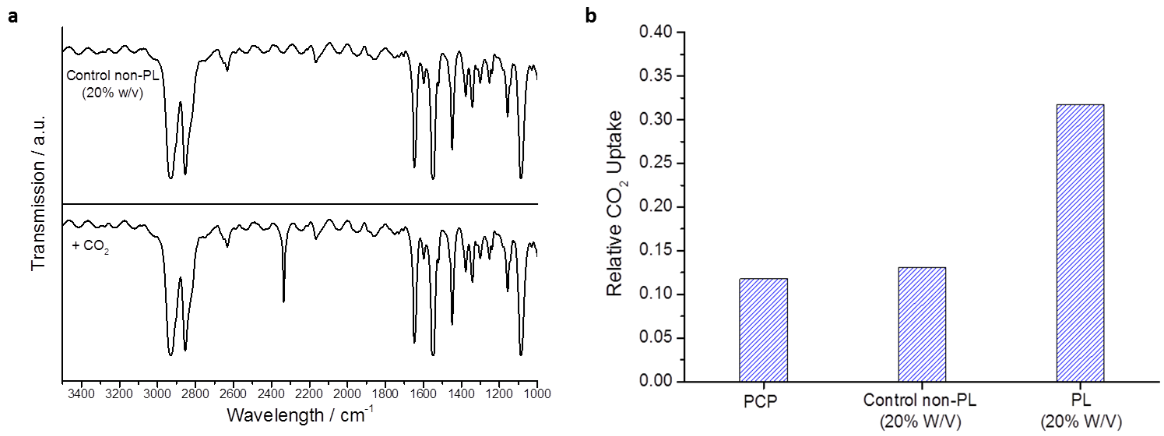

Supplementary Fig. 33: Comparison of CO_2 uptake in the PL (20% w/v), PCP and the control non-PL (20% w/v). (a) Stacked FTIR of the control non-PL both before and after CO_2 addition. (b) Comparison of the calculated relative CO_2 uptake demonstrating no enhanced solubility in the control liquid with the uptake being similar to the base solubility in neat PCP.

5.3.2 CH_4 Uptake

All samples (0.6 mL) had a ^1H NMR spectra recorded using the same calibrated d_2-DCM/TMS capillary as an internal standard (capillary 1), both prior to CH_4 addition and after (3 min bubbling at 50–60 mL/min, 32–36 on Gilmont flowmeter scale with a stainless steel float). *N.B.* As a precaution, an NMR lid with a hole in was used to avoid build-up of pressure due to gas release.

PCP: 0.6 mL freeze-pump-thaw degassed pure PCP.

PL: Conducted on 20% w/v PL (120 mg dissolved in 0.6 mL) prepared according to the standard procedure.

Control Non-PL: Conducted on 20% w/v control non-PL (120 mg dissolved in 0.6 mL) prepared according to the standard procedure.

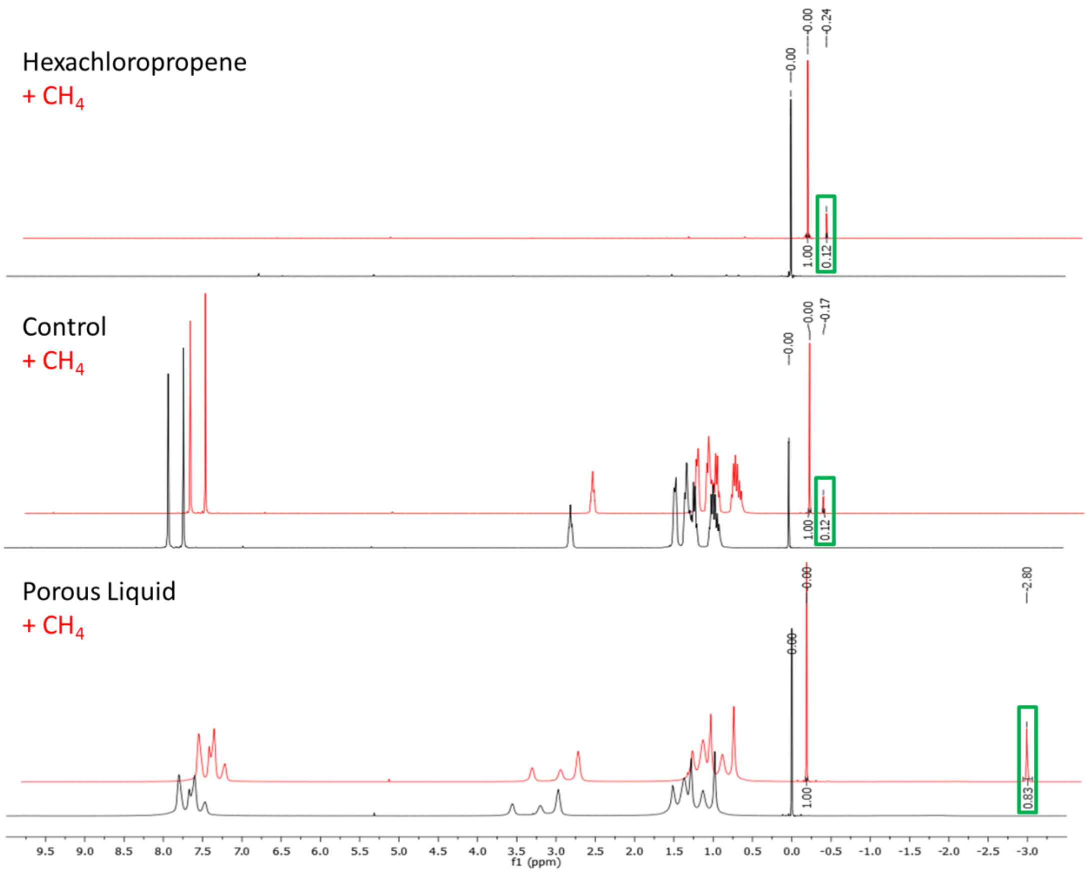

Supplementary Fig. 34: Stacked and overlaid ^1H NMR spectra of PCP (upper), the PL (20% w/v, lower) and the control non-PL (20% w/v, middle), both before (black) and after (red) CH_4 addition (green box). Demonstrates an ~7-fold increase in CH_4 uptake in the PL compared to PCP and a shielding effect of the CH_4 signal from -0.24 ppm to -2.80 ppm suggesting it's presence inside the cage cavity on the NMR time scale. No increased uptake or shielding of CH_4 was observed in the control non-PL compared to PCP.

A sample of both the **PL** and the **reduced-scrambled liquid** (0.6 mL, 20% w/v) were prepared according to the standard procedures and had ^1H NMR spectra recorded after CH_4 addition (3 min bubbling at 50–60 mL/min, 32–36 on Gilmont flowmeter scale with a stainless steel float) using the same calibrated d_2-DCM/TMS capillary as an internal standard (capillary 2). *N.B.* As a precaution, an NMR lid with a hole in was used to avoid build-up of pressure due to gas release.

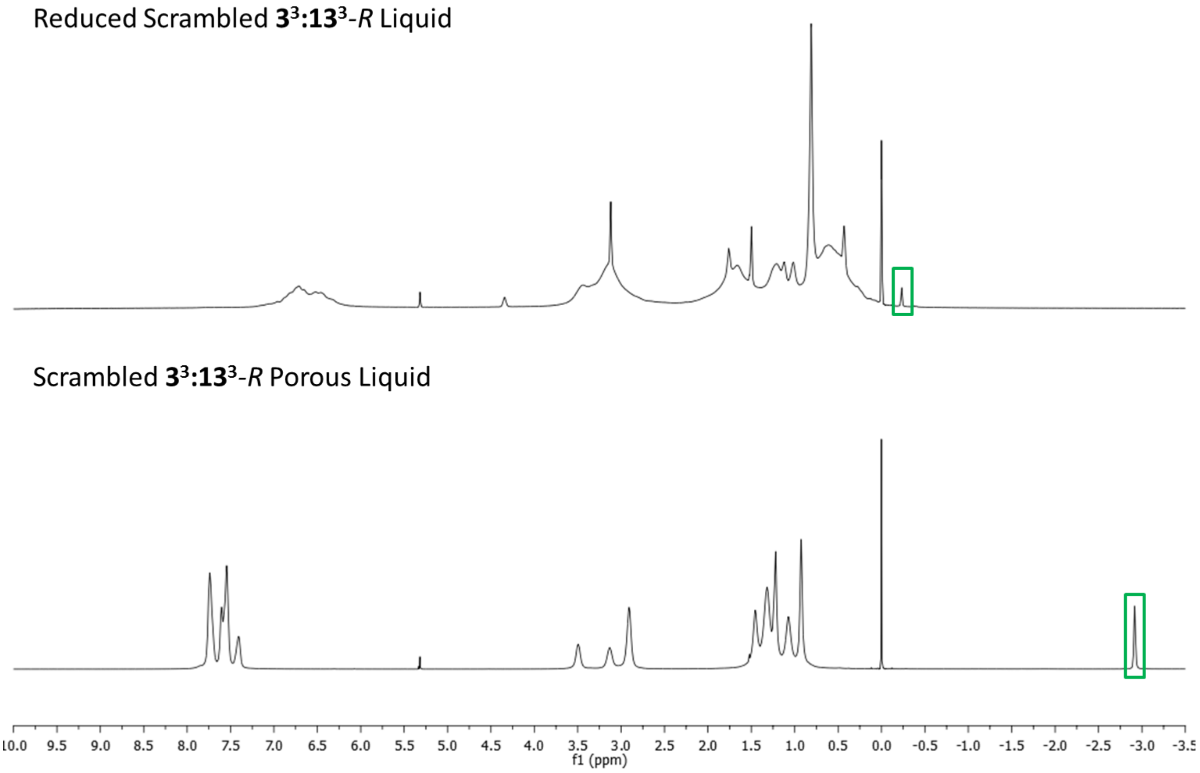

Supplementary Fig. 35: Stacked ^1H NMR spectra of the reduced-scrambled liquid (20% w/v, top) and the scrambled PL (20% w/v, bottom) after CH_4 addition (green box). No increased uptake or shielding of CH_4 was observed over that observed in PCP (see **Supplementary Fig. 34**) showing that in order for an increased gas uptake to be observed, shape-persistent cages are required over flexible ones.

Supplementary Table 11: Comparison of the CH_4 ^1H NMR shifts shows no increased shielding effect in the control non-PL (20% w/v) or the reduced-scrambled liquid (Red-PL, 20% w/v) compared to PCP.

Sample	Capillary	CH_4 Shift (ppm)
PCP	1	-0.24
PL (20% w/v)	1	-2.80
	2	-2.91
Control Non-PL (20% w/v)	1	-0.17
Red-PL (20% w/v)	2	-0.23

Supplementary Table 12: Calculation of CH_4 uptake in the control non-PL (20% w/v) and the reduced-scrambled liquid (Red-PL, 20% w/v) from the ^1H NMR spectra in **Supplementary Figures 34-35** using the calibrated d_2-DCM/TMS capillaries, enabling a quantitative uptake in mmol/mL to be determined (see **Fig. 4f**).

	**Capillary**	**Average CH_4 Uptake from ^1H NMR** (mmol/mL)	**CH_4 Integration** (4 H) relative to TMS = 1 (12 H, 0.016 to -0.016 ppm)	**Calculated CH_4 Uptake using ratio of integrations** (mmol/mL)
PL (20% w/v)	1	0.0820	0.82	0.0820
PCP	1	-	0.115	0.0115
Control Non-PL (20% w/v)	1	-	0.12	0.0120
PL (20 % w/v)	2	0.0866	1.82	0.0866
Red-PL (20% w/v)	2	-	0.22	0.0104

5.3.3 Xe and SF_6 Uptake

All samples (0.6 mL) had either a ^{129}Xe or ^{19}F NMR spectra recorded, using the same calibrated d_2-DCM/TMS capillary as an internal lock, after 3 min bubbling of either Xe or SF_6 gas at 50–60 mL/min (60–66 or 62–69 respectively on Gilmont flowmeter scale with a stainless steel float). *N.B.* As a precaution, an NMR lid with a hole in was used to avoid build-up of pressure due to gas release.

PCP: 0.6 mL freeze-pump-thaw degassed pure PCP.

PL: Conducted on 20% w/v PL (120 mg dissolved in 0.6 mL) prepared according to the standard procedure.

Control Non-PL: Conducted on 20% w/v control non-PL (120 mg dissolved in 0.6 mL) prepared according to the standard procedure.

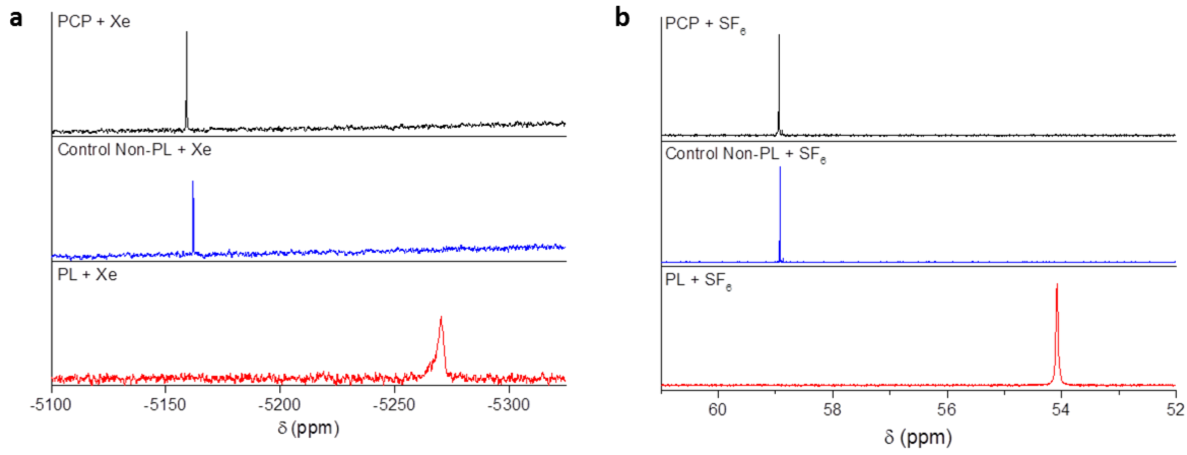

Supplementary Fig. 36: Stacked ^{129}Xe NMR (a) and ^{19}F NMR (b) spectra showing both xenon and sulfur hexafluoride uptake respectively in PCP (black), the control non-PL (20% w/v, blue) and the PL (20% w/v, red). Strong shielding effects are observed in the PL, indicative that Xe and SF_6 are present in the cage cavity on the NMR time scale but no shift is observed in the control non-PL.

5.4 Sonication as a Gas Release Mechanism

Sample Preparation: Two samples of the PL (20% w/v) were made-up according to the standard procedure (600 mg in 3 mL, and 400 mg in 2 mL) and subjected to cycles of CO_2 addition (bubbled through for 15 and 10 min respectively at 50–60 mL/min, 44–49 on Gilmont flowmeter scale with a stainless steel float) and release by sonication (30 min sonication per cycle, not heated). The 3 mL sample was connected to the gas collection setup during sonication and the uptake-release cycle repeated 5 times with the amount of evolved gas being measured each time, whereas the 2 mL sample was used for IR analysis.

IR analysis: IR analysis of CO_2 uptake conducted immediately after each gas addition and after each sonication, over 3 uptake-release cycles, with the neat liquid samples in a Specac omni-cell demountable liquid cell with calcium fluoride (CaF_2) plates and a 0.05 mm PTFE insert. The absorbance IR spectra were integrated using Origin with the relative quantity of CO_2 calculated as discussed previously (see **Fig. 6b and 6d**).

Controls: Two samples of PCP (3 mL) were prepared according to the standard procedure, and one had CO_2 added (bubbled through for 15 min at 50–60 mL/min, 44–49 on Gilmont flowmeter scale with a stainless steel float) prior to both undergoing release by sonication (30 min sonication per cycle, not heated) whilst connected to the gas collection setup.

Supplementary Table 13: Volumes of gas evolved during sonication over 5 cycles of CO_2 addition and release for the PL (20% w/v) remains constant, with a 3-fold increase in the amount evolved compared to a CO_2-saturated sample of PCP, and a 14-fold increase compared to PCP alone (see **Fig. 6c**).

Sample		Gas Evolution (cm^3)
PL (20% w/v) + CO_2	Cycle 1	4.0
	Cycle 2	4.2
	Cycle 3	4.3
	Cycle 4	4.5
	Cycle 5	4.6
PCP		0.3
PCP + CO_2		1.4

6. Guest Selectivity

6.1 Shape- and Size-Selectivity

6.1.1 Size-exclusion

Two samples of PL (200 mg in 1 mL) were prepared in vials according to the standard procedure and had Xe gas added (5 min at 50–60 mL/min, 60–66 on Gilmont flowmeter scale with a stainless steel float) followed by a stirrer bar. To one sample was carefully added chloroform (16 µL, 1.0 eq. relative to cage) and to the other 1-*t*-butyl-3,5-dimethylbenzene (36 µL, 1.0 eq. relative to cage). Both samples were then stirred and the gas release recorded – for video demonstrating guest selectivity in the PL see supplementary information for *Nature*, 2015, **527**, 216. Gas release was observed with the small guest as it can fit inside the cage cavity displacing the xenon, whereas the bulky guest cannot, thus demonstrating guest selectivity.

6.1.2 Gas Evolution Measurements

This guest selectivity of the PL allows gas evolution measurements to be conducted allowing estimated gas uptakes to be calculated without resorting to techniques such as FTIR or NMR.

Standard procedure for gas evolution studies of the porous liquid *via* small solvent release:

The desired amount of scrambled cage (typically 600 mg, 0.5772 mmol) was added to a pre-weighed GC headspace vial (22 mm x 45 mm screw top, 10 mL, Fisher Scientific) equipped with a stirrer bar and lid. The lid was kept separate while the vial containing the scrambled cage was desolvated in the vacuum oven at 90 °C overnight. On removing the samples from the oven the vials were immediately capped and reweighed to calculate the amount of desolvated scrambled cage present. The vial was connected to a manifold *via* a needle through the septum and evacuated for 10 min before N_2 was introduced prior to dissolving in the degassed PCP at a 20% w/v concentration (typically 3 mL). Once the sample was fully dissolved the desired gas was bubbled through the solution at a rate of 50–60 mL/min for 5 min per 1 mL of PCP used in the PL (39–43, 32–36, 44–49, 60–66 or 62–69 for N_2, CH_4, CO_2, Xe and SF_6 respectively on Gilmont flowmeter scale with a stainless steel float) with an 18-gauge needle as an outlet.

Excess $CHCl_3$: The gas flow was removed and the cap was rapidly changed for a new one with an unbroken septum. Using a syringe with a 21 gauge needle an equivalent volume of $CHCl_3$ (typically 3 mL) was carefully layered onto the PL whilst connected to the gas collection setup *via* a needle/tubing cannula, thereby also ensuring no air-locks remained and setting a start-point which was marked. The sample was stirred allowing the layers to mix and gas evolution was measured by displacement of water in an inverted Rotaflo stopcock 10 or 25 mL burette (0.1 mL graduations) in a beaker of water connected to the GC vial *via* the needle/tubing cannula.

1.0 eq. $CHCl_3$: The gas flow was removed and the cap was rapidly changed for a new one with an unbroken septum. Using a syringe with a 21 gauge needle the gas being tested was used, whilst connected to the gas collection setup *via* a needle/tubing cannula, to set a start-point and ensure no air-locks remained. After marking the start-point, 1.0 eq. $CHCl_3$ (relative to the amount of cage present, typically 46 µL) was carefully added so as not to disturb the PL before the sample was stirred allowing the $CHCl_3$ to fully mix with the PL. Gas evolution was measured by displacement of water in an inverted Rotaflo stopcock 10 or 25 mL burette (0.1 mL graduations) in a beaker of water connected to the GC vial *via* the needle/tubing cannula. Once the gas evolution had stopped a further 1.0 eq. $CHCl_3$ was added to ensure no further gas could be displaced.

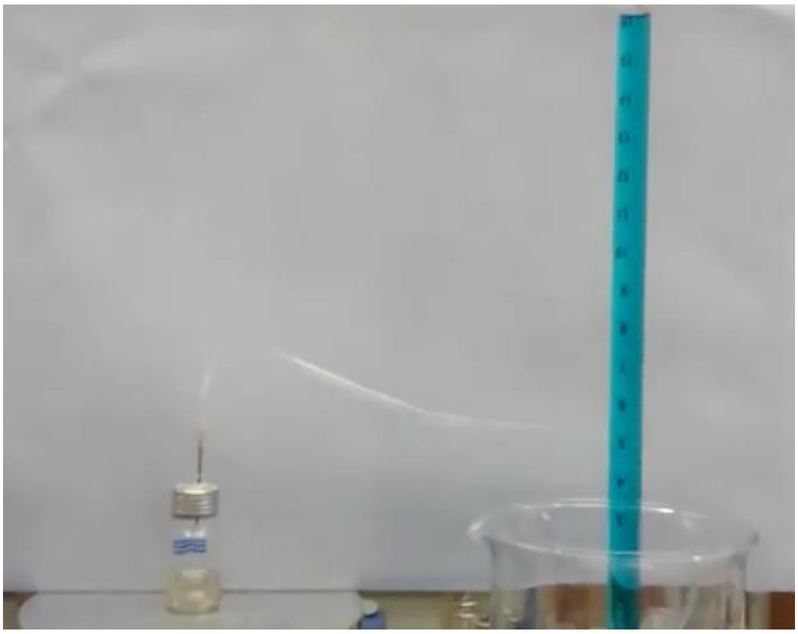

Supplementary Fig. 37: Photo of the gas collection setup used in the gas evolution studies. The sample for gas evolution studies is made up in a GC headspace vial (22 mm x 45 mm screw top, 10 mL, Fisher Scientific) and sealed with an undamaged GC screwtop cap with septum. This is connected by a needle/tubing cannula to a water filled inverted Rotaflo stopcock 10 or 25 mL burette (0.1 mL graduations) in a beaker of water. The cannula and burette were tested for leaks using an empty GC headspace vial by adding a known amount of air into the system *via* a syringe. Prior to testing a known amount of the gas in use, or the excess of chloroform, was used to set the start point and ensure no air-locks remain. After piercing the septum and removing the needle vacuum grease can be used to ensure the cap is still completely sealed and that any gas released will be solely collected in the burette.

Measurements were repeated a total of three times—two times on one batch and another on a different batch of cage material, at the same temperature to obtain a mean gas evolution and corresponding standard deviation.

Gas evolution studies of control samples *via* small solvent release:

PCP: A GC vial dried in the vacuum oven at 90 °C overnight was sealed with a septum cap and connected to a manifold *via* a needle through the septum. The vial was evacuated and refilled with N_2 before the addition of freeze-pump-thaw degassed pure PCP (2 mL) prior to gas evolution testing *via* small solvent addition (31 µL, 1.0 eq. $CHCl_3$—calculated from the amount of cage that would have been used in 2 mL i.e. 400 mg) using the standard procedure for PLs above.

PCP + Gases: Method as for PCP but the desired gas was bubbled through the solution at a rate of 50–60 mL/min for 5 min per 1 mL of PCP prior to testing (see standard procedure above for the PL).

PL + Bulky Additive: Conducted on 20% w/v PL (600 mg dissolved in 3 mL) prepared and saturated with CO_2 according to the standard procedure, but 1-*t*-butyl-3.5-dimethylbenzene (108 µL, 1.0 eq. relative to the amount of cage used) used as a test displacement solvent prior to $CHCl_3$ (46 µL, 1.0 eq. relative to the amount of cage used) using the standard procedure for gas evolution testing of the PL.

Control Non-PL + $CHCl_3$: A sample of 20% w/v control non-PL was prepared according to the standard procedure (600 mg in 3 mL) and saturated with CO_2 prior to gas evolution testing *via* small solvent addition (119 µL, 1.0 eq. $CHCl_3$ relative to the amount of cage used) using the standard procedure for gas evolution testing of the PL.

Calculation of maximum gas release based on a 1:1 stoichiometry of gas:cage:

Scrambled **3^3:13^3**-*R* cage mixture (200 mg) dissolved in 1 mL PCP equates to 0.1924 mmol of cage in 1 mL PCP based on an average MW of 1039.34**.**

By assuming a maximum occupancy of 1:1 gas to cage at 1 bar, *i.e.* that for a 20% w/v sample PL (200 mg cage in 1 mL PCP) the maximum gas uptake will be 0.1924 mmol, and by using the Ideal Gas Equation, the maximum theoretical volume of gas that could be evolved by displacement can be calculated:

$$V(m^3) = \frac{nRT}{\rho}$$

$$n(mol) = 1.924 \times 10^{-4}$$

$$R(J.K^{-1}.mol^{-1}) = 8.314$$

$$T(K) = 293$$

$$\rho(Pa) = 101325$$

Therefore, the maximum theoretical gas release will be 4.6 cm^3 per 200 mg of cage in 1 mL PCP. This equates to a **maximum of 13.8 cm^3 being evolved for a 600 mg in 3 mL sample of PL.**

Calculation of Gas Uptake:

By re-arranging the Ideal Gas Equation it is possible to convert the measured volume of gas evolved (cm^3) into n(mmol) of gas evolved per 600 mg cage *or* per 600 mg cage in 3 mL PCP *i.e.* per 5.7381 g PL (cage = 600 mg, PCP = 5.1381 g, 3 mL, measured ρ = 1.7127 g/mL (see **Supplementary Table 2**)).

$$n(mol) = \frac{V\rho}{RT}$$

This can be converted into an estimated gas uptake in terms of either mmol/g_{cage}, allowing comparison to other sorption data on solid cage species, or mmol/g_{PL} allowing comparison to uptake in alternative porous liquids.

Gas	Mean Gas Evolution ± SD cm^3	
	**Excess $CHCl_3$**	**1.0 eq. $CHCl_3$**
N_2	1.57 ± 0.08	2.10 ± 0.20
CH_4	5.60 ± 0.30	6.33 ± 0.21
CO_2	4.25 ± 0.13	7.53 ± 0.47

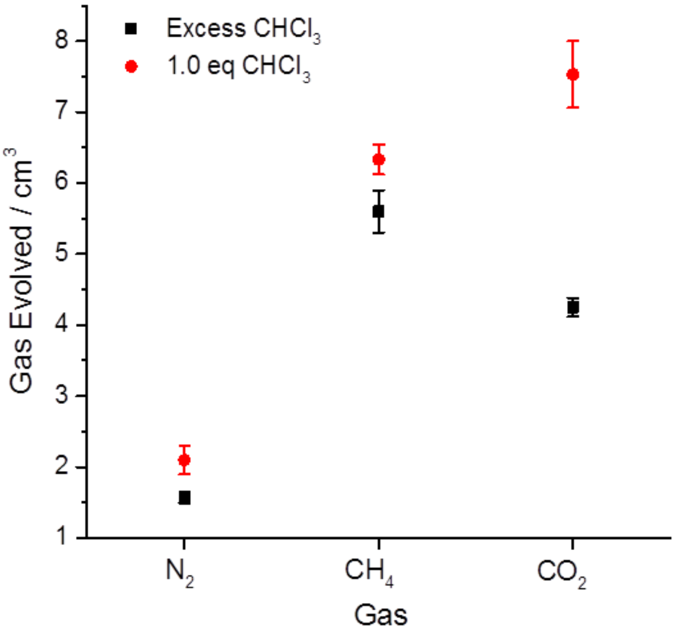

Supplementary Fig. 38: PL gas evolution measurements—average volumes of gas collected by displacement with either an excess of chloroform, or one molar equivalent based on cage. Using 1.0 eq. $CHCl_3$ proves to be more efficient in the gas evolution tests and was chosen as the optimal displacement method, presumably because large volumes of $CHCl_3$ partially dissolve the gas being displaced, which is particularly evident in the case of CO_2.

Supplementary Table 14: Volumes of gas evolved from the PL (20% w/v) and PCP using 1 molar equivalent of $CHCl_3$ relative to cage (see **Fig. 5a**) and subsequent conversion into percentage occupancies and estimated gas uptakes. Assuming a maximum occupancy of 1 gas molecule per host cage, and by using the Ideal Gas Equation, the measured amount of evolved gas can be compared to the theoretical maximum amount of evolved gas (13.8 cm^3 for the 20% w/v PL from 600 mg in 3 mL PCP) to obtain a percentage occupancy (see **Fig. 5a**). Calculation of n(μmol) gas evolved is possible from the average volume collected (cm^3) using the Ideal Gas equation, and can be converted into the uptake of each gas in terms of either μmol/g_{cage} or equivalents of gas per cage - allowing comparison to other sorption on solid cages, or μmol/g_{PL} - allowing comparison to uptake in alternative porous liquids (see **Fig. 5b**).

**Gas**	**Average Gas Evolved (cm^3)**		**Occupancy based on gas evolution (%)**	**μ(mmol) Gas Evolved** (per 600 mg cage *or* per 5.7381 g PL)	**Estimated Uptake (μmol/g_{cage})**	**Estimated Uptake (equiv.)**	**Estimated Uptake (μmol/g_{PL})**
	**PL (20% w/v)**	**PCP**					
**N_2**	2.10 ± 0.20	0.22	15	87.35 ± 8.32	145.58 ± 13.86	0.15 ± 0.01	15.22 ± 1.45
**CH_4**	6.33 ± 0.21	0.3	45	263.30 ± 8.73	438.83 ± 14.56	0.46 ± 0.02	45.89 ± 1.52
**CO_2**	7.53 ± 0.47	0.3	54	313.21 ± 19.55	522.01 ± 32.58	0.54 ± 0.03	54.58 ± 3.41
**Xe**	10.05 ± 0.21	0.45	72	418.03 ± 8.73	696.71 ± 14.56	0.72 ± 0.02	72.85 ± 1.52
**SF_6**	10.25 ± 0.07	0.45	74	426.35 ± 2.91	710.58 ± 4.85	0.74 ± 0.01	74.30 ± 0.51

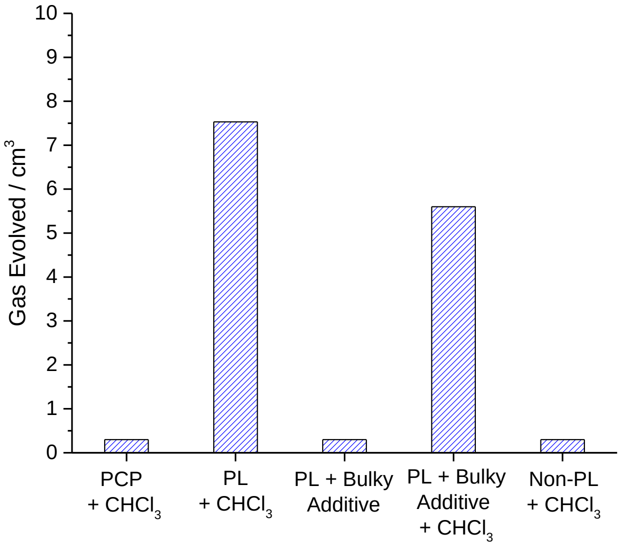

Supplementary Fig. 39: Comparison of CO_2 gas evolution measurements on CO_2-saturated control samples. Demonstrates that the cage is playing a role in the increased uptake of CO_2 (PCP *vs* PL *vs* control non-PL) and that the PL exhibits guest selectivity by size-exclusion—chloroform fits inside the cages displacing the gas, whilst the bulky additive 1-*t*-butyl-3,5-dimethylbenzene does not.

6.2 Chiral Selectivity – Enantioselective Adsorption of 1-Phenylethanol

Procedure for GC sample preparation:

Solid Scrambled 3^3:13^3-*R* cage: Six samples of solid scrambled **3^3:13^3**-*R* cage (50 mg) were desolvated at 90 °C in the vacuum oven in GC headspace vials (22 mm x 76 mm screw top, 20 mL, Fisher Scientific) before being allowed to cool to rt. To 3 of the samples was added 1 eq. of 1-PhEtOH (5.8 µL) and to the remaining samples was added 2 eq. of 1-PhEtOH (11.6 µL) before the vials were sealed.

PL: Six samples of 20% w/v PL (200 mg scrambled **3^3:13^3**-*R* cage in 1 mL PCP) were prepared according to the standard procedure in GC headspace vials (22 mm x 76 mm screw top, 20 mL, Fisher Scientific). To 3 of the samples was added 1 eq. of 1-PhEtOH relative to cage (23.2 µL) and to the remaining samples was added 2 eq. of 1-PhEtOH relative to cage (46.4 µL) before the vials were sealed. The same method was also used to prepare an equivalent six samples of 5% w/v PL (50 mg in 1 mL) with 5.8 µL and 11.6 µL of 1-PhEtOH for 1 and 2 eq. relative to cage respectively.

Non-PL: Two samples of 20% w/v control non-PL (200 mg SIC in 1 mL PCP) were prepared according to the standard procedure in GC headspace vials (22 mm x 76 mm screw top, 20 mL, Fisher Scientific). To one of these samples was added 1 eq. of 1-PhEtOH relative to the control molecule (59.5 µL) and to the other was added 2 eq. of 1-PhEtOH relative to the control molecule (119 µL) before the vials were sealed. The same method was also used to prepare an equivalent two samples of 5% w/v control non-PL (50 mg in 1 mL) with 14.8 µL and 29.7 µL of 1-PhEtOH for 1 and 2 eq. relative to the control molecule respectively.

All samples were vortexed at rt for 18 h at 200 rpm to allow them to reach equilibrium prior to the chiral selectivity measurements.

Chiral Selectivity Measurements: The enantioselectivity of the solid scrambled **3^3:13^3**-*R* cage, PL (20% w/v) and control non-PL (20% w/v) were measured for *rac*-1-phenylethanol following the previously described method.[9] Briefly, using the equilibrated samples the proportion of each enantiomer of 1-phenylethanol adsorbed in the host was measured by chiral GC analysis. The 1-phenyethanol in the chromatograms is representative of what is left in the solution phase after adsorption into the host. For example, solid scrambled **3^3:13^3**-*R* cage has adsorbed more (*S*)-1-phenylethanol than (*R*)-1-phenylethanol. This experiment was repeated at two different guest:host ratios for the solid **3^3:13^3**-*R* cage, the PL and the non-PL. Two concentrations of PL and non-PL were

tested; 5% w/v and 20% w/v. Each combination of host and guest were prepared and measured three times and repeat injections of each sample were also run to ensure reproducibility of the headspace injection. Representative chromatograms of each host with two equivalents of guest are shown in **Supplementary Fig. 40**.

The solid scrambled **3^3:13^3**-*R* cage shows chiral selectivity and an ee of 14%. The ee is consistently 14% in different samples and is the same with both 1 or 2 eq. of *rac*-1-phenyethanol. The 5% and 20% w/v PL do not show any enantioselectivity with either 1 or 2 eq. of *rac*-1-phenylethanol. Likewise the 5% and 20% w/v control non-PL doesn't show any enantioselectivity with 1 or 2 eq. of *rac*-1-phenylethanol. This suggests that the solid scrambled **3^3:13^3**-*R* cage has potential as a material for chiral separations, whereas the PL and control non-PL currently do not.

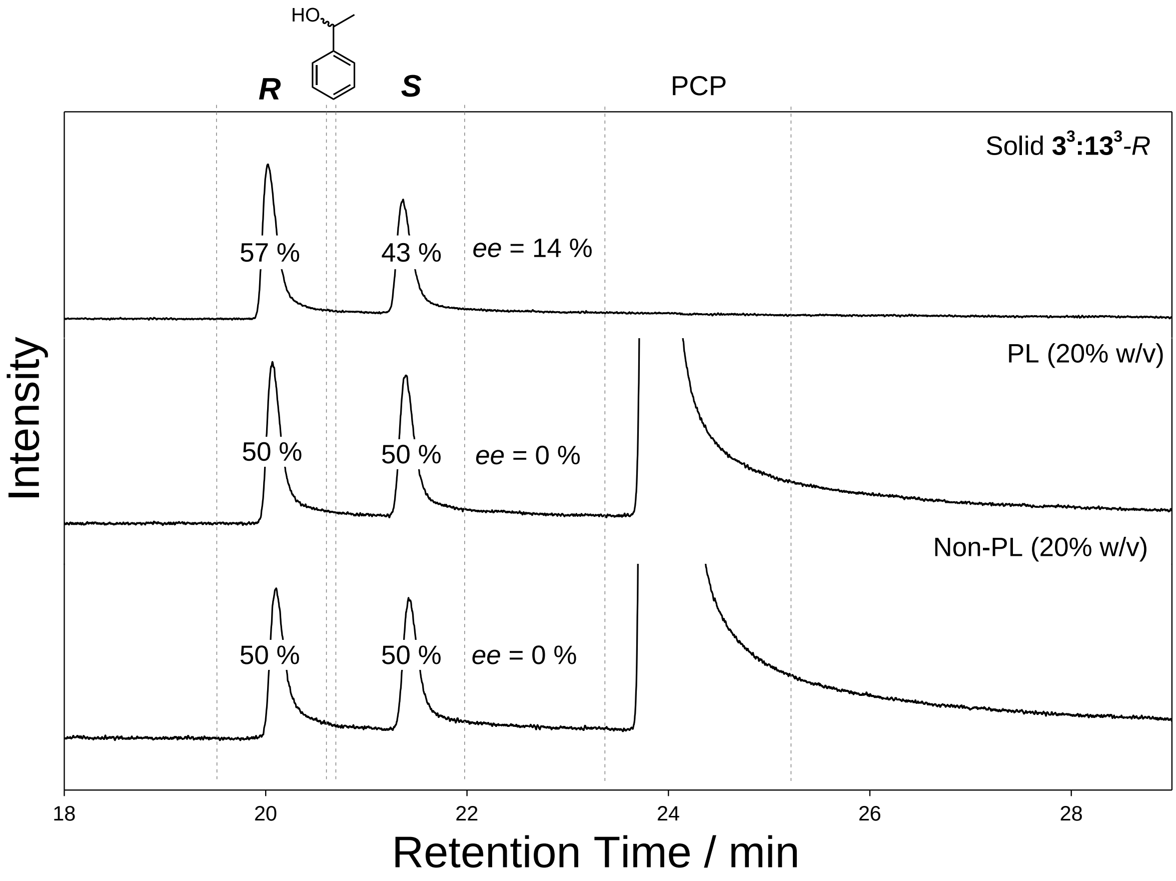

Supplementary Fig. 40: GC chromatograms of the solid scrambled **3^3:13^3**-*R* cage, the PL (20% w/v) and control non-PL (20% w/v), each with two equivalents of *rac*-1-phenylethanol.

7. Porosity in porous liquids *vs* porous organic cages

By conducting sorption of the tested gases on the solid scrambled **3^3:13^3**-*R* cage and **CC1**α, at the same temperature as the gas evolution tests on the PL (see **Fig. 7b and 7c**), it is possible to compare the gas uptakes as mmol/g_{cage} and as equivalents per cage for the two solids and the PL (20% w/v).

Supplementary Table 15: Sorption/gas evolution uptake (mmol/g_{cage}) comparison at 293 K (mmol/g_{cage}).

	**N_2** (mmol/g_{cage})	**CH_4** (mmol/g_{cage})	**CO_2** (mmol/g_{cage})	**Xe** (mmol/g_{cage})
**PL (20% w/v)**	0.1456	0.4388	0.5220	0.6967
**Scrambled 3^3:13^3-*R* cage**	0.1298	0.6094	1.5551[a]	1.7459[a]
**CC1**α	0.1950	0.7845	1.3425	1.0312

[a]Conducted at 294 K

Supplementary Table 16: Sorption/gas evolution uptake comparison of equivalents of gas per cage at 293 K (see **Fig. 7d**).

	**MW_{cage}**	**1 g Cage** (mmol)	**N_2** (Equiv.)	**CH_4** (Equiv.)	**CO_2** (Equiv.)	**Xe** (Equiv.)
**PL (20% w/v)**	1039.34	0.9621	0.15	0.46	0.54	0.72
**Scrambled 3^3:13^3-*R* cage**	1039.34	0.9621	0.13	0.63	1.61[a]	1.81[a]
**CC1**α	792.92	1.2611	0.15	0.62	1.06	0.82

[a]Conducted at 294 K

8. Diffusion NMR and Host-Guest Chemistry

8.1 Study of Cage Aggregation in the Porous Liquid

Diffusion NMR was carried out on the PL at a range of concentrations (2.5% to 20% w/v) to assess whether cages aggregate at high concentration:

Sample Preparation: Five samples of varying % w_{cage}/v_{PCP} PL (2.5%, 12.5 mg in 0.5 mL; 5%, 25 mg in 0.5 mL; 10%, 50 mg in 0.5 mL; 15%, 75 mg in 0.5 mL; 20%, 200 mg in 1 mL) were prepared according to the standard procedure in small vials and each sample was analysed by diffusion NMR and had its viscosity measured at 298 K.

Diffusion NMR: Measurements were carried out according to the general methods section using a sealed lock tube containing TMS in d_2-DCM.

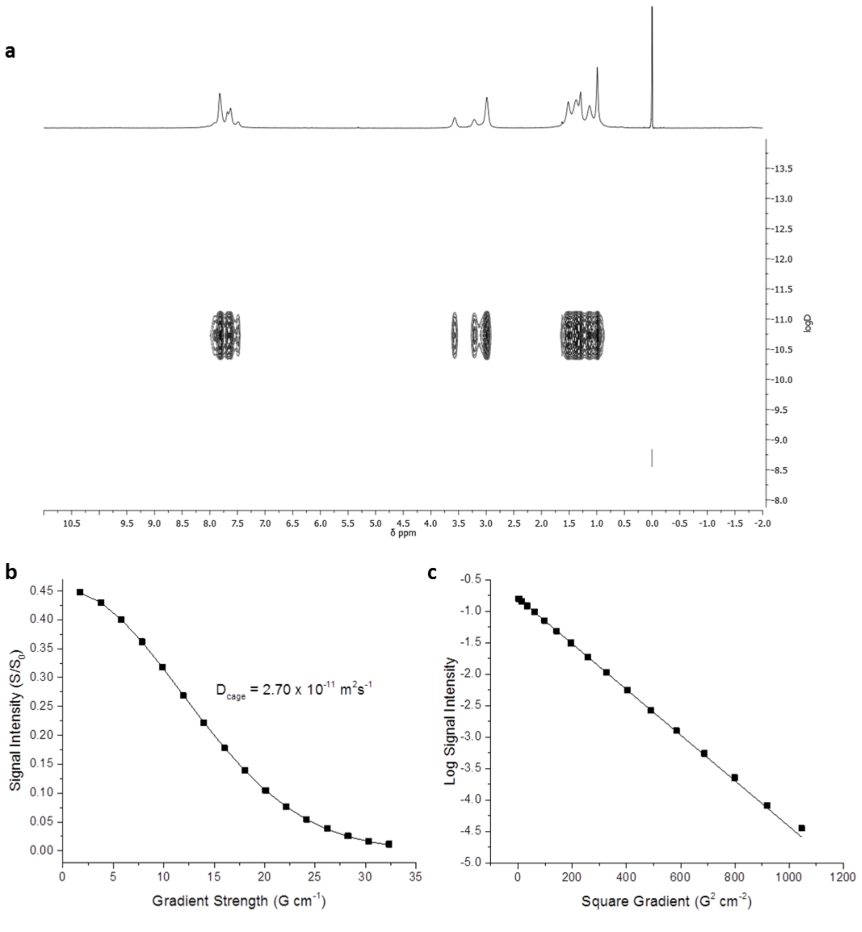

Supplementary Fig. 41: Example ^{1}H DOSY spectra (with the least attenuated spectrum [top]) for a mixture of scrambled **3^3:13^3**-*R* cage in PCP (20% w/v); (b) Attenuation of aromatic ^{1}H NMR signal with increasing gradient strength for the example shown; (c) Straight-line Stejskal-Tanner PFG-NMR response curve for the example shown. All samples were calibrated individually but measured to the same standard as shown here.

Supplementary Table 17: Summary of measured diffusion co-efficients for the scrambled $\mathbf{3^3{:}13^3}$-*R* cage in PCP at a range of concentrations (2.5–20% w/v).

Diffusion Coefficients (D) of 3^3:13^3-*R* (10^{-10} $m^2.s^{-1}$)					
**PL % w/v**	**2.5**	**5**	**10**	**15**	**20**
1	0.744	0.667	0.513	0.381	0.275
2	0.740	0.644	0.499	0.386	0.270
3	0.740	0.645	0.498	0.379	0.269
**Average**	**0.741**	**0.652**	**0.503**	**0.382**	**0.271**
SD	0.0023	0.0130	0.0084	0.0036	0.0032

Supplementary Table 18: Viscosity measurements for the scrambled $\mathbf{3^3{:}13^3}$-*R* cage in PCP at a range of concentrations (2.5–20% w/v).

Viscosity (cP)					
**PL % w/v**	**2.5**	**5**	**10**	**15**	**20**
1	3.725	4.256	5.655	7.509	10.59
2	3.734	4.252	5.651	7.494	10.6
3	3.72	4.249	5.65	7.471	10.6
**Average**	**3.73**	**4.25**	**5.65**	**7.49**	**10.60**
SD	0.0071	0.0035	0.0026	0.0191	0.0058

Supplementary Table 19: Calculated solvodynamic radii (R_s) of the scrambled $\mathbf{3^3{:}13^3}$-*R* cage in PCP at a range of concentrations (2.5–20% w/v) calculated using the Stokes-Einstein equation with the measured diffusion co-efficients and viscosities (see **Supplementary Tables 17 and 18**).

Solvodynamic Radius of 3^3:13^3-*R* (nm)					
**PL % w/v**	**2.5**	**5**	**10**	**15**	**20**
1	0.787	0.770	0.753	0.765	0.749
2	0.791	0.797	0.774	0.755	0.763
3	0.791	0.796	0.776	0.769	0.765
**Average**	**0.789**	**0.788**	**0.768**	**0.763**	**0.759**
SD	0.0025	0.0155	0.0127	0.0072	0.0089

Changes in hydrodynamic radii were found to be negligible with increasing concentration (see **Fig. 8a**), and were consistent with a cage 1.5 nm in size.

8.2 Study of the Host-Guest Chemistry in the Porous Liquid

To study the host-guest chemistry within the PL, diffusion NMR was carried out to determine the diffusion coefficients of host (scrambled **3^3:13^3**-*R* cage) and guest (CH_4, $CHCl_3$ and 1-*t*-butyl-3.5-dimethylbenzene) molecules within the PL (20% w/v). To assess whether these values correlated to guest-binding, diffusion coefficients of the same guests in neat solvent (PCP) were measured. To confirm that guest binding was a function of the cavity, and not the chemical structure of the cage, experiments were also repeated with 1,1',1''-(benzene-1,3,5-triyl)tris(*N*-cyclohexylmethan-imine) as the host in the control non-PL (20% w/v).

Sample Preparation: The following samples were made up in vials according to the standard procedures before transferring to an NMR tube for diffusion measurements:

Diffusion NMR Samples
PCP (0.5 mL) + CH_4 (2.5 min bubbling at 50–60 mL/min)
PCP (0.5 mL) + $CHCl_3$ (6.5 µL, same volume as added to PL sample)
PCP (0.5 mL) + 1-*t*-butyl-3.5-dimethylbenzene (15 µL, same volume as added to PL sample)
Non-PL (100 mg in 0.5 mL) + CH_4 (2.5 min bubbling at 50–60 mL/min)
Non-PL (100 mg in 0.5 mL) + $CHCl_3$ (16.5 µL, 0.85 eq. relative to control molecule)
Non-PL (100 mg in 0.5 mL) + 1-*t*-butyl-3.5-dimethylbenzene (39 µL, 0.85 eq. relative to control molecule)
PL (100 mg in 0.5 mL) + CH_4 (2.5 min bubbling at 50–60 mL/min)
PL (100 mg in 0.5 mL) + CH_4 + $CHCl_3$ (6.5 µL, 0.85 eq. relative to scrambled **3^3:13^3**-*R* cage)
PL (100 mg in 0.5 mL) + CH_4 + 1-*t*-butyl-3.5-dimethylbenzene (15 µL, 0.85 eq. relative to scrambled **3^3:13^3**-*R* cage)

Diffusion NMR: Measurements were carried out immediately after sample preparation according to the general methods section using a sealed lock tube containing TMS in d_2-DCM. Measurements were calibrated individually for each component in the mixed systems to accurately assess their size.

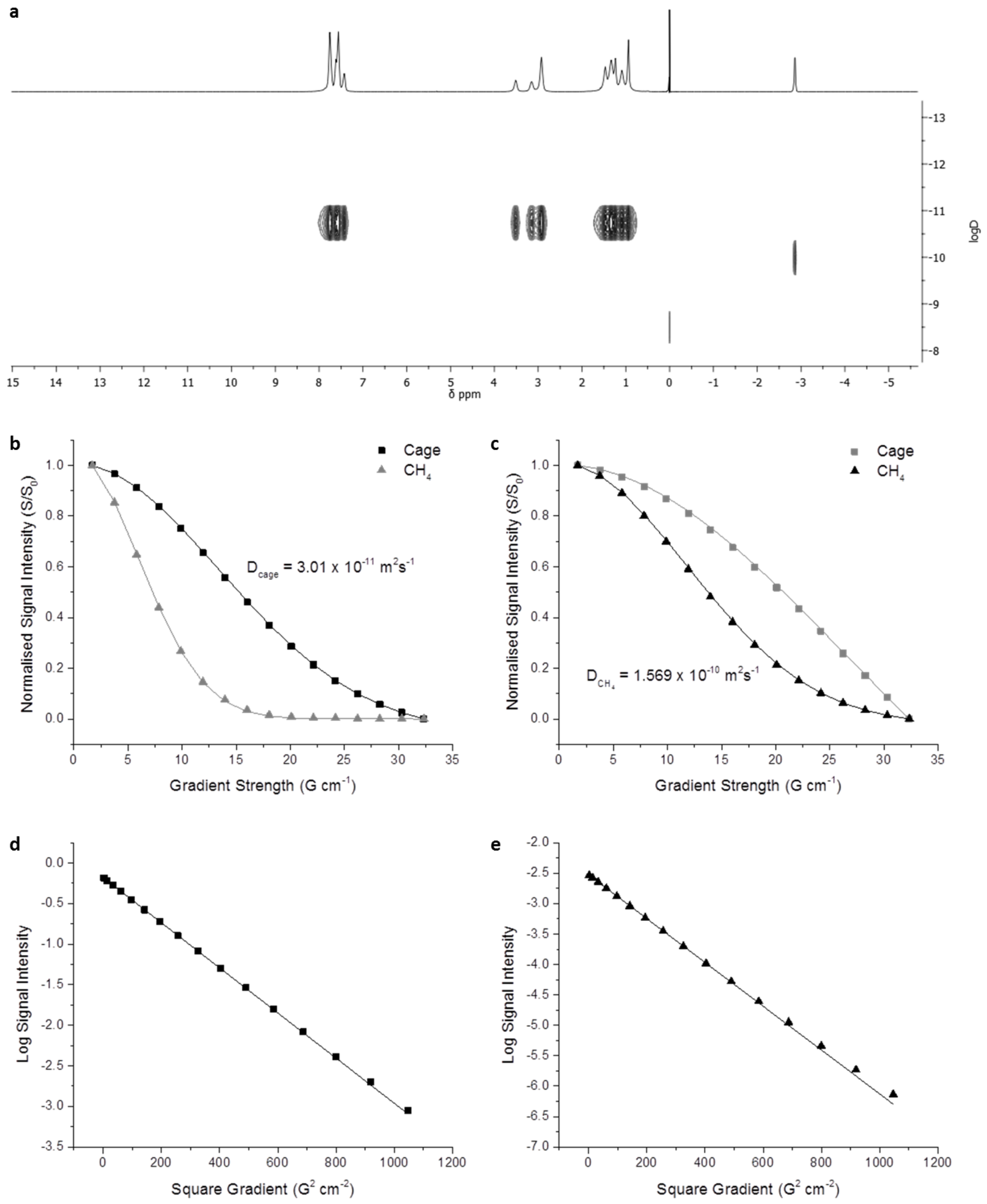

Supplementary Fig. 42: Example ^{1}H DOSY spectra (with the least attenuated spectrum [top]) for a mixture of scrambled $\mathbf{3^3{:}13^3}$-*R* cage in PCP (20% w/v) saturated with CH_4; (b) Attenuation of ^{1}H NMR signals optimised for cage molecules (squares) with increasing gradient strength for the example shown; (c) Attenuation of ^{1}H NMR signals optimised for CH_4 (triangles) with increasing gradient strength for the example shown - in each case the curve in black has been used to calculate the diffusion co-efficient of the component in this example; (d) Straight-line Stejskal-Tanner PFG-NMR response curve for the example shown corresponding to the cage molecule; (e) Straight-line Stejskal-Tanner PFG-NMR response curve for the example shown corresponding to the CH_4 molecule.

Supplementary Table 20: Summary of measured diffusion co-efficients for the guests CH_4, $CHCl_3$ and *t*Bu (1-*t*-butyl-3.5-dimethylbenzene) in PCP.

Diffusion Coefficients (D, 10^{-10} $m^2.s^{-1}$)			
**Sample**	**PCP + CH_4**	**PCP + $CHCl_3$**	**PCP + *t*Bu**
**Species**	**CH_4**	**$CHCl_3$**	***t*Bu**
1	13.88	6.323	3.258
2	14.18	6.383	3.262
3	14.58	6.37	3.261
**Average**	**14.213**	**6.359**	**3.260**
SD	0.3512	0.0316	0.0021

Supplementary Table 21: Viscosity measurements for the PCP with guest samples.

Viscosity (cP)			
**Sample**	**PCP + CH_4**	**PCP + $CHCl_3$**	**PCP + *t*Bu**
1	3.258	3.153	3.234
2	3.261	3.153	3.236
3	3.25	3.149	3.226
**Average**	**3.26**	**3.15**	**3.23**
SD	0.0057	0.0023	0.0053

Supplementary Table 22: Calculated solvodynamic radii (R_s) of the guests in PCP using the Stokes-Einstein equation with the measured diffusion co-efficients and viscosities (see **Supplementary Tables 20 and 21**).

Solvodynamic Radius (nm)			
**Sample**	**PCP + CH_4**	**PCP + $CHCl_3$**	**PCP + *t*Bu**
**Species**	**CH_4**	**$CHCl_3$**	***t*Bu**
1	0.048	0.110	0.207
2	0.047	0.109	0.207
3	0.046	0.109	0.207
**Average**	**0.047**	**0.109**	**0.207**
SD	0.0012	0.0005	0.0001

Supplementary Table 23: Summary of measured diffusion co-efficients for the guests CH_4, $CHCl_3$ and *t*Bu (1,1',1''-(benzene-1,3,5-triyl)tris(*N*-cyclohexylmethan-imine)) and SIC (small imine control molecule - (1,1',1''-(benzene-1,3,5-triyl)tris(*N*-cyclohexylmethan-imine)) in the control Non-PL (20% w/v).

Diffusion Coefficients (D, 10^{-10} $m^2.s^{-1}$)							
**Sample**	**Non-PL**	**Non-PL + CH_4**		**Non-PL + $CHCl_3$**		**Non-PL + *t*Bu**	
**Species**	**SIC**	**SIC**	**CH_4**	**SIC**	**$CHCl_3$**	**SIC**	***t*Bu**
1	0.817	0.817	10.19	0.927	4.257	0.853	2.066
2	0.807	0.82	9.998	0.926	4.257	0.852	2.053
3	0.811	0.822	10.23	0.925	4.34	0.854	2.052
**Average**	**0.812**	**0.820**	**10.139**	**0.926**	**4.285**	**0.853**	**2.057**
SD	0.0050	0.0025	0.1240	0.0010	0.0479	0.0010	0.0078

Supplementary Table 24: Viscosity measurements for the non-PL with guest samples.

Viscosity (cP)				
**Sample**	**Non-PL**	**Non-PL + CH_4**	**Non-PL + $CHCl_3$**	**Non-PL + *t*Bu**
1	6.182	6.17	5.504	6.035
2	6.194	6.154	5.511	5.975
3	6.173	6.178	5.496	5.963
**Average**	**6.18**	**6.17**	**5.50**	**5.99**
SD	0.0105	0.0122	0.0075	0.0386

Supplementary Table 25: Calculated solvodynamic radii (R_s) of the guests and control molecule (SIC) in the non-PL using the Stokes-Einstein equation with the measured diffusion co-efficients and viscosities (see **Supplementary Tables 23 and 24**).

Solvodynamic Radius (nm)							
**Sample**	**Non-PL**	**Non-PL + CH_4**		**Non-PL + $CHCl_3$**		**Non-PL + *t*Bu**	
**Species**	**SIC**	**SIC**	**CH_4**	**SIC**	**$CHCl_3$**	**SIC**	***t*Bu**
1	0.432	0.433	0.035	0.428	0.093	0.427	0.176
2	0.437	0.432	0.035	0.428	0.093	0.428	0.177
3	0.435	0.431	0.035	0.429	0.091	0.427	0.178
**Average**	**0.435**	**0.432**	**0.035**	**0.428**	**0.093**	**0.427**	**0.177**
SD	0.0027	0.0013	0.0004	0.0005	0.0010	0.0005	0.0007

Supplementary Table 26: Summary of measured diffusion co-efficients for the guests CH_4, $CHCl_3$ and *t*Bu (1,1',1''-(benzene-1,3,5-triyl)tris(*N*-cyclohexylmethan-imine)) and the host scrambled **3^3:13^3**-*R* cage in the PL (20% w/v).

Diffusion Coefficients (D, 10^{-10} $m^2.s^{-1}$)								
**Sample**	**PL + CH_4**		**PL + CH_4 + $CHCl_3$**			**PL + CH_4 + *t*Bu**		
**Species**	**Cage**	**CH_4**	**Cage**	**CH_4**	**$CHCl_3$**	**Cage**	**CH_4**	***t*Bu**
1	0.304	1.63	0.311	6.782	0.549	0.331	1.278	1.386
2	0.302	1.569	0.318	6.69	0.541	0.299	1.346	1.449
3	0.301	1.568	0.325	6.706	0.552	0.281	1.308	1.438
**Average**	**0.302**	**1.589**	**0.318**	**6.726**	**0.547**	**0.304**	**1.311**	**1.424**
SD	0.0015	0.0355	0.0070	0.0492	0.0057	0.0253	0.0341	0.0337

Supplementary Table 27: Viscosity measurements for the PL with guest samples.

Viscosity (cP)			
**Sample**	**PL + CH_4**	**PL + CH_4 + $CHCl_3$**	**PL + CH_4 + *t*Bu**
1	10.59	9.875	10.95
2	10.6	9.865	10.94
3	10.6	9.888	10.92
**Average**	**10.60**	**9.88**	**10.94**
SD	0.0058	0.0115	0.0153

Supplementary Table 28: Calculated solvodynamic radii (R_s) of the guests and scrambled **3^3:13^3**-*R* cage in the PL (20% w/v) using the Stokes-Einstein equation with the measured diffusion co-efficients and viscosities (see **Supplementary Tables 26 and 27**).

Solvodynamic Radius (nm)								
**Sample**	**PL + CH_4**		**PL + CH_4 + $CHCl_3$**			**PL + CH_4 + *t*Bu**		
**Species**	**Cage**	**CH_4**	**Cage**	**CH_4**	**$CHCl_3$**	**Cage**	**CH_4**	***t*Bu**
1	0.678	0.126	0.711	0.033	0.403	0.603	0.156	0.144
2	0.682	0.131	0.695	0.033	0.409	0.667	0.148	0.138
3	0.684	0.131	0.680	0.033	0.400	0.710	0.153	0.139
**Average**	**0.681**	**0.130**	**0.695**	**0.033**	**0.404**	**0.660**	**0.152**	**0.140**
SD	0.0034	0.0029	0.0153	0.0002	0.0042	0.0540	0.0040	0.0034

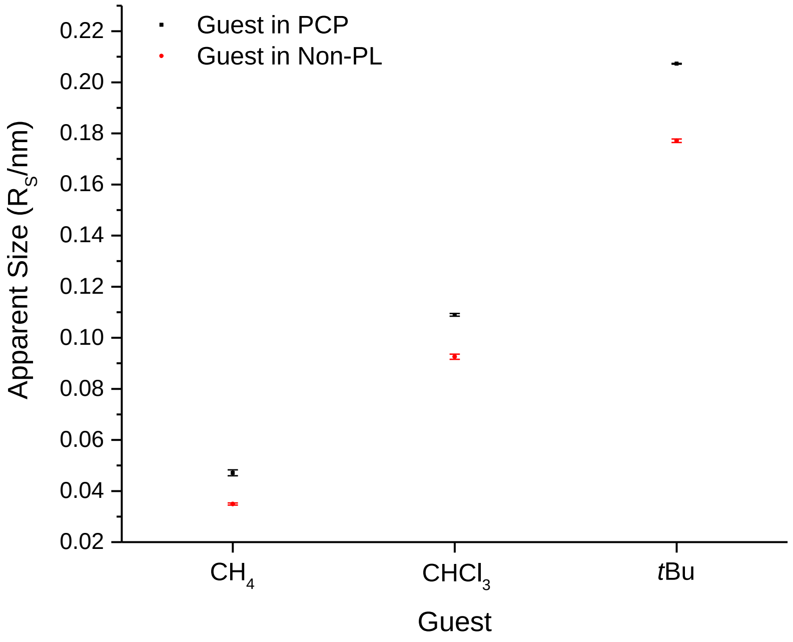

Supplementary Fig. 43: Comparison of the calculated solvodynamic radii (R_S) of the guest molecules CH_4, $CHCl_3$ and *t*Bu (1,1',1''-(benzene-1,3,5-triyl)tris(*N*-cyclohexylmethan-imine)) in both PCP (black) and the non-PL (20% w/v, red) containing the small imine control molecule (1,1',1''-(benzene-1,3,5-triyl)tris(*N*-cyclohexylmethan-imine)). No apparent increase in size is observed in the non-PL when compared to the unbound guests in PCP, suggesting no host-guest interaction.

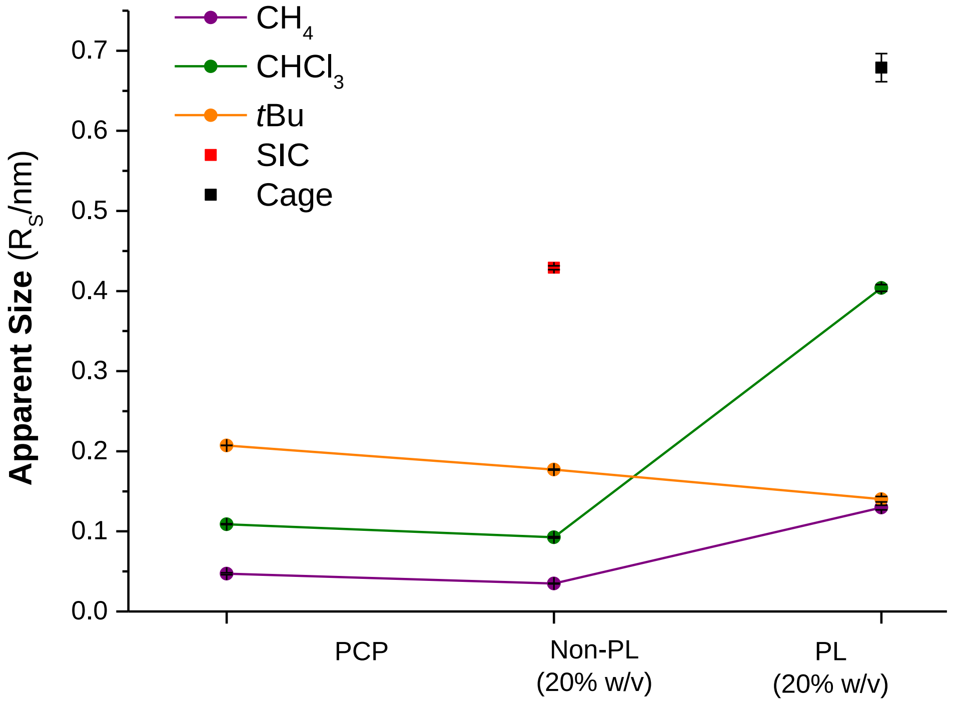

Supplementary Fig. 44: Comparison of the solvodynamic radii (R_S) of guests (CH_4, purple; $CHCl_3$, green, *t*Bu = 1-*t*-butyl-3.5-dimethylbenzene, orange) in neat solvent (PCP), and in the presence of hosts 1,1',1''-(benzene-1,3,5-triyl)tris(*N*-cyclohexylmethan-imine) (SIC = small imine control, red), and scrambled $\mathbf{3^3{:}13^3}$-*R* cage (cage, black), both in PCP (Non-PL and PL respectively, 20% w/v).

Guest uptake was established by comparing the solvodynamic radii (R_S) of guests in neat solvent (PCP), PL (20% w/v) and without the scrambled $\mathbf{3^3{:}13^3}$-*R* cage, but in the presence of a small imine control molecule in the non-PL (20% w/v). An increase in apparent R_S is consistent with a small guest forming a host-guest complex and diffusing at the speed of the host, making it appear larger. An apparent increase in size was observed for both CH_4 and $CHCl_3$ in the PL compared to their unbound state in PCP, albeit not to the same size as the host cage molecule, suggesting a host-guest complex has been formed but is dynamic in nature where the guest can rapidly exchange between its unbound and bound state. No apparent increase in size was observed for the bulky 1-(*t*-butyl)-3,5-dimethylbenzene guest suggesting it is excluded from the cage cavity. No apparent increase in size was observed for any of the guests in the control non-PL, suggesting the apparent sizes increases seen in the PL are due to the guests binding with the cage cavity.

8.2.3 Calculation of Percentage Occupancies

The estimated amount of bound guest molecules in the host cage cavities within the system (f_b) was estimated using the method previously described by Hermans *et al.*[10] To account for the viscosity difference between neat PCP and the PL, each diffusion coefficient was normalised by the viscosity.

$$fb = \frac{(D_{g,obs} \times \eta_{PL}) - (D_{g,unbound} \times \eta_{PCP})}{(D_{g,bound} \times \eta_{PL}) - (D_{g,unbound} \times \eta_{PCP})}$$

$D_{g,\ obs}$: The observed diffusion coefficient of the guest in the PL (20% w/v)

(CH_4 in PL, D = 1.589 x 10^{-10} m^2s^{-1}; $CHCl_3$ in PL, D = 0.547 x 10^{-10} m^2s^{-1})

$D_{g,\ unbound}$: The diffusion coefficient of the unbound guest, which can be approximated using the diffusion of the guest in neat PCP

(CH_4 in PCP, D = 14.213 x 10^{-10} m^2s^{-1}; $CHCl_3$ in PCP, D = 6.359 x 10^{-10} m^2s^{-1})

$D_{g,\ bound}$: The diffusion coefficient of a guest bound 100% of the time to the host cage. This can be approximated to be equivalent to the diffusion coefficient of the host cage in the PL for the sample in question

(cage in PL+CH_4, D = 0.302 x 10^{-10} m^2s^{-1}; cage in PL+$CHCl_3$, D = 0.318 x 10^{-10} m^2s^{-1})

η_{PL}: The viscosity of the PL + guest sample in question

(PL+ CH_4, η = 10.60 cP; PL+$CHCl_3$, η = 9.88 cP)

η_{PCP}: The viscosity of PCP + guest sample in question

(PCP+ CH_4, η = 3.26 cP; PCP+$CHCl_3$, η = 3.15 cP)

The estimated occupancy of CH_4 and $CHCl_3$ in the host cage cavities in the porous liquid were calculated to be 68% and 86% respectively using the stated equation.

References

1) http://webbook.nist.gov/chemistry/ — Thermophysical Properties of Fluid Systems.
2) E. O. Stejskal and J. E. Tanner, *J. Chem. Phys.,* 1965, **42**, 288.
3) Accelrys; Accelrys Software, Inc.: San Francisco, 2009.
4) C. W. Yong, *J. Chem. Inf. Model,* 2016, **56**, 1405.
5) W. Smith and T. R. Forester, *J. Mol. Graph,* 1996, **14**, 136.
6) T. R. Forester and W. Smith, *Faraday Trans.,* 1997, **93**, 3249.
7) S. Melchionna; G. Ciccotti and B. L. Holian, *Molecular Physics,* 1993, **78**, 533.
8) N. Giri; M. G. Del Pópolo; G. Melaugh; R. L. Greenaway; K. Rätzke; T. Koschine; M. F. Costa Gomes; L. Pison; A. I. Cooper and S. L. James, *Nature*, 2015, **527**, 216.
9) L. Chen; P. S. Reiss; S. Y. Chong; D. Holden; K. E. Jelfs; T. Hasell; M. A. Little; A. Kewley; M. E. Briggs; A. Stephenson; K. M. Thomas; J. A. Armstrong; J. Bell; J. Busto; R. Noel; J. Liu; D. M. Strachan; P. K. Thallapally and A. I. Cooper, *Nature Mater.,* 2014, **13**, 954.
10) T. M. Hermans; M. A. C. Broeren; N. Gomopoulos; P. van der Schoot; M. H. P. van Genderen; N. A. J. M. Sommerdijk; G. Fytas and E. W. Meijer, *Nature Nanotech.,* 2009, **4**, 721.
